# Marine exometabolites correlate with benthic fungal diversity and community structure in the South China Sea

**DOI:** 10.1128/spectrum.03557-25

**Published:** 2026-04-23

**Authors:** Xi Liu, Si-Di Ma, Jia-Ming Li, Hao Liu, Zheng-Xu Zhong, Gao-Rong Zhang, Wen-Hao Hu, Hou-Jin Li, Wen-Jian Lan

**Affiliations:** 1 State Key Laboratory of Anti-Infective Drug Discovery and Development, School of Pharmaceutical Sciences, Sun Yat-Sen University 26469 , Guangzhou, Guangdong, People’s Republic of China; 2 School of Chemical and Biological Engineering, Hechi University, Yizhou, Guangxi, People’s Republic of China; 3 School of Chemistry, Sun Yat-Sen University 26469 , Guangzhou, Guangdong, People’s Republic of China; Ocean University of China, Qingdao, Shandong, China

**Keywords:** marine exometabolites, marine benthic microorganisms, fungal community structure, fungal diversity

## Abstract

Marine exometabolites exert a substantial and dominant effect on interactions among marine benthic microorganisms. Nevertheless, the relationship between exometabolites and the community structure of benthic fungi remains unclear. In this study, we examined the abundance and diversity of fungal communities in various marine benthic habitats and elucidated their correlation with key elements, such as carbon and nitrogen, abiotic factors, such as pH, salinity, and depth, and marine exometabolites using various environmental metabolomic techniques. We found that the marine exometabolites primarily comprised organic acids and amino acids and their derivatives, which exhibited a broad negative and positive correlation with the Shannon of benthic fungal communities, respectively. Furthermore, the contribution of exometabolites to the changes in Shannon and benthic fungal community structure was higher than those of environmental factors, and organic acids exerted a highly significant impact on the fungal community structure. Key fungal taxa, such as *Alternaria* sp., showed significant association with organic acids and amino acids and their derivatives in co-occurrence networks. Structural equation modeling revealed an intuitive and concise interaction network between fungi, the environment, and marine exometabolites. Thus, the findings of this study provide new insights into the mechanisms underlying the maintenance of benthic fungal communities.

**IMPORTANCE:**

Research on the diversity and community structure of marine benthic fungi is limited. This study explores the relationships between these fungal communities and key elements (carbon and nitrogen), abiotic factors (pH, salinity, and depth), and exometabolites, with a focus on the role of marine exometabolites. The findings demonstrate that exometabolites, particularly organic acids, significantly influence the structure of benthic fungal communities. These results enhance our understanding of successional processes within these communities and clarify the interactions between benthic fungi and exometabolites, offering valuable insights into the formation of marine benthic fungal communities.

## INTRODUCTION

Benthic marine microorganisms harbor distinctive genes that facilitate the direct remineralization of organic matter and contribute substantially to the marine nutrient cycle (1, 2). Research on these microorganisms, however, has largely focused on bacteria, archaea, and protists, while fungi have received comparatively limited attention (3–5). Despite successful isolation of diverse fungal species from various benthic substrates, such as submerged wood, sediments, and macroalgae, and despite fungi constituting an estimated 5% of marine biomass carbon, their ecological functions and roles within benthic ecosystems remain poorly understood (4, 6, 7).

In the extensive marine biosphere, benthic fungi coexist with a wide range of organisms including sponges, sea anemones, crinoids, barnacles, oysters, sea squirts, and corals. They fulfill diverse ecological roles, such as decomposition, influence biogeochemical processes like denitrification, and are implicated in diseases affecting marine life (8–11). For example, 225 fungal species have been identified as causative agents of 193 disease conditions in economically important marine organisms, including crustaceans and soft corals (12). Therefore, elucidating the characteristics of marine benthic fungi and their impact on community structure is essential for advancing our understanding of geochemical processes, carbon cycling, and the sustainable cultivation of economically valuable marine species.

Marine exometabolites are a series of small molecular compounds released into the surrounding marine environment by benthic organisms. Key taxa, such as corals, algae, and sponges are prolific producers of these metabolites, which exhibit considerable structural variation and contribute to nutrient and energy transfer within benthic ecosystems. Furthermore, they support benthic biodiversity by functioning as signaling molecules that mediate ecological interactions and influence organismal behavior (13–15).

Although abiotic factors, including temperature, salinity, depth, and the availability of elements, such as nitrogen and carbon, are recognized as primary drivers of benthic fungal community assembly, the potential regulatory role of exometabolites in shaping these communities is often overlooked (16–18). Consequently, investigating how marine exometabolites influence the structure and dynamics of benthic fungal communities is essential to advance our understanding of fungal evolution, the establishment of benthic symbioses, and the development of strategies for disease prevention in commercially important marine species.

Accordingly, this study aimed to identify the primary factors driving the diversity and community composition of marine benthic fungi. To achieve this, we examined the relationship between fungal communities in various marine benthic habitats along with key elements (carbon and nitrogen), abiotic factors (pH, salinity, and depth), and marine exometabolites.

## MATERIALS AND METHODS

### Sample collection

**Fig 1:**
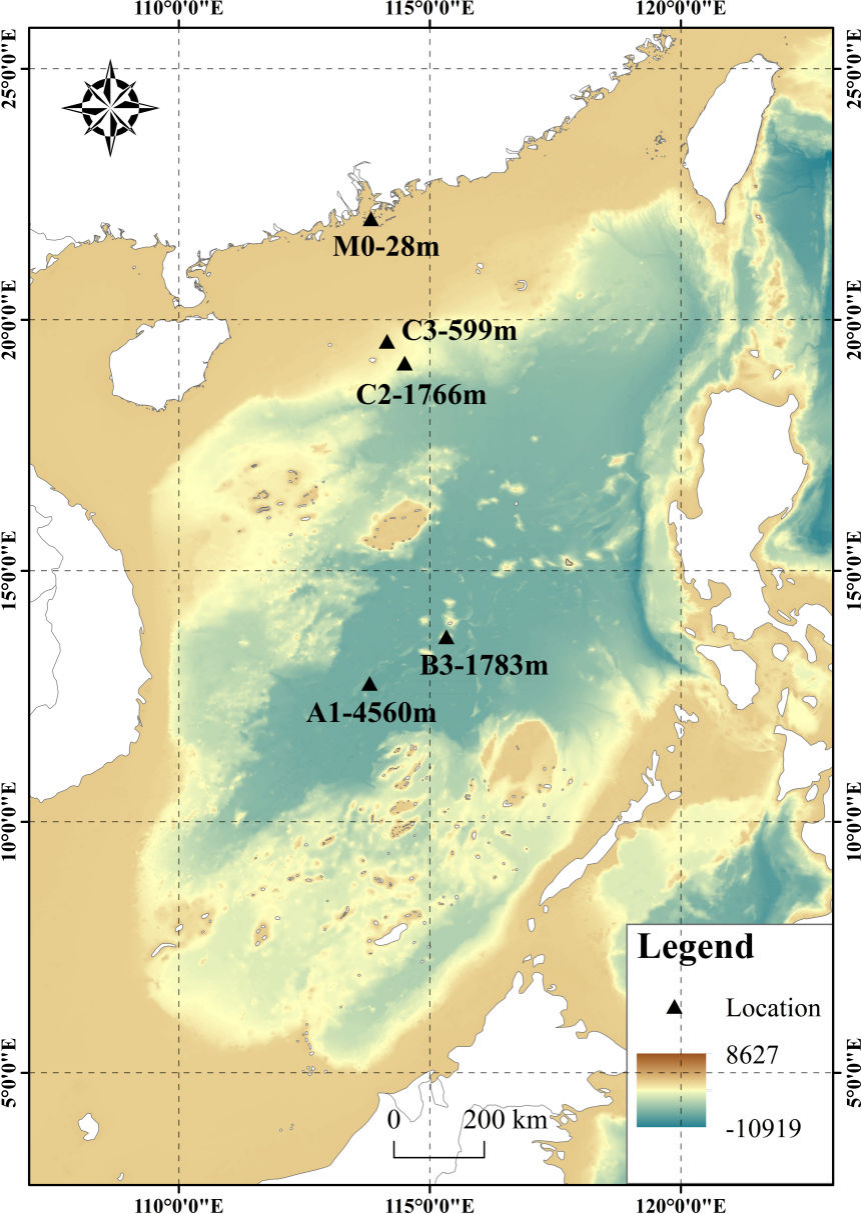
Collected location for marine sediment samples. M0: 21°55′36″ N, 113°46′6″ E, ≈−28 m, 2022.05.14; C3: 19°39′30″ N, 114°8′59″ E, ≈−599 m, 2022.06.03; C2: 19°1′19″ N, 114°25′18″ E, ≈−1,766 m, 2022.05.28; B3: 13°41′52″ N, 115°22′13″ E, ≈−1,783 m, 2022.05.21; A1: 12°58′18″ N, 113°53′21″ E, ≈−4,560 m, 2022.05.18.

Grabs were used to collect marine sediment samples from five sites in the South China Sea. The samples were immediately frozen in liquid nitrogen and stored at −80°C. The GPS coordinates of the sampling locations and sampling dates are presented in Fig. 1. The sediment cores collected from each site were randomly divided into three subsets.

### High-throughput sequencing and data processing

The samples (0.5 g) from each subset were subjected to DNA extraction using the HiPure Soil DNA Kit (or HiPure Stool DNA Kit) (Magen, China) following manufacturer protocols. PCR amplification was performed with the primers **ITS3_KYO2** (GATGA AGAAC GYAGY RAA) and **ITS4** (TCCTC CGCTT ATTGA TATGC) (19). Then, PCR products were quality-checked using 2% agarose gel, purified with AMPure XP Beads (Beckman, USA), and quantified with Qubit 3.0. Libraries were prepared using Illumina DNA Prep Kit (Illumina, USA) and qualified with ABI StepOnePlus Real-Time PCR System. Qualified libraries were sequenced on Novaseq 6000 using PE250 mode. The sequences were deposited in the Genome Sequence Archive (GSA) database of the China National Center for Bioinformation (https://www.cncb.ac.cn) (GSA Accession: CRA025811).

After sequencing, the raw data were processed using FASTP (Version 0.18.0), and the reads containing ≥10% N bases and Phred score ≤20 with ≥50% bases were removed (20). FLASH (Version 1.2.11) was used to merge the clean reads into tags with a minimum overlap of 10 bp and maximum mismatch rate of 2% (21). The tags were truncated at the first low-quality base (Q ≤ 3) in a 3-bp sliding window, and the tags with <75% high-quality segment compared with the original length were removed (22). The clean tags with 97% similarity were clustered into OTUs using the UPARSE method, and chimeras were removed using the UCHIME method using USEARCH (Version 11.0.667) (23–25). The effective tags were used for calculating OTU abundance statistics, and representative sequences were annotated based on the UNITE database (Version 10.0) using the Ribosomal Database Project (RDP) classifier (Version 2.14) (25–27). The fungal taxonomic ranking was determined based on the following similarity criteria: species, 98%; genus, 94%; family, 90%; order, 85%; class, 80%; and phylum, 75% (28).

### Analyses of marine sediment metabolites

The 15 subsamples (1 g) were placed in EP tubes and resuspended in prechilled 80% methanol by vortexing thoroughly, followed by incubation on ice for 5 min and centrifugation at 15,000 × *g* at 4°C for 15 min. The supernatant was collected and centrifuged at 15,000 × *g* at 4°C for 20 min. Finally, the solution was injected into the LC-MS/MS system for analysis (29, 30).

UHPLC-MS/MS analyses were performed using a Vanquish UHPLC system (ThermoFisher, Germany) coupled with an Orbitrap Q ExactiveTM HF mass spectrometer or Orbitrap Q ExactiveTMHF-X mass spectrometer (Thermo Fisher, Germany). The samples were injected into a Hypersil Gold column (100 × 2.1 mm, 1.9 μm) using a 12-min linear gradient at a flow rate of 0.2 mL/min. The eluents for the positive and negative polarity modes were eluent A (0.1% formic acid in water) and eluent B (methanol). The solvent gradient was set as follows: 2% B, 1.5 min; 2%–85% B, 3 min; 85%–100% B, 10 min; 100%–2% B, 10.1 min; and 2% B, 12 min. The Q ExactiveTM HF mass spectrometer was operated in positive/negative polarity mode under the following conditions: spray voltage, 3.5 kV; capillary temperature, 320°C; sheath gas flow rate, 35 psi; aux gas flow rate, 10 L/min; S-lens RF level, 60; and Aux gas heater temperature, 350°C.

The data files generated after performing UHPLC-MS/MS were processed using XCMS for peak alignment, peak picking, and quantitation for each metabolite. Based on the adduct ions and setting mass deviation to 10 ppm, these data were compared with the high-quality secondary spectrum database to identify the metabolites. The blank sample was used to eliminate the background ions, and relative peak areas were obtained by normalizing the original quantitative results using the following formula: Relative peak areas = Raw quantitative value of samples/(sum of quantitative value of samples/sum of quantitative value of QC1). Compounds with a coefficient of variation (CV) of relative peak areas in QC samples > 30% were removed (31–33).

### Analyses of environmental factors

To determine the salinity, the 15 sample subsets comprising 10 g of dried soil were sieved through a 1-mm sieve. Then, 50 mL of water was added, and the mixture was shaken for 3 min. A 30-mL aliquot of the filtrate was collected via vacuum filtration and placed in an oven at 100°C–105°C until a constant weight was achieved, which was used to calculate the salinity.

To determine the pH, 10 g of air-dried sample was weighed and passed through a 2-mm sieve, to which 25 g of CO_2_-free distilled water was added. The mixture was stirred for 1 min and allowed to stand for 30 min. The pH was measured using an electronic pH meter.

To determine the organic matter content, air-dried soil sample (0.25 g) was weighed accurately and passed through a 0.25-mm aperture sieve. To this, 10.00 mL of 0.4 mol/L potassium dichromate-sulfuric acid solution was added, followed by thorough shaking and boiling for 5 min at 170°C–180°C. Then, *O*-phenanthroline indicator was added and titrated with standard ferrous sulfate solution to calculate the organic matter content.

To determine the total carbon (TC) content, the sample was heated to >900°C in a carrier gas rich in oxygen and helium. Gas separation was performed using a temperature-programmed desorption (TPD) capture column, and the separated gases were introduced into a thermal conductivity detector (TCD) for measurement. The quantity of carbon dioxide produced was used to calculate the TC content in the sample.

Total nitrogen (TN) content was analyzed using a fully automated Kjeldahl nitrogen analyzer. The sample (5 g) was weighed and mixed with 25 mL of potassium chloride solution. After shaking at 180 r/min for 1 h, the mixture was filtered. Next, 1 mL of the soil leachate was diluted to 5 mL using potassium chloride solution, to which 2.5 mL of phenol solution and 2.5 mL of alkaline sodium hypochlorite solution were added. The mixture was allowed to stand at 20°C for 1 h, followed by the addition of 0.5 mL of a masking agent. Finally, the mixture was diluted to the mark with water, and colorimetric analysis was performed at 625 nm to calculate the ammonium nitrogen content.

### Statistical analysis

OTU rarefaction curves, rank abundance curves, and weighted and unweighted UniFrac distance matrices were generated using QIIME (Version 1.9.1) (34). Alpha-diversity analysis of the fungal communities was conducted using the vegan package (Version 2.7.2) in R.

Spearman correlation analysis was performed to examine the correlation between differential compounds. The α diversity indices, namely, Shannon index, Chao1 index, and Pielou index were applied for selection. They were calculated using SPSS software (Version 26.0) with *P* < 0.005 and |R| > 0.7. Additionally, random forest (RF) modeling was implemented using R package RandomForest (Version 2.9.0), and the relative importance of each variable was assessed based on the mean squared error (%IncMSE) (35, 36). Canonical correspondence analysis (CCA) was used to analyze the relationships between significant factors and fungal community. Furthermore, variance partitioning analysis (VPA) was performed to evaluate the relative importance of metabolic compounds and environmental variables based on the Shannon index.

OTUs with a relative abundance of >1% were selected to construct the species co-occurrence network using R package igraph (Version 0.7.1) with the parameters |R| > 0.8 and *P* < 0.005 and to calculate the topological parameters. Visualization was performed using the interactive platform Gephi (Version 9.2) (36, 37). The piecewiseSEM package in R was used to structure the partial least squares path modeling and construct the interaction relationships among fungal diversity, environmental factors, and marine exometabolites.

All statistical analyses were performed using R Studio software (Version 2025.05.1+513) unless specified otherwise.

## RESULTS

### Marine exometabolites and environmental characteristics

In total, 227 known marine exometabolites were detected in 15 subsamples, of which 179 compounds were identified in positive mode and 48 compounds in negative mode. Among these, a total of 34 compounds separately showed significant correlation with Shannon, Chao1, Pielou evenness (*P* < 0.005, |R| > 0.70) and included 16 organic acids, 5 organic amines, 3 amino acids and derivatives, and 10 other compounds. Additionally, the environmental variables of different sediment sampling sites such as salinity, pH, organic carbon content, TC, TN, and ammonium nitrogen were observed, and significant differences were found among the sampling areas (Kruskal–Wallis test, *P* < 0.05). Notably, pH and TN showed significant correlation with Shannon, Chao1, and Pielou index (*P* < 0.05, |R| > 0.55). Among the 34 compounds, five compounds (5-oxoproline, epiandrosterone, victoxinine, dehydroascorbic acid, and stearamide) showed strong correlation with pH (*P* < 0.001, |R| > 0.8), and 16 compounds (vitamin U, 9-cis-retinoic acid, epiandrosterone, 11beta-hydroxyandrosterone, indole, 3-hydroxybenzoic acid, victoxinine, tetrahydrocytisine, strongylophorin-22, dehydroascorbic acid, methylsuccinic acid, alpha-isopropylmalate, stearamide, 3-methylsalicylic acid, trans-vaccenic acid, and SM(d18:0/16:1(9Z))) exhibited extremely strong correlation with TN (*P* < 0.001, |R| > 0.8).

### Composition of marine benthic fungal communities

After the filtering and denoising processes, 428 OTUs were obtained from the 15 samples. Among these OTUs, seven known fungal phyla, namely, Ascomycota (292 OTUs, 68.2% of the total 428 OTUs), Basidiomycota (85 OTUs, 19.8%), Mortierellomycota (15 OTUs, 3.5%), Mucoromycota (8 OTUs), Glomeromycota (3 OTUs), Chytridiomycota (2 OTUs), and Rozellomycota (1 OTU) were represented (976,430 sequences) comprising 23 classes, 51 orders, 116 families, and 161 genera (Table S6).

**Fig 2:**
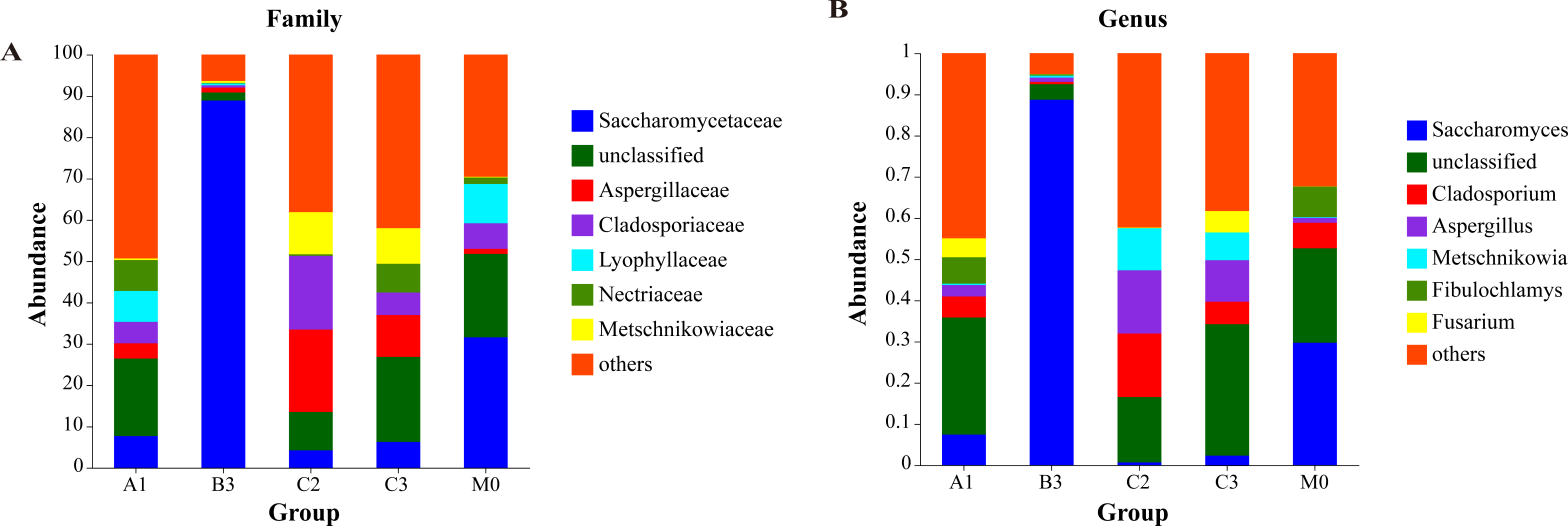
Fungal diversity analysis. (**A and B)** The relative abundance of fungi varies at different sampling points, (**A)** family; (**B)** genus.

Of these, 86.18% sequences were classified at the family and 79.75% at the genus level. In total, 161 fungal genera were identified. Of these, 10 genera—*Saccharomyces* (5.92%), *Aspergillus* (5.33%), *Cladosporium* (4.13%), *Metschnikowia* (2.81%), *Fibulochlamys* (2.19%), *Fusarium* (2.06%), *Trichoderma* (1.70%), *Mortierella* (1.40%), *Simplicillium* (1.22%), and *Coniochaeta* (1.19%)—were highly abundant and exhibited significant variation across the samples (Fig. 2A and B).

Significant differences were observed in the fungal community structure at five sampling locations. Among them, *Saccharomyces*, *Cladosporium*, and *Aspergillus* were the dominant fungal genera, and the relative abundance was significantly different in the five locations. Both Adonis analysis (Pr(>F) = 0.001, R^2^ = 0.513) and Anosim analysis (R = 0.584, *P* = 0.003) indicated significant differences, and the Kruskal–Wallis test confirmed the significant differences in the α-diversity indices in marine sediments (*P* = 0.025).

### Integrated influence of exo-metabolites and environmental factors on fungal community assembly

In total, 36 factors (34 compounds separately correlated with Shannon, Chao1 indices and Pielou evenness and two environmental factors correlated with Shannon, Chao1 indices and Pielou evenness) showed significant association with α-diversity, as evidenced by the Shannon and Chao1 indices and Pielou evenness. The Chao1 index showed significant correlations with nine compounds (*P* < 0.005, |R| > 0.70), which included four organic acids, two lipids and lipid-like compounds, lophophorine, 3,7-dimethylguanine, and three other compounds. Pielou evenness significantly correlated with 17 compounds (*P* < 0.005, |R| > 0.70), which included nine organic acids, four organic amines, two amino acids and derivatives, one lipid and lipid-like compound, and 4-hydroxybenzaldehyde. The Shannon index showed significant correlation with 21 compounds (*P* < 0.005, |R| > 0.70), which included nine organic acids, four organic amines, three steranes and sterane-like compounds, palmitoylethanolamide, nigerapyrone A, and 4-hydroxybenzaldehyde. Additionally, pH correlated negatively with the Shannon index, Chao1 index, and Pielou evenness (*P* < 0.05; R = −0.618, -0.574, and −0.618), whereas TN correlated positively with the Shannon index, Chao1 index, and Pielou evenness (*P* < 0.05; R = 0.693, 0.664, and 0.654).

**Fig 3:**
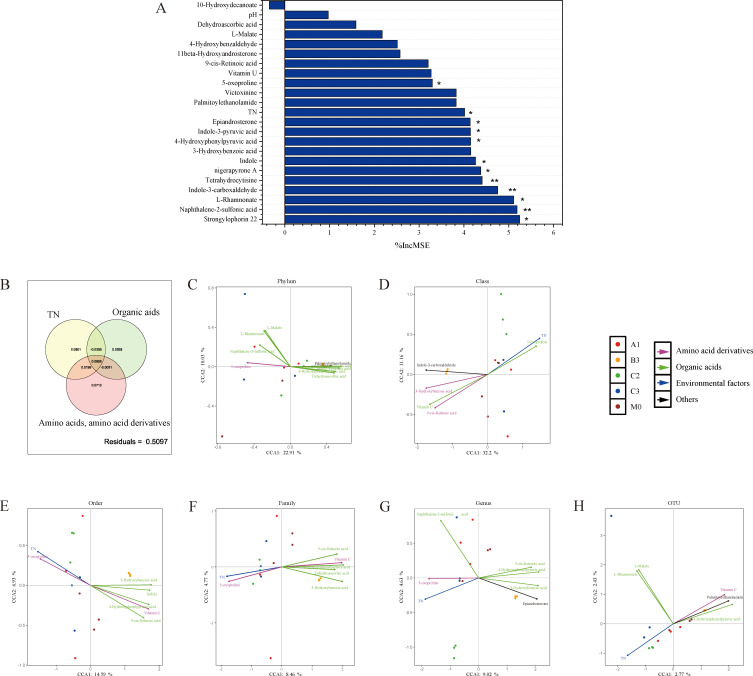
The impact of environmental factors and chemical substances on fungal diversity. (**A**) Random forest model constructed based on fungal Shannon index; (**B**) the impact of environmental factors and exo-metabolites on fungal diversity; (**C–H**) CCA analysis, (**C**) Phylum; (**D**) Class; (**E**) Order; (**F**) Family; (**G**) Genus; (**H**) OTUs.

RF model analysis of the relative importance of compounds on α-diversity based on the Shannon index (*P* < 0.05, R² = 0.858) showed that 11 compounds (%IncMSE range: 3.297–5.246) significantly influenced fungal diversity (%IncMSE.pval < 0.05). The top five compounds associated with the Shannon index were strongylophorin-22, naphthalene-2-sulfonic acid, *L*-rhamnonate, indole-3-carboxaldehyde, and tetrahydrocytisine (Fig. 3A).

Additionally, VPA analysis showed that the exometabolites and environmental factors significantly correlated with the Shannon index (Fig. 3B). Organic acids, such as 4-hydroxyphenylpyruvic acid, naphthalene-2-sulfonic acid, 3-hydroxybenzoic acid, *L*-rhamnonate, indole-3-pyruvic acid, and *L*-malate, accounted for 31.9% of variations in the Shannon index, whereas amino acids and their derivatives such as 5-oxoproline and vitamin U accounted for 18.6% and TN accounted for 16.1% of the variations. This observation indicates that organic acids exhibit the strongest explanatory power regarding the changes in the Shannon index of sediment fungal communities.

CCA was performed at the phylum, class, order, family, genus, and OTUs levels to understand the significance of the relationship between benthic metabolites and environmental factors on fungal community composition (Fig. 3C through H). The factors that significantly influenced the structure of the fungal community differed depending on the taxonomic level. Organic acid content significantly correlated with fungal community composition at the phylum, order, family, genus, and OTU levels. For example, 4-hydroxyphenylpyruvic acid exhibited significant correlations at the phylum level (Pr(>r) = 0.028 < 0.05) and at the class (r² = 0.9124, Pr(>r) = 0.001), order (r² = 0.9204, Pr(>r) = 0.001), family (r² = 0.9379, Pr(>r) = 0.001), genus (r² = 0.9392, Pr(>r) = 0.001), and OTUs (r² = 0.7035, Pr(>r) = 0.003) levels. Additionally, the amino acid derivative showed significant correlations with fungal community composition at the phylum, class, order, family, and genus levels. For example, the amino acid derivative vitamin U showed significant correlations at the phylum (Pr(>r) = 0.048 < 0.05), class (r² = 0.9255, Pr(>r) = 0.001), order (r² = 0.9255, Pr(>r) = 0.001), family (r² = 0.8918, Pr(>r) = 0.001), genus (r² = 0.8860, Pr(>r) = 0.001), and OTUs (r² = 0.5998, Pr(>r) = 0.003) levels. Other compounds, such as palmitoylethanolamide, exhibited significant correlation with fungal community composition at the class, order, family, genus, and species (Pr(>r) < 0.005) but showed weak correlations at the phylum level (Pr(>r) < 0.05). Additionally, the environmental TN concentration correlated significantly with fungal community composition at the phylum, class, order, family, genus, and OTUs levels (Pr(>r) < 0.01).

### Co-occurrence networks and factors correlated with keystone species

**Fig 4:**
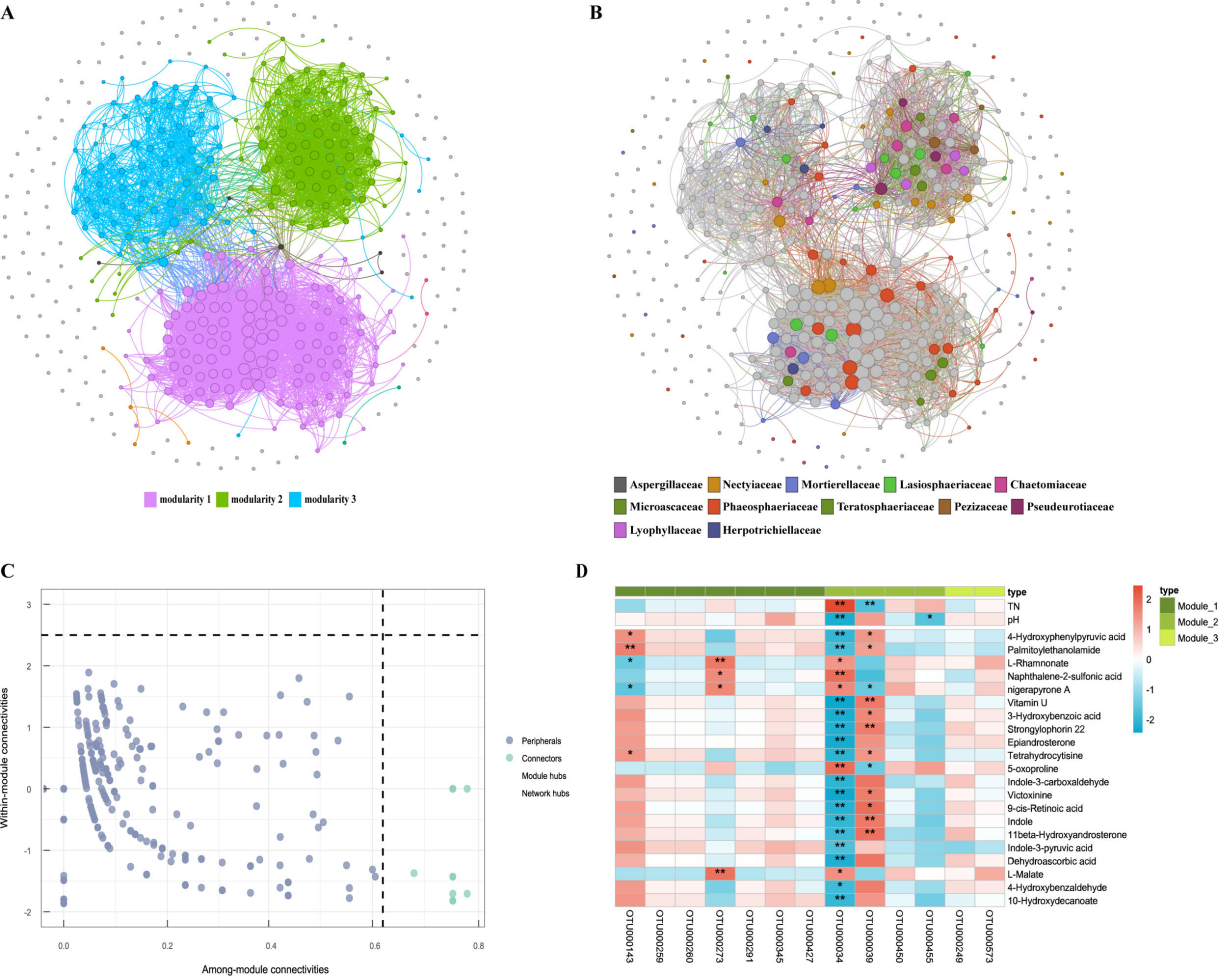
Fungal co-occurrence network. (**A**) Modular fungal co-occurrence network; (**B**) the co-occurrence network of fungal families; (**C**) within-module connectivity and among-module connectivity analysis; (**D**) correlation heatmap of multiple factors on key fungal species (*: *P* < 0.05; **: *P* < 0.01).

The fungal co-occurrence network comprised 428 nodes (OTUs) and 4,818 edges (links), and showed a diameter of 9, average clustering coefficient of 0.6437, average path length of 2.9538, and modularity index of 0.5358 (Fig. 4). The >0.4 modularity index indicates that this real-world network possesses a modular structure (38). The nodes in the network were assigned to three major modules. *Stachybotrys chartarum* OTU000039, *Alternaria* sp. OTU000034, *Lepiota venenata* OTU000450, and *Paraphoma* sp. OTU000455 dominated module 1 and co-occurred with taxa, such as Aspergillaceae, Mortierellaceae, Lasiosphaeriaceae, Phaeosphaeriaceae, Pleosporaceae, and Teratosphaeriaceae. *Candida apicola* OTU000143, OTU000259, OTU000260, *Wallemia* sp. OTU000273, *Schizophyllum commune,* OTU000291, OTU000345, and *Chordomyces antarcticus* OTU000427 dominated module 2 and were primarily associated with taxa, such as Lasiosphaeriaceae, Nectriaceae, Chaetomiaceae, Lyophyllaceae, Microascaceae, Pezizaceae, and Pseudeurotiaceae. Additionally, *Rhodotorula diobovata* OTU000249 and *Vishniacozyma victoriae* OTU000273 dominated module 3 and were primarily associated with taxa, such as Aspergillaceae, Herpotrichiellaceae, and Lasiosphaeriaceae. Thirteen species were distributed across the three major modules (module 1, module 2, and module 3) and belonged to key taxa, such as *S. chartarum* OTU000039; *Alternaria* sp. OTU000034; *L. venenata* OTU000450; *Paraphoma* sp. OTU000455; *C. apicola* OTU000143, OTU000259, OTU000260; *Wallemia* sp. OTU000273; *S. commune* OTU000291, OTU000345; *C. antarcticus* OTU000427; *R. diobovata* OTU000249; and *V. victoriae* OTU000273.

The correlation between different factors and key species varied at the species level. For example, 4-hydroxyphenylpyruvic acid, palmitoylethanolamide, and tetrahydrocytisine correlated positively with two key species (*S. chartarum* OTU000039, *C. apicola* OTU000143) but negatively with *Alternaria* sp. OTU000034. *L*-rhamnonate and nigerapyrone A correlated positively with two species (*Alternaria* sp. OTU000034, *V. victoriae* OTU000273) and negatively with *C. apicola* OTU000143. Similarly, other compounds, such as vitamin U, 3-hydroxybenzoic acid, 9-cis-retinoic acid, 5-oxoproline, and *L*-malate, modulated the abundance of different key species. Additionally, TN correlated positively with *Alternaria* sp. OTU000034 and negatively with *S. chartarum* OTU000039, and pH correlated negatively with *Alternaria* sp. OTU000034 and *Paraphoma* sp. OTU000455 (Fig. 4D).

### Ecological linkages between key exo-metabolites and fungal diversity

**Fig 5:**
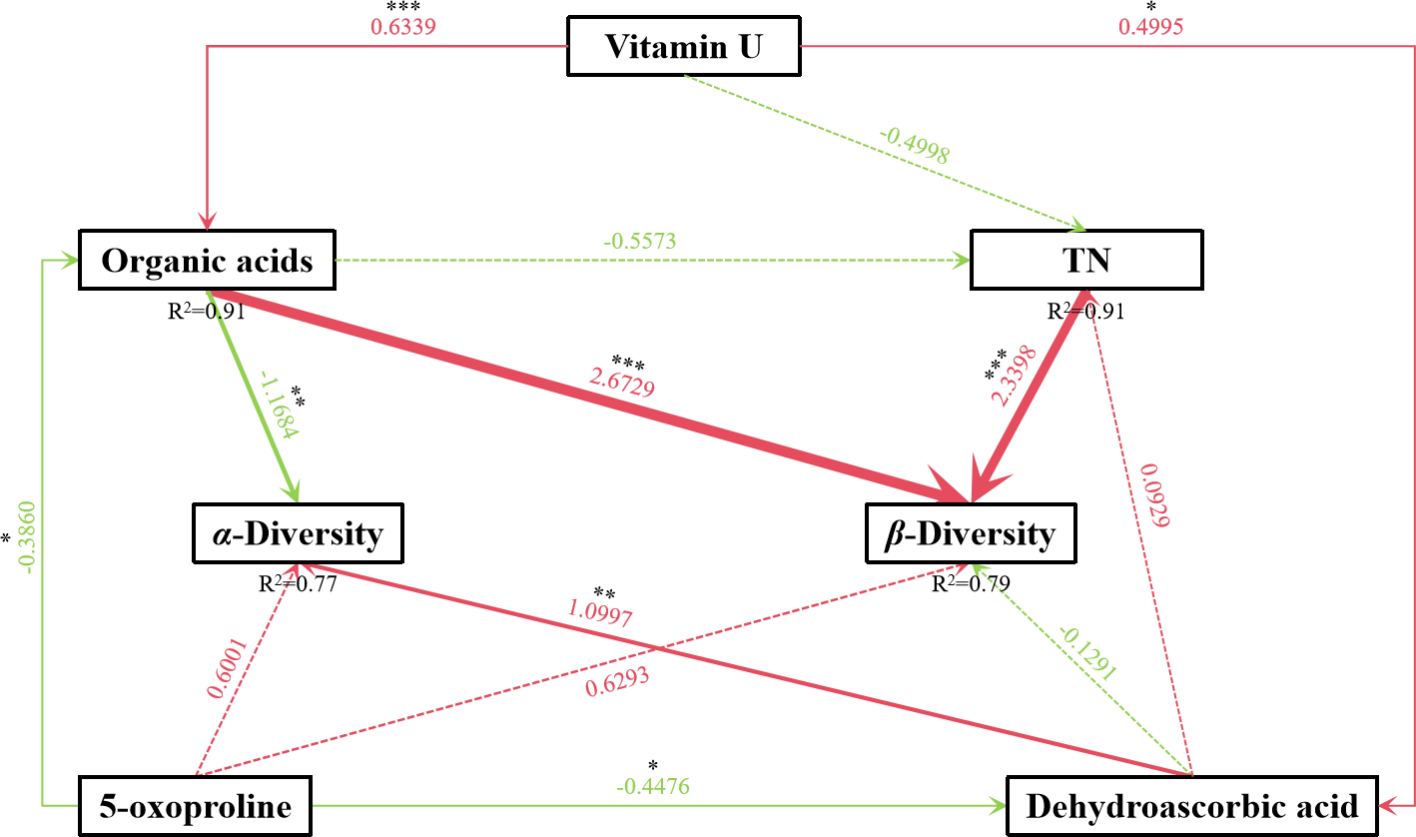
The SEM model equation is based on fungal α- and β-diversity. Red: positive correlation; Green: negative correlation; Solid line: significant correlation; Dotted line: insignificant correlation; *: *P* < 0.05; **: *P* < 0.01; ***: *P* < 0.001.

Structural equation modeling (SEM) was used to evaluate the relationship between exometabolites, α-diversity, and β-diversity of the benthic fungal communities (Fig. 5). The interactive model was a good fit for our data (AIC: 20.400; global goodness-of-fit: chi-squared = 3.326 with *P*-value = 0.767 and on 6 degrees of freedom; Fisher’s C = 6.004 with *P*-value = 0.916 and on 12 degrees of freedom). The model explained 77% of variance in α-diversity and 79% of variance in β-diversity. Organic acids showed significant negative effect on α-diversity (*P* < 0.01) and significant positive effect on β-diversity (*P* < 0.001). Additionally, TN exhibited a significant positive effect on β-diversity (*P* < 0.001), although its effect was lower than that of organic acids. Furthermore, α-diversity of benthic fungal communities correlated positively and significantly with dehydroascorbac acid, whereas organic acids correlated positively and significantly with vitamin U and negatively and significantly with 5-oxoproline.

## DISCUSSION

Marine exometabolites encompass a diverse array of compounds that facilitate nutrient and energy cycling among benthic micro-organisms. Additionally, they act as remote signals that influence the lives and behaviors of microorganisms in the proximity and contribute to biodiversity (15, 39). Establishing explicit linkages between specific exometabolites and distinct microbial assemblages is crucial for advancing our understanding of the mechanisms that sustain marine benthic ecosystems. This study investigates the relationship between exometabolites and fungal communities in the South China Sea, aiming to elucidate the role of exometabolites in regulating the diversity, composition, and co-occurrence network structures of benthic fungi. Furthermore, heterogeneity in sediment composition—including variations in grain size (e.g., soil, gravel), organic matter content, mineralogy, salinity, and specific chemical constituents—is a key driver of spatial variation in exometabolite profiles, a factor integral to the contextual framework of this research (15).

**Fig 6:**
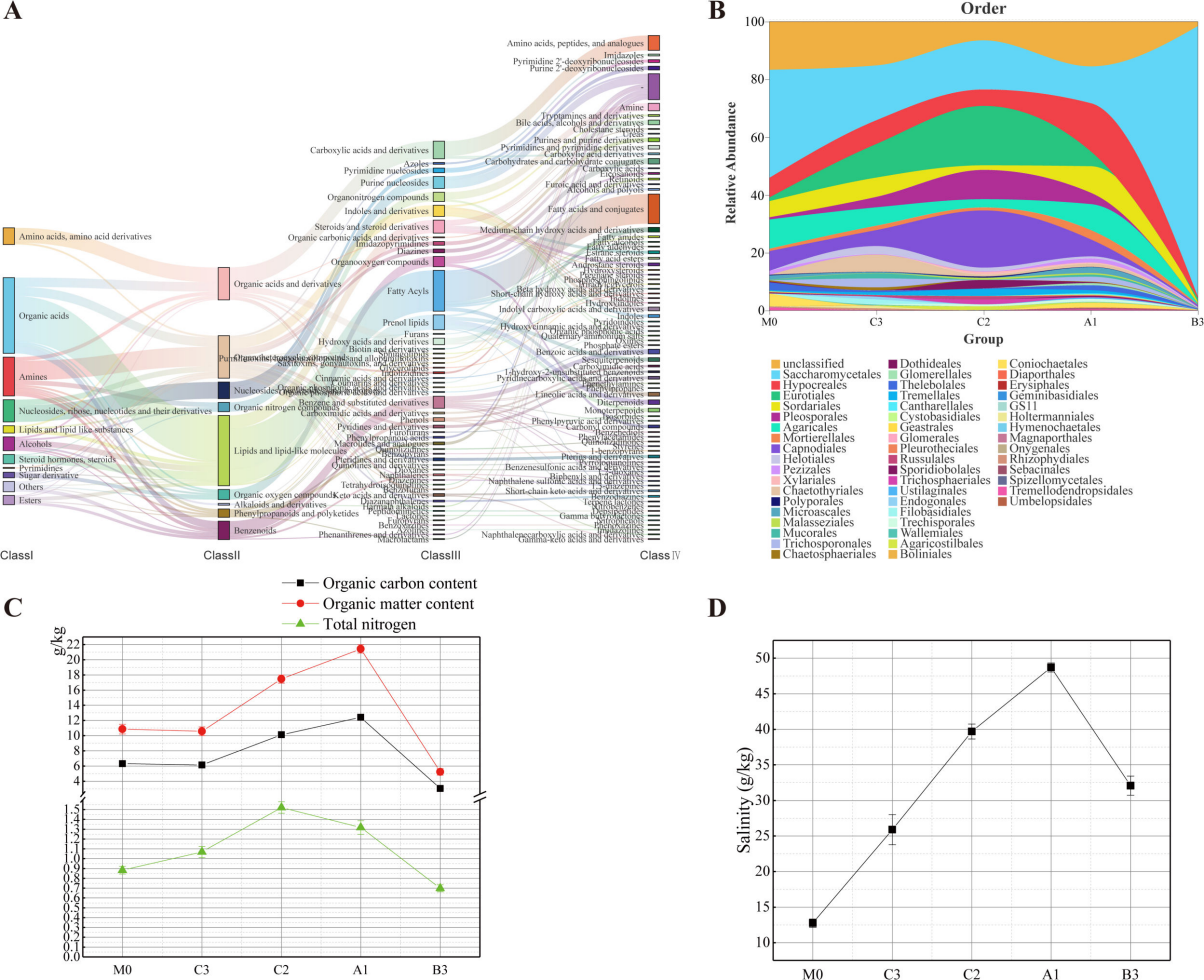
(**A**) Benthic exo-metabolite sankey diagram; (**B**) waterfall map of fungal family distribution; (**C**) line charts of organic carbon, organic matter, and total nitrogen at different collected locations; (**D**) line charts of salinity at different collected locations.

Analysis of exometabolites in the South China Sea revealed that organic acids constituted the predominant class, followed by amines (Fig. 6A). Contrary to the prevailing view that deep-sea sediments are oligotrophic (40, 41), our measurements of total nitrogen (TN), organic matter, and organic carbon content showed slightly higher levels in deep-sea regions compared with the sublittoral zone (Fig. 6C). This phenomenon may be attributed to frequent biological activities in the local deep-sea environment.

The analysis of benthic fungal communities showed that the dominant phyla, such as Ascomycota, Basidiomycota, and Mortierellomycota are widely present in marine benthic environments, which concurs with the findings of several studies (42–44). Furthermore, the relative abundance of the fungal order Hypocreales gradually increased with depth in the longitudinal range of 113°46′6″ E–114°25′18″ E (Fig. 6B). The phenomenon may be attributed to the adaptive evolution of fungi, which enables only certain species to germinate and grow under high hydrostatic pressure conditions (45–47). Distinct from other sites, the benthic fungal community at B3 was dominated by Saccharomycetales—a uniqueness attributable to its local seamount topography and currents (48). However, the mechanisms behind such seamount effects are unresolved (49). They propose dissolved inorganic nitrogen as the key driver, although they invoke this domain-specific mechanism only for bacteria and archaea. We are not sure if this factor is the main driver affecting the fungal community on seamounts, but we have also observed similar phenomena as Liu et al. (49).

Exometabolites generally exhibited a negative correlation with fungal α-diversity indices (Chao1, Shannon, and Pielou evenness). Notably, 20 organic acids were significantly negatively correlated with diversity (*P* < 0.05), suggesting a potential role in structuring these communities. Compounds such as oxalic and succinic acid can be preferentially utilized by specific taxa, enhancing their nutrient acquisition, promoting biofilm formation, and releasing substances that inhibit competitors. This process may consolidate niche dominance, thereby reducing overall diversity—a mechanism supported by prior studies (50–52). In contrast, only five metabolites showed a significant positive correlation with fungal diversity (*P* < 0.05). These included the fungal secondary metabolites *L*-rhamnose and nigerapyrone A, as well as three amino acid derivatives: *N*-lactoyl-phenylalanine, 5-oxoproline, and *L*-tryptophan. Amino acids are known to serve as readily available carbon sources in marine environments, and elevated bioavailable carbon often sustains more diverse microbial assemblages, which may explain this positive association (53, 54). Overall, while different classes of organic compounds (e.g., organic amines, lipids, carbohydrates) correlated negatively with community evenness, their consistent pattern suggests that exometabolites collectively play a key role in regulating the structure and diversity of marine benthic fungal communities (55).

Thus, organic acids most likely affect the diversity of benthic fungi in the marine benthic environment (Fig. 5). This finding concurs with the observations of Shi et al. (56) and Eilers et al. (57) that organic acids significantly influence the abundance of dominant microbial groups (56, 57). Furthermore, organic acids, amino acids, and amino acid derivatives exerted a higher effect than that of the environmental factor TN on the Shannon index of benthic fungi, in contrast to the observed widespread impact of environmental variables on microbial communities (58, 59). According to the results of the present study, the α-diversity of the marine benthic fungi is more strongly correlated with marine exometabolites than with environmental factors. However, the regulatory role of marine exometabolites on the community structure of benthic fungi remains unclear. The higher contribution of marine exometabolites to the variability of the benthic fungal community structure compared with that of TN suggests that the distribution of compounds was more critical than TN in maintaining fungal communities in marine sediments. Thus, the contribution of exometabolites to the maintenance of benthic fungal communities was likely dominant and is a primary factor that influences the variability of fungal communities.

While the role of exometabolites in marine benthic fungal networks is poorly understood, our study addresses this gap by revealing significant and specific associations: 21 exometabolites showed strong correlations (*P* < 0.001) with four key network species. These exometabolites exhibited varying associations with the four key taxa, which may be attributed to the different substrate preferences of microorganisms. Moreover, the likelihood of other taxa utilizing the substrates occupied by a particular taxon diminishes in the marine benthic niche, which is characterized by relative scarcity of resources (60).

SEM-based analysis of the mechanism underlying the effects of exometabolites and environmental variables (TN) on fungal α- and β-diversity showed that organic acids and TN exerted direct positive effect on β-diversity. Thus, organic acids and TN may increase fungal β-diversity. Additionally, organic acids showed a direct negative effect on α-diversity. Furthermore, vitamin U and 5-oxoproline showed direct positive and negative effects on organic acids, respectively. Thus, organic acids may be regulated by specific amino acids and derivatives. Moreover, dehydroascorbic acid exerted a direct positive effect on α-diversity, which indicates that dehydroascorbic acid is a key factor in maintaining the α-diversity of benthic fungal communities. To summarize, we postulate that organic acids are key factors in maintaining the diversity and community structure of benthic fungi, and other exometabolites indirectly regulate fungal communities by regulating organic acids. Although the general consensus is that key elements, such as nitrogen and carbon, are the primary driving forces that affect the structure of fungal communities, this study shows that organic acids are crucial among the variables that regulate fungal communities. Exometabolites, particularly organic acids, play an important role in fungal community succession and are important factors in shaping fungal ecological structure. These findings were derived from bioinformatic analysis, yet their experimental validation poses significant challenges. While Lai et al. conducted laboratory simulations by amending tidal flat sediments with compounds, this approach faces major obstacles in deep-sea research due to the extreme hydrostatic pressure (61). In contrast, the ichip-based method employed by Zhou et al. (62), which involves supplementing the medium in individual wells with different compounds, appears more feasible for deep-sea applications (62). Utilizing wells without exometabolites as a control group and wells with added exometabolites as the experimental group could potentially advance experimental validation in deep-sea microbial ecology. However, this method still presents significant challenges for studying deep-sea fungal communities because it requires specialized designs to protect the ichip from damage by marine organisms. Hopefully, marine microbiologists will soon overcome this challenge, allowing the study of marine fungal communities to shift from primarily bioinformatics analysis to experimental observation.

### Conclusion

Microbiome and environmental metabolomics techniques were used to identify a significant correlation between marine exometabolites and benthic fungal community diversity in the South China Sea. Exometabolites were found to be more important than key elements such as TN in elucidating the variations in the fungal community structure. Among the marine exometabolites, organic acids contributed most significantly to the structure of fungal communities. Specific fungal families showed close relation to benthic environmental depth, and the concentrations of specific compounds correlated significantly with the abundance of specific fungal taxa. SEM showed that the marine exometabolites, such as organic acids, regulated fungal community succession. Thus, the findings of this study provide new insights into the mechanisms underlying the maintenance of benthic fungal communities in the South China Sea.

## SUPPLEMENTAL MATERIAL

Supplemental material spectrum.03557-25-s0001.docx Tables S1 to S7.

ASM does not own the copyrights to Supplemental Material that may be linked to, or accessed through, an article. The authors have granted ASM a non-exclusive, world-wide license to publish the Supplemental Material files. Please contact the corresponding author directly for reuse.
