# Supplementary material for: Marine exometabolites correlate with benthic fungal diversity and community structure in the South China Sea
Source: Microbiol Spectr. 2026 Apr 23;14(6):e03557-25. doi: 10.1128/spectrum.03557-25 (PMC13227990; doi:10.1128/spectrum.03557-25)
Supplement: Supplemental material — Tables S1 to S7. [file spectrum.03557-25-s0001.docx]

**Supplementary material**

**Marine Exometabolites Correlate with Benthic Fungal Diversity and Community Structure in The South China Sea**

Xi Liu ^a,#^, Si-Di Ma ^a,#^, Jia-Ming Li ^a^, Hao Liu ^a^, Zheng-Xu Zhong ^a^, Gao-Rong Zhang ^a,c^, Wen-Hao Hu ^a^, Hou-Jin Li ^b,*^ and Wen-Jian Lan ^a,*^

^a^ State Key Laboratory of Anti-Infective Drug Discovery and Development, School of Pharmaceutical Sciences, Sun Yat-Sen University, Guangzhou, Guangdong 510006, People’s Republic of China

^b^ School of Chemistry, Sun Yat-Sen University, Guangzhou, Guangdong 510006, People’s Republic of China

^c^ School of Chemical and Biological Engineering, Hechi University, Yizhou, Guangxi 546300, People’s Republic of China

# These authors contributed equally to this work

* Correspondence:

**Wen-Jian Lan:** **Email:** lanwj@mail.sysu.edu.cn; **Tel.:** +86-020-39943042; **Fax:** +86-020-39943042

**Hou-Jin Li** : ceslhj@mail.sysu.edu.cn

**Contents**

[Table S1 Sample collection location information 3](#_Toc9385)

[Table S2 Information on Physical and Chemical Properties of Marine Sediments 4](#_Toc6124)

[Table S3 UPLC-MS compound abundance table 5](#_Toc7849)

[Table S4 Identification and classification information of compounds 15](#_Toc28307)

[Table S5 Information on OTU abundance of marine benthic fungi 30](#_Toc359)

[Table S6 OTU identification and classification information 42](#_Toc29795)

[Table S7 Alpha diversity of benthic fungal communities 54](#_Toc16248)

Table S1 Sample collection location information

| No. | Sample_name | Geographic_location | Latitude_longitude | Elevation (m) | Environment_medium |
| --- | --- | --- | --- | --- | --- |
| 1 | A1-1 | the South China Sea | 12.97 N 113.89 E | -4560 | marine sediment |
| 2 | A1-2 | the South China Sea | 12.97 N 113.89 E | -4560 | marine sediment |
| 3 | A1-3 | the South China Sea | 12.97 N 113.89 E | -4560 | marine sediment |
| 4 | B3-1 | the South China Sea | 13.70 N 115.37 E | -1783 | marine sediment |
| 5 | B3-2 | the South China Sea | 13.70 N 115.37 E | -1783 | marine sediment |
| 6 | B3-3 | the South China Sea | 13.70 N 115.37 E | -1783 | marine sediment |
| 7 | C2-1 | the South China Sea | 19.02 N 114.42 E | -1766 | marine sediment |
| 8 | C2-2 | the South China Sea | 19.02 N 114.42 E | -1766 | marine sediment |
| 9 | C2-3 | the South China Sea | 19.02 N 114.42 E | -1766 | marine sediment |
| 10 | C3-1 | the South China Sea | 19.66 N 114.15 E | -599 | marine sediment |
| 11 | C3-2 | the South China Sea | 19.66 N 114.15 E | -599 | marine sediment |
| 12 | C3-3 | the South China Sea | 19.66 N 114.15 E | -599 | marine sediment |
| 13 | M0-1 | the South China Sea | 21.93 N 113.77 E | -28 | marine sediment |
| 14 | M0-2 | the South China Sea | 21.93 N 113.77 E | -28 | marine sediment |
| 15 | M0-3 | the South China Sea | 21.93 N 113.77 E | -28 | marine sediment |

Table S2 Information on Physical and Chemical Properties of Marine Sediments

| No. | Sample name | deep (m) | Total nitrogen content (g/kg) | pH | Organic carbon content (g/kg) | Organic matter content (g/kg) | Salinity (g/kg) | Total carbon content (g/kg) | Ammonium nitrogen content (mg/kg) |
| --- | --- | --- | --- | --- | --- | --- | --- | --- | --- |
| 1 | A1-1 | -4560 | 1.35 | 8.26 | 12.2 | 21.0 | 48.7 | 16.1 | 139.3 |
| 2 | A1-2 | -4560 | 1.24 | 8.31 | 12.8 | 22.0 | 48.1 | 17 | 104.0 |
| 3 | A1-3 | -4560 | 1.36 | 8.32 | 12.3 | 21.2 | 49.3 | 16.5 | 125.5 |
| 4 | B3-1 | -1783 | 0.70 | 8.72 | 2.9 | 4.9 | 31.3 | 75.3 | 74.6 |
| 5 | B3-2 | -1783 | 0.66 | 8.73 | 2.9 | 5.0 | 31.3 | 78.2 | 60.3 |
| 6 | B3-3 | -1783 | 0.73 | 8.76 | 3.3 | 5.7 | 33.6 | 76.1 | 65.2 |
| 7 | C2-1 | -1766 | 1.58 | 8.47 | 9.8 | 16.9 | 38.9 | 41 | 81.7 |
| 8 | C2-2 | -1766 | 1.51 | 8.36 | 10.2 | 17.6 | 40.9 | 40.4 | 97.3 |
| 9 | C2-3 | -1766 | 1.47 | 8.27 | 10.4 | 17.9 | 39.3 | 39.4 | 92.7 |
| 10 | C3-1 | -599 | 1.10 | 8.76 | 6.0 | 10.3 | 25.4 | 62.2 | 79.1 |
| 11 | C3-2 | -599 | 1.10 | 8.71 | 5.9 | 10.2 | 28.2 | 59.5 | 83.7 |
| 12 | C3-3 | -599 | 1.00 | 8.67 | 6.5 | 11.2 | 24.1 | 63.1 | 93.0 |
| 13 | MO-1 | -28 | 0.91 | 8.82 | 6.2 | 10.6 | 12.9 | 8.8 | 116.9 |
| 14 | MO-2 | -28 | 0.84 | 8.84 | 6.0 | 10.4 | 12.1 | 9.2 | 108.2 |
| 15 | MO-3 | -28 | 0.90 | 8.75 | 6.7 | 11.6 | 13.3 | 7.8 | 97.7 |

Table S3 UPLC-MS compound abundance table

| Compound name | A1_1 | A1_2 | A1_3 | B3_1 | B3_2 | B3_3 | C2_1 | C2_2 | C2_3 | C3_1 | C3_2 | C3_3 | M0_1 | M0_2 | M0_3 |
| --- | --- | --- | --- | --- | --- | --- | --- | --- | --- | --- | --- | --- | --- | --- | --- |
| Norvaline | 19.4521349781827 | 15.5177695416609 | 20.7720157602303 | 20.5056215484669 | 20.3060650884666 | 20.3347976573271 | 21.6938811899452 | 27.2758357371697 | 26.6904676671121 | 18.6730391904565 | 25.7282111603456 | 27.4722517293084 | 7.18048629197294 | 6.36119454523884 | 9.38120326265991 |
| N-Lactoyl-Phenylalanine | 5.14105374922979 | 4.84226745696837 | 5.83023258626303 | 2.10539900538634 | 1.525851561 | 2.06239253201616 | 4.0434071222694 | 4.51694674821525 | 3.62198387862735 | 2.32029584944656 | 2.00568022059819 | 3.12299621146265 | 1.32166766193297 | 1.61375489309922 | 1.66332474573875 |
| N-acetylvaline | 0.0428689445028646 | 1.17466617276873 | 0.036951996751272 | 1.25454821959897 | 0.968331653953652 | 1.31512589289314 | 0.820518800869261 | 1.07579204648557 | 0.826529506562012 | 1.78637612327291 | 1.64944771823369 | 1.98488012506157 | 2.03392731752967 | 1.92306867848643 | 2.02252822409917 |
| N-acetylphenylalanine | 3.87295673238571 | 3.80727170039294 | 3.23489077346042 | 0.37950439225874 | 0.154582759115105 | 0.246672197846183 | 2.41174159150936 | 2.22353556471134 | 4.91167683257428 | 0.838523614463899 | 1.6889627675674 | 0.879013242004963 | 0.184950599399624 | 3.36090041957227 | 3.36496731552778 |
| N-acetylleucine | 2.26999276990929 | 2.64550571069801 | 2.16317284081417 | 1.25264150384374 | 1.59152428868346 | 0.9944536133671 | 1.12823530153531 | 0.916774697490579 | 1.06255842333325 | 1.02398671261188 | 1.6327577485394 | 2.45246434743404 | 1.49666529176778 | 0.634334773867936 | 3.85217174789074 |
| L-Norleucine | 65.9694261657237 | 61.41872356 | 83.9289293459238 | 83.2831959717045 | 89.1830914103505 | 84.4223093414658 | 109.700850102917 | 111.144817997222 | 85.6794550971504 | 96.093022299396 | 104.76901323908 | 119.243192154056 | 19.2226119612774 | 0.0697824754135023 | 36.7532561828418 |
| L-Isoleucine | 173.59015342088 | 157.430520957273 | 210.690077875308 | 209.730097791429 | 132.516142177389 | 202.084678093401 | 148.073456461892 | 287.360164791716 | 110.660903135328 | 269.848613850947 | 138.391803326307 | 323.529541022265 | 78.6898780530995 | 68.0454362290323 | 74.7964892144452 |
| L-Cysteic acid | 0.142445566819606 | 0.193148152164543 | 0.298687366875148 | 0.0454139949126126 | 0.0194927828564593 | 0.0904509982423394 | 0.0832859208830088 | 0.0726208238787516 | 0.0714192088613296 | 0.236466699990817 | 0.124805033249502 | 0.103025686089232 | 0.200393832973601 | 0.503720892056471 | 0.299551003864627 |
| Kainic acid | 1.59483899260523 | 2.65194208820726 | 1.93106891966359 | 0.421660104272181 | 0.438382038834012 | 0.448842750117103 | 0.754622761318801 | 1.78530519978444 | 0.86939078483962 | 0.376601147243451 | 1.68848478223963 | 0.489409452481698 | 0.130785286311081 | 0.210399183594181 | 0.139808364458004 |
| Creatinine | 0.326022730601231 | 0.24755933208225 | 0.269543467427477 | 0.143114661857742 | 0.223657908317493 | 0.19211984793818 | 1.40193542516138 | 1.44415395276003 | 0.827756055907481 | 0.158484578722149 | 0.138658747532842 | 0.203279285690239 | 0.217253183622779 | 0.196001588650751 | 0.127813695488031 |
| beta-cyano-L-Alanine | 1.64590260264952 | 2.95233954717305 | 0.18960809500445 | 3.80282705580872 | 4.63315834559417 | 0.605114810046595 | 1.13120505619693 | 1.34499806568391 | 1.57132196623136 | 2.70984517950909 | 2.93432940895256 | 5.69686975833765 | 5.74677851085935 | 4.66984977365365 | 3.40728630400806 |
| 5-oxoproline | 61.3187829164454 | 46.5500392501278 | 52.4605617617772 | 12.4915233521933 | 13.1612054349236 | 13.9060287229131 | 40.8320475346574 | 51.7193296751247 | 58.3378219491805 | 38.6313995552615 | 38.4525476509902 | 65.2121720117056 | 8.8036944004436 | 10.91529043 | 12.0172140987498 |
| 5-Hydroxylysine | 2.61947793145579 | 1.94406640753555 | 3.32319312461257 | 0.121800391792177 | 0.246002616316214 | 0.162341588906137 | 0.944516593500909 | 1.09587059670177 | 0.925727667663555 | 0.502378221575351 | 0.613076866403852 | 1.21570310653341 | 0.312969341813219 | 0.523117019596837 | 0.960072318505719 |
| Vitamin U | 85.958859262145 | 117.387112619573 | 84.6506221228188 | 242.858274996989 | 261.300978422442 | 221.463274128996 | 51.040690315621 | 59.1354131645933 | 73.5442870692496 | 120.794521388341 | 122.298175564226 | 129.695099282792 | 172.40446072212 | 148.09618384579 | 130.257761678539 |
| L-Cysteinesulfinic acid | 0.0734259126798235 | 0.103750465231503 | 0.0564604738849323 | 0.095384603146732 | 0.0762632972433253 | 0.105586230661905 | 0.0289267172345617 | 0.00613234314045347 | 0.0278631104749116 | 0.00533787949973188 | 0.00659301098978098 | 0.0661411585421323 | 0.0248023431066855 | 0.0408248231017878 | 0.00485105434664988 |
| Dopaquinone | 2.55584577091642 | 2.54945325906724 | 2.62064258816855 | 0.257879617697185 | 0.316097516919795 | 0.80951919169643 | 0.472763795129248 | 0.473260647562497 | 0.47537497868222 | 1.78740381183206 | 1.5651098682254 | 1.11353171830071 | 2.40848410543764 | 2.51763732041251 | 5.29804235686854 |
| trans-urocanate | 207.549807497007 | 201.467691395259 | 215.42736990776 | 38.046544021852 | 58.2203480613506 | 26.9706815832585 | 186.878971463534 | 214.20906527884 | 181.506466699268 | 67.475720045365 | 94.8945179165665 | 132.56664289939 | 42.0205458863907 | 49.4115112868228 | 60.739133532343 |
| Thymidine | 1.51833349209129 | 1.52826928643647 | 1.44266834954909 | 20.1443948497853 | 21.9296720069828 | 19.1225567829725 | 7.37048392601258 | 3.85345429554454 | 5.0190091187511 | 24.783035740489 | 18.426340043126 | 11.9352062102469 | 2.04149334342361 | 1.32078309392576 | 2.54981690219165 |
| N6-Methyl-2'-deoxyadenosine | 0.210387907462218 | 0.18146697517915 | 0.110832012449812 | 9.28907777393653 | 10.0322089218413 | 10.8232028060041 | 3.15407723537104 | 2.30826492456652 | 3.84879425118391 | 11.4903689643248 | 9.14957185283408 | 8.08796569544014 | 2.60805610131562 | 1.70091955223591 | 3.04049655171843 |
| inosine | 0.806201124691871 | 0.714134922944084 | 1.17411629305934 | 1.72657981278688 | 1.81815054092525 | 1.35561180241457 | 1.06712946020619 | 0.779981476157032 | 0.468497411694356 | 1.60913819270023 | 1.56989274179259 | 0.571885368430758 | 0.154988924004133 | 0.144728248652899 | 0.152242716648767 |
| Guanosine | 1.61073842750396 | 1.45835819392572 | 2.02400968383174 | 2.83720473179493 | 3.06390605696755 | 3.34150775644972 | 2.66040097329711 | 2.21144528465625 | 2.01208739252996 | 5.77261368955331 | 5.95263641225827 | 4.93059764341636 | 1.11598596338423 | 0.92291833419705 | 1.28563004572641 |
| Adenosine | 18.7800571158617 | 16.342516592024 | 22.4022246711854 | 24.8026454703296 | 27.6129061058628 | 28.7201165049282 | 14.20377063 | 10.6150417306288 | 14.5062287524412 | 52.8592332211231 | 112.375722711932 | 42.8351348042067 | 38.8176532943387 | 50.6283169764023 | 32.974700230674 |
| 3-Methyluridine | 0.0348041964296582 | 0.0520613903835738 | 0.0645170625142859 | 0.0279206788536373 | 0.175352524660982 | 0.0975588661195289 | 0.357284720044407 | 0.315155988825532 | 0.153472053843262 | 0.0881865817744962 | 0.0473775043494393 | 0.00959561557939664 | 0.0796496622796563 | 0.0519986987322903 | 0.0759068030266092 |

**Table S3** *Cont.*

| Compound name | A1_1 | A1_2 | A1_3 | B3_1 | B3_2 | B3_3 | C2_1 | C2_2 | C2_3 | C3_1 | C3_2 | C3_3 | M0_1 | M0_2 | M0_3 |
| --- | --- | --- | --- | --- | --- | --- | --- | --- | --- | --- | --- | --- | --- | --- | --- |
| 2'-O-Methyladenosine | 0.803897055201495 | 0.737459335644136 | 0.966467225243716 | 1.53370387147452 | 2.50255696298903 | 1.18115693824763 | 1.17105239629769 | 1.08080984481932 | 0.851341970144456 | 3.74113585090057 | 2.57486296900834 | 1.60787498402842 | 0.555992484586469 | 0.430850537017039 | 1.06953854415575 |
| 2'-Deoxyuridine | 0.218360483623007 | 0.206316101169561 | 0.238268447751134 | 0.926905255131446 | 0.90291872355698 | 1.00717942935937 | 1.34804578518186 | 0.859788804679826 | 0.790181740073328 | 2.7700816864769 | 2.52910630227644 | 1.27482576005747 | 0.113796241917425 | 0.0590383517546501 | 0.13705353915205 |
| 2'-Deoxyguanosine | 0.695587628346971 | 0.592743218459503 | 0.595007430927335 | 25.8311193275649 | 26.5191553798774 | 27.9927328314376 | 12.2507149309783 | 7.76157975933888 | 8.62290287490194 | 40.9421088029063 | 35.1729304845934 | 24.2621763602918 | 2.43688330054831 | 1.22644938988596 | 3.06158253188466 |
| 2'-Deoxycytidine | 0.345247838897061 | 0.246521545986258 | 0.265043021573405 | 8.04735504897433 | 8.6253935482392 | 7.69990204544004 | 4.36833862885059 | 2.95161737668516 | 3.47613679502765 | 13.8053544409464 | 11.3369580869122 | 7.31185939894172 | 0.585774111803092 | 0.263254787171599 | 0.920605615544017 |
| 2'-Deoxyadenosine | 6.86427003240422 | 6.60859354228426 | 5.37787706571397 | 117.631444394192 | 131.375866681466 | 119.370246080862 | 36.9347879801514 | 25.0504456472578 | 39.0680114394147 | 173.946818576316 | 154.830136033584 | 133.044319851562 | 20.6791408950882 | 8.91022489818513 | 32.1693023910042 |
| N,N-Dimethylguanosine | 0.404015457812977 | 1.79969930921096 | 1.10647649592755 | 0.966978912639371 | 1.86559611059174 | 0.662448516795656 | 0.228436706404206 | 0.491959839103482 | 0.0412799780607608 | 2.59165502077981 | 2.72147137302934 | 0.856974981422088 | 1.18357094596108 | 2.42611622538763 | 1.49648230983349 |
| N(6)-OH-Me-Adenosine | 0.381967013071448 | 1.13927441513804 | 0.685110192796009 | 0.832208575295796 | 1.25158728203259 | 0.70751418114938 | 0.651233881298673 | 0.470281800235715 | 0.419250984252652 | 2.38488680523147 | 2.77166703259085 | 1.34380472562783 | 0.567570026053474 | 0.941048585656403 | 0.798733645638198 |
| 9-Riburonosyladenine | 0.199747521612604 | 2.82375881244584 | 3.15890726954907 | 13.2926108761037 | 11.9717946326627 | 18.4600486783573 | 8.82294462244831 | 4.65505310866994 | 5.9223456775587 | 2.20310914702508 | 2.27200836288765 | 3.31273769764901 | 0.129030237900357 | 0.127824406803928 | 0.0988119932962218 |
| 5'-N-Methylcarboxamidoadenosine | 10.4850452844545 | 9.04072436424882 | 11.4427926789737 | 5.43706032435098 | 4.14181336194152 | 6.33228153898619 | 5.57772825716098 | 4.25006470489716 | 3.86001391656258 | 5.26832532956192 | 5.33015346513629 | 3.8588513048603 | 7.81350217293078 | 7.28650936027932 | 7.39852469138483 |
| 5'-Dehydroadenosine | 0.298426255885649 | 2.32843899837861 | 2.18372191876109 | 16.9669985737935 | 19.8462068617231 | 25.4682936953823 | 5.3246560116217 | 5.5561021918638 | 4.36477466434151 | 3.97769453512928 | 3.69506900037017 | 8.73409235174768 | 1.0652999606143 | 0.824035512928755 | 0.780152037831873 |
| 5,6-Dihydrouridine | 1.2394684441614 | 1.22846586674155 | 0.988926185390966 | 1.65017393381392 | 1.81166230292983 | 2.00020404813483 | 0.508996297428629 | 0.706016545073136 | 0.774866313398469 | 1.52796191689226 | 0.43499875464406 | 4.30606584608012 | 0.313080356119912 | 0.309201951027501 | 0.327853109488655 |
| 2'-Amino-2'-deoxyadenosine | 3.18579586064265 | 2.66431623357084 | 2.95708961999558 | 0.908756389499345 | 1.16724219450952 | 1.56348115237823 | 2.4819894439763 | 2.05118516742276 | 5.72761226934183 | 0.994763244946792 | 1.16614964828874 | 0.775305391226522 | 0.681411654853948 | 0.460923599984403 | 2.77367366908225 |
| Sphingosine (d17:1) | 0.0471301418831518 | 0.113374668935972 | 0.0553536278971124 | 0.0806588540825056 | 0.0416268058688619 | 0.0685670846596865 | 0.0761185389749116 | 0.125682415429339 | 0.245801964539577 | 0.814178458535902 | 1.10283442012991 | 4.18454420234035 | 0.441375822833335 | 0.413201804100478 | 1.95048643551663 |
| Phytosphingosine | 0.109790989570088 | 0.152747468992787 | 0.0282298980792128 | 1.0692415334637 | 2.55687422790506 | 2.70460774895981 | 0.676368067805091 | 0.84814494497535 | 0.726488692362532 | 0.818290080809463 | 0.311251629949784 | 4.85257122378598 | 0.576490422415531 | 0.56183263965148 | 0.79936306905605 |
| oleoyl ethanolamide | 0.0337306535617805 | 0.179398621406312 | 0.386845622840245 | 0.870712264812551 | 0.123086409745892 | 0.106046992283249 | 0.704385755365775 | 1.44622949772812 | 0.415417537036051 | 1.05883638852535 | 0.596450981278418 | 2.29073865676333 | 3.07484395702545 | 0.942756978969787 | 0.900534763727736 |
| N-Feruloylserotonin | 0.0962694261979062 | 0.160747630616446 | 0.0511823787537921 | 0.371807132612539 | 0.27560886364882 | 0.274304001519504 | 0.0221781569781905 | 0.0338825608921543 | 0.119401752903405 | 0.113561736599197 | 0.0448659575685031 | 0.079786856982669 | 0.154649081452975 | 0.155824671234692 | 0.150271546461316 |
| Histamine | 0.144683303861561 | 0.19468188738075 | 0.153973342136012 | 0.246730312611665 | 0.0121916749023949 | 0.0141415936324524 | 0.0430298142311128 | 0.0530842576519726 | 0.0963971543930719 | 0.137931773057388 | 0.28910995127169 | 0.123452243938345 | 0.396996462547545 | 0.312748054922165 | 0.605357382832234 |
| 2-Amino-1-phenylethanol | 59.0261677042236 | 53.8838366219127 | 80.7511406138816 | 58.7956799971231 | 64.2371723787105 | 56.3672359562946 | 91.1790761854343 | 119.977162090512 | 64.9639616968517 | 106.884055726307 | 95.5827568495715 | 153.063727039949 | 29.0525395647838 | 23.4163648356271 | 44.847602581658 |
| Xestoaminol C | 0.661460243146598 | 1.09314943581077 | 0.585204457177067 | 6.4606296749354 | 6.94732192019569 | 6.03598108265151 | 0.417080229574028 | 0.238364778617658 | 0.509201267387922 | 2.32059242013408 | 1.30512219609501 | 2.02557407493953 | 2.89668039169154 | 2.96591073121238 | 1.88742135071601 |
| Serotonin | 0.727897181147021 | 1.07681883425238 | 0.550019509038897 | 1.21322769525887 | 1.43424320364525 | 1.18119622589787 | 0.254936142935786 | 0.350420893990843 | 0.599000182783531 | 0.671680438425193 | 0.321045173856817 | 0.593547047326658 | 0.925650747348372 | 0.722063466701016 | 0.345067332937018 |
| Palmitoleoyl Ethanolamide | 6.39490377061017 | 20.4738620055325 | 7.93529390684735 | 13.1285213480065 | 31.4213562053523 | 33.3619294790146 | 16.6180475436328 | 25.3413727516403 | 16.6579894329472 | 81.4629648934262 | 45.3163718447442 | 97.3693928502167 | 59.3790460762947 | 48.7220569926677 | 70.8070434233103 |
| N,N-Dimethylaniline | 6.11110309275734 | 7.46095983507542 | 5.65963613161721 | 34.4492053028983 | 33.614854918651 | 31.1693022443236 | 6.78904989561242 | 4.12247895352705 | 5.05100104024347 | 11.4253130329822 | 5.99316014586972 | 8.57588995229527 | 10.2941097398593 | 11.916098073544 | 9.07511829059464 |
| Taurodeoxycholate | 13.523963925311 | 13.5176125943392 | 4.79505801936672 | 27.7735190689928 | 36.5924377774697 | 35.8644650308492 | 10.4717077438453 | 20.5874907492293 | 9.94110523934064 | 18.9238567860128 | 5.36283762504457 | 16.1365066285085 | 15.6629625572099 | 6.41574909724142 | 10.3035044926535 |

**Table S3** *Cont.*

| Compound name | A1_1 | A1_2 | A1_3 | B3_1 | B3_2 | B3_3 | C2_1 | C2_2 | C2_3 | C3_1 | C3_2 | C3_3 | M0_1 | M0_2 | M0_3 |
| --- | --- | --- | --- | --- | --- | --- | --- | --- | --- | --- | --- | --- | --- | --- | --- |
| Taurocholic acid | 40.1245726143795 | 45.7967399936214 | 17.9705272841376 | 189.598706345508 | 188.256420311798 | 172.016404942868 | 35.0737820132138 | 46.2232356873688 | 29.3843098000537 | 77.6742128840077 | 17.4838125500938 | 51.4690496307774 | 54.1498244299631 | 20.4635359600122 | 40.884000628638 |
| Taurochenodeoxycholic acid | 72.811091551049 | 90.0288055907093 | 29.7383280476188 | 283.398645470365 | 342.408825534037 | 333.429935222302 | 49.8561352863166 | 102.421605793242 | 54.0607872364263 | 128.172855517007 | 32.7816688806734 | 102.032230699177 | 127.336337170757 | 44.8614230233233 | 71.511323532534 |
| Glycocholic acid | 9.65716100227586 | 9.31147129216312 | 3.72667906559225 | 25.3415427123076 | 29.6790428942294 | 27.0269551038904 | 9.8474893164923 | 14.0246819934153 | 6.81696461280464 | 18.8743276635245 | 4.66681744867016 | 14.4331939136645 | 11.743704909512 | 5.08335898991522 | 9.67324431780362 |
| Glycochenodeoxycholic acid | 20.1786374318466 | 24.5033023975286 | 8.06315183818211 | 77.9908624946536 | 101.139064843468 | 94.7843289491439 | 14.3652858825851 | 29.5142644891811 | 17.9982023277102 | 37.1425272890634 | 9.65516314041072 | 30.5267088816151 | 35.5799567225269 | 13.0088932339392 | 20.5044469906409 |
| 5alpha-Cholestan-3-one | 2.31690422417985 | 97.9637618114391 | 3.55855793205537 | 19.1532129426514 | 14.0606364522946 | 24.6517360311461 | 851.179803470394 | 979.829464773814 | 0.995403234463942 | 1248.84647615812 | 651.294390480888 | 843.870253604014 | 750.626210302913 | 880.831668427931 | 193.194830202468 |
| Ureidopropionic acid | 8.62790488249047 | 13.8635509862349 | 19.5759744498799 | 0.0910733162331431 | 0.189760383026049 | 0.133730032271368 | 4.23854930784972 | 10.4049266349742 | 6.11999527143679 | 0.547513305463411 | 3.70416958556994 | 4.19734205982018 | 0.346137051727081 | 0.741280105410183 | 0.71357598010125 |
| 1,3,7-Trimethyluric acid | 0.888235282098442 | 0.999074011162889 | 1.10588714158647 | 2.21540920183347 | 1.6124252699725 | 1.83059179846932 | 0.623625312158414 | 0.449379687237542 | 0.623662733925455 | 0.337995765771952 | 0.156620429231459 | 0.192946721960736 | 0.562361112667297 | 0.475024422456664 | 0.38237237388609 |
| Xanthine | 4.47600498824134 | 5.38087892748076 | 5.23517138049661 | 1.7071377732307 | 2.13669907624479 | 1.81251635046162 | 1.89059589118309 | 2.13540773225402 | 2.13182786000927 | 1.11266732730839 | 1.47946349527862 | 1.66239029267246 | 1.75366612238262 | 1.69588514700563 | 1.56569988889965 |
| Thymine | 2.08184230546355 | 2.02824350821613 | 1.7183009794079 | 45.4887991014315 | 45.6204610434819 | 51.7185552411319 | 21.2445710177092 | 15.5391396116421 | 18.3259947587701 | 53.8862413895483 | 44.7753385469739 | 32.0028481237022 | 5.75883369519686 | 3.97132644072721 | 6.94438949799157 |
| cytosine | 1.83412417961482 | 1.57440922859975 | 1.45458852523974 | 54.4739509064114 | 61.3997498922491 | 55.0432766624698 | 13.3878328876176 | 10.2689640271942 | 17.8475819216177 | 61.571935102433 | 54.2528205623926 | 53.4781612858803 | 3.92511842393492 | 2.00052724818207 | 6.67034617241463 |
| Alloxan | 1.99268133289601 | 2.64795922831521 | 1.9094808908033 | 7.33827610276971 | 7.76423286982861 | 7.07993854564267 | 1.15005073709337 | 1.11599177221711 | 1.00607482766086 | 2.33707522738158 | 2.2781854189127 | 1.56636611184082 | 1.83726942885162 | 1.56173706334907 | 2.16947039698971 |
| 8-Chloroxanthine | 0.0258385240714881 | 0.023021321028069 | 0.00502363264250069 | 0.0875110112764307 | 0.0255956080440051 | 0.0143684978892136 | 0.0299031874501806 | 0.0210838031653772 | 0.0113524950991318 | 0.0209007388721405 | 0.016522555847902 | 0.0770554395658552 | 0.0551484831231004 | 0.0773696913798736 | 0.0737074954089389 |
| 5,6-Dihydroxyuracil | 5.11843693100446 | 4.5957796343886 | 7.15675160685076 | 0.778429008350888 | 0.592379026898762 | 0.706655039715514 | 3.83485050946268 | 3.94421125614956 | 6.07351433967865 | 1.08589452987924 | 1.56737114453417 | 1.15011470313957 | 3.84803794647786 | 3.66259706458814 | 3.74528563590263 |
| 3,7-Dimethylguanine | 4.57266481547477 | 6.88234280283538 | 8.37486459369244 | 19.2971334336029 | 28.9708848354626 | 4.9806809529624 | 2.25410773811597 | 3.23217368679133 | 4.7304141955013 | 6.508573554 | 5.90587805132833 | 8.63213289033926 | 14.2942109592955 | 9.18795932044043 | 9.70391356793434 |
| N-Cyclohexylformamide | 1.12025497533031 | 1.39648947655163 | 0.904002442130656 | 6.32638411399484 | 5.98774774427226 | 6.41918071281331 | 0.751353333687452 | 0.700272120768125 | 0.787099718968471 | 2.35968938419683 | 1.13355192247089 | 2.51225752172463 | 4.58925355611195 | 5.7626064871558 | 4.96450367274926 |
| N-Acetylmuramate | 0.428265728936648 | 0.404496292269044 | 0.487287287977336 | 0.172444721878953 | 0.136438685230907 | 0.194937877192887 | 0.507825027734417 | 0.501039092698275 | 1.28914967794045 | 0.588796598229107 | 0.392036149924885 | 0.98724548467782 | 0.4770454860466 | 0.536075865226759 | 0.643898899575319 |
| Methyl beta-D-Galactopyranoside | 3.42720312101249 | 3.61126268827946 | 3.48969131625473 | 0.1411107640222 | 0.147443472642455 | 0.392334880588168 | 0.429371442708236 | 0.726244524658852 | 0.463688444029692 | 24.9154518656753 | 234.13976450541 | 9.29017563148269 | 2.09901010041109 | 1.68792087797491 | 2.36465240944779 |
| D-Arabinono-1,4-lactone | 0.206397453289221 | 0.295572602541363 | 0.213013472757108 | 0.622348868924718 | 0.664519550814405 | 0.708582658988939 | 0.243820489803424 | 0.17417292969009 | 0.205830246827093 | 0.340664869357866 | 0.291202644074434 | 0.395677036655303 | 0.389402134042299 | 0.415299540900957 | 0.308890684051289 |
| MUCIC ACID | 0.330573466161607 | 0.256399766154724 | 0.315507877764203 | 0.324889160938029 | 0.290250934538944 | 0.725393767723868 | 0.137953219190593 | 0.129747316811486 | 0.159653523232385 | 0.119316173732744 | 0.0916232969310252 | 0.84138572618597 | 0.480372199600542 | 0.252184298458199 | 0.35923595368576 |
| D-Mannitol 1-phosphate | 0.900146185896977 | 0.719592601735122 | 1.02432910322513 | 0.298035841064251 | 0.191747669836958 | 0.291504546128748 | 0.129059364639114 | 0.112358371835842 | 0.22605126310243 | 0.0385001315226798 | 0.101299085677363 | 0.0220942118604347 | 0.0781935547396457 | 0.17525719886686 | 0.0376201654888823 |
| D-Gluconic Acid | 4.9431574850405 | 3.32620359351893 | 4.27721553033975 | 2.5722007252707 | 2.30371934506447 | 2.15208754174415 | 2.08942523863397 | 1.26366430486756 | 2.10706538706088 | 1.44883583271969 | 1.39309851469093 | 2.26074344625301 | 1.21047560221079 | 1.61695334016407 | 1.8186290503015 |
| alpha-Cyclogeraniol acetate | 0.7222102080126 | 0.853997885123348 | 0.600636717368433 | 1.40600571327655 | 1.65072408320529 | 0.32485430154672 | 0.383017148049273 | 0.50191657154198 | 0.530540771378575 | 0.639963690586432 | 0.768799443193051 | 1.26092152282341 | 1.76193082848926 | 1.08908233633946 | 0.879550853121704 |
| 1-Cyclohexenecarboxylic acid | 5.48759135806432 | 4.24778763536864 | 3.9046115053619 | 9.59827577855935 | 10.1205165513642 | 9.35409257390377 | 1.93481671535873 | 2.28757106954653 | 4.13962784767135 | 4.328923657 | 4.21937856002188 | 5.43353240507768 | 0.0732840001242997 | 4.42953421051392 | 5.69044241847701 |

**Table S3** *Cont.*

| Compound name | A1_1 | A1_2 | A1_3 | B3_1 | B3_2 | B3_3 | C2_1 | C2_2 | C2_3 | C3_1 | C3_2 | C3_3 | M0_1 | M0_2 | M0_3 |
| --- | --- | --- | --- | --- | --- | --- | --- | --- | --- | --- | --- | --- | --- | --- | --- |
| Prostaglandin E1 | 3.96244202972803 | 2.64786612034128 | 3.58629258856384 | 2.38275053760889 | 2.72516103402146 | 2.64295920733948 | 7.57656766066605 | 8.53460876377286 | 5.9791300352633 | 4.1189539511932 | 4.5506973065471 | 2.68365021853556 | 5.94268605154523 | 4.51148630260509 | 6.05233685021556 |
| all-trans-4-Oxoretinoic acid | 23.9742355849116 | 15.7320751515262 | 12.9290039933908 | 3.72699514646386 | 3.31265945491545 | 2.67453906264617 | 16.5475977349062 | 9.99660569796675 | 12.168304258338 | 5.21457053383616 | 4.22116946287943 | 11.1827656042541 | 6.96844740687169 | 23.7821906412345 | 11.4508763524218 |
| 9-cis-Retinoic acid | 1.58955166439515 | 3.05089256680254 | 1.55925670352945 | 4.80347746438242 | 5.64464189251114 | 6.23213171866712 | 1.26772075202313 | 1.77884071257769 | 1.3547898458566 | 3.4830288772132 | 3.38815219537763 | 2.74654340428615 | 4.22747129140147 | 2.7680031315098 | 4.86028842537696 |
| 2,5-Furandicarboxylic acid | 0.53699771428538 | 0.602561358100066 | 0.661763439725337 | 1.77043729236608 | 1.69225570038811 | 2.0756248451415 | 0.612126716152357 | 0.633734399227659 | 0.683955467385239 | 1.21031011044845 | 0.926932801930427 | 0.0233004358732654 | 3.58606193827423 | 3.59949680990404 | 3.31648506811886 |
| 13,14-Dihydro-15-keto-PGE2 | 1.40377195768339 | 1.4948231037274 | 1.63917440409406 | 2.32492763026193 | 5.96620876716706 | 2.25652118200441 | 2.33353800337994 | 2.04901901974665 | 3.56618589173292 | 2.43043697475554 | 1.86113180315948 | 2.5335298287804 | 2.72998691472732 | 2.62645651583158 | 2.69668016895644 |
| Vitamin B5 | 2.0172897826262 | 1.77282361828766 | 2.27900542613686 | 0.340944322252712 | 0.421674077237427 | 0.392090102613253 | 1.32175052049436 | 1.34956147817748 | 1.02528793583853 | 1.41648515519856 | 3.32590895747973 | 1.02710668270894 | 1.01123865123716 | 1.15172160372691 | 1.00000442786366 |
| Undecylenic acid | 3.01509378440424 | 3.78420812856824 | 4.16716698501293 | 3.4746593352914 | 3.67494329520449 | 3.3970291349666 | 2.07982947850593 | 1.88534675465169 | 2.74642024280019 | 2.97008406417822 | 4.21411682702273 | 2.60832937416903 | 3.2780738529308 | 3.84631979403049 | 4.03855806673948 |
| Traumatic Acid | 5.96811008980075 | 6.71813855431949 | 6.14981614291432 | 11.0287474275532 | 10.8408269238294 | 9.18891369977422 | 8.69569141098324 | 6.47664222215595 | 7.92329634259314 | 7.41475593709253 | 5.37764024726715 | 6.32513795186668 | 6.63112365000692 | 5.9135748618748 | 5.90145727943835 |
| trans-vaccenic acid | 0.0939633682998944 | 0.165912111903017 | 0.0885636937888588 | 0.541659879944374 | 0.56143190050312 | 0.516104595098438 | 0.0587809130861639 | 0.114724221146028 | 0.0949561341611161 | 0.238246455478877 | 0.201703530417714 | 0.294420670859674 | 0.312171147268032 | 0.242908387239845 | 0.262993418811775 |
| Tetradecanoic acid | 0.311248631118133 | 0.54277712890447 | 0.34537729866989 | 0.972796598062357 | 1.72022192096307 | 1.58031066396125 | 0.208632292454301 | 0.485641548240501 | 0.394707182947928 | 0.841505333386104 | 0.598662696477359 | 1.06926348603195 | 1.06030284648463 | 0.878665053009042 | 1.18337749351849 |
| Suberic acid | 55.9241074566437 | 50.5248322495326 | 62.1728790622379 | 35.4351265257675 | 37.7228277727513 | 41.208622308029 | 48.5030769289354 | 45.7665493159852 | 40.6361311476083 | 24.3024981168566 | 26.0107372406545 | 32.7125196129089 | 26.1729428736615 | 29.9672277267353 | 28.6049523463118 |
| Sebacic acid | 25.9252912606687 | 23.1856580973955 | 25.1976215716767 | 29.3719640466344 | 31.9048976676963 | 36.6002648292427 | 24.942342679148 | 23.4448430960324 | 24.0651318003414 | 37.0346346872596 | 19.9663299149202 | 84.8628069492665 | 13.7118575483857 | 16.0484229396192 | 16.2942242208658 |
| Sabinic acid | 5.24074806564391 | 4.87757320571429 | 4.15085768438518 | 4.7540517674426 | 3.70820601720519 | 4.11407714558972 | 2.94308103918147 | 2.74156632468488 | 3.24776242922831 | 4.18580885754557 | 6.07479785422666 | 4.26199068871496 | 4.31660606310196 | 5.39156623914374 | 6.11768673230484 |
| Ricinoleic acid | 11.3322136807096 | 19.5388834712912 | 19.8830589835783 | 54.079353372063 | 96.6940154104123 | 81.0750330316286 | 11.8081076283471 | 18.3627409217145 | 22.6319203364486 | 29.1697927173631 | 35.21831942 | 23.8984310144775 | 8.96094707364014 | 10.6190273802496 | 14.4555422137145 |
| Potassium | 3.85519191148727 | 4.51642202512229 | 4.40829353412162 | 8.12150944187992 | 7.85089330924083 | 7.88532757088958 | 3.3595668827082 | 3.56338797938568 | 3.61483398035915 | 3.84780364738892 | 2.97463753748083 | 5.15015838643292 | 4.11624828519903 | 0.13316647137486 | 4.67972528278451 |
| Pimelic acid | 13.9627436614566 | 14.0850452159862 | 15.5606419294169 | 11.5945050092837 | 11.0759154732315 | 13.0686646307101 | 10.2437417354176 | 10.404134128662 | 9.23476081509639 | 8.15441649504665 | 6.76514123725149 | 8.59144873774831 | 11.5554296289116 | 11.8689365268869 | 9.59121355579356 |
| Panthenol | 43.144719639644 | 37.5656692656871 | 36.5392410476235 | 31.919020578341 | 26.4428183127426 | 21.7899170342165 | 16.6204108866059 | 17.9158977362261 | 29.1799572203908 | 101.053311320278 | 56.684635872294 | 104.275951163486 | 29.7380481704814 | 50.59234675 | 52.4082753998908 |
| Palmitoleic acid | 1 | 1.49351421325264 | 1.17305551796167 | 1.71566223937111 | 4.2511067865731 | 3.40077676977059 | 1.01249241054275 | 3.19970677718377 | 2.30834453699172 | 5.00694223670162 | 4.62993218128368 | 6.5710919125488 | 3.43820261960689 | 1.74319380389153 | 2.34146068415669 |
| Methylsuccinic acid | 2.19938242061006 | 2.69281172818808 | 1.83956563059542 | 3.45363956726017 | 3.24898268463803 | 3.43785896372902 | 1.1475382230102 | 0.736616817451238 | 1.3472118020215 | 2.05140724012731 | 2.54674166741153 | 2.63943324445124 | 5.24040876761495 | 4.43641137283912 | 4.8924211738408 |
| L-Rhamnonate | 6.6967374157733 | 5.98582128807686 | 7.92409669434162 | 0.681511158173791 | 0.575171221819495 | 0.890642640140363 | 4.57976391020731 | 9.659452412 | 7.26434124943691 | 38.7095338182025 | 468.797068834674 | 30.3738048554624 | 1.7360438161471 | 1.76731828170842 | 2.58040157656706 |
| Hexadecanedioic acid | 28.857109862587 | 31.833358660687 | 29.2866534137723 | 86.3029202681106 | 95.7982768461068 | 174.474777859444 | 38.0852043744931 | 31.5521602686833 | 34.5585955226474 | 65.4241808962418 | 54.0689260182994 | 31.4384578001231 | 53.0729965032657 | 43.3035289629426 | 62.9288655659013 |
| Hendecanoic acid | 0.700353271575946 | 0.757463354913188 | 0.901675500153731 | 0.995878263655507 | 1.20603681716827 | 1.00380208677155 | 4.00517503948627 | 3.73472841507618 | 7.56978734511347 | 0.859884319202897 | 0.546051358414171 | 0.808818384178067 | 0.869698089522143 | 1.00754469838369 | 1.10018494425835 |
| Elaidic acid | 0.350270410771406 | 0.488911723966071 | 0.465358499245381 | 1.978430635 | 1.47436876070809 | 1.38291102047826 | 0.283517389591429 | 0.397043823737956 | 0.341125705451189 | 1.59308100144992 | 0.789259282582717 | 1.15877430759265 | 2.05248574282829 | 0.778232800450985 | 0.690375347461832 |

**Table S3** *Cont.*

| Compound name | A1_1 | A1_2 | A1_3 | B3_1 | B3_2 | B3_3 | C2_1 | C2_2 | C2_3 | C3_1 | C3_2 | C3_3 | M0_1 | M0_2 | M0_3 |
| --- | --- | --- | --- | --- | --- | --- | --- | --- | --- | --- | --- | --- | --- | --- | --- |
| Dodecanoic acid | 1.35261567185825 | 1.41160018894671 | 1.5646623587924 | 0.79799493901651 | 0.867725197055702 | 0.600343711679001 | 14.3747505779291 | 11.7823075015726 | 41.0554764505733 | 1.15144276662404 | 2.01249861168508 | 1.69594089733936 | 2.13521223719723 | 1.38292079572884 | 3.15473773247318 |
| Dodecanedioic acid | 8.89005457391558 | 9.19107446778841 | 9.77919955122327 | 12.107093292387 | 10.7026024228283 | 9.46593511881075 | 10.32758081 | 9.87310807289401 | 10.6083150710228 | 10.0009150348271 | 7.41667608316843 | 8.26935944169893 | 15.0839221789321 | 8.35769091662266 | 12.3716725250814 |
| Docosanoic acid | 0.066881144332406 | 0.0919344970050341 | 0.0497757343103882 | 0.407549595727737 | 0.422552596243594 | 0.235029573434454 | 0.0286994422846683 | 0.0420178256508114 | 0.0539219189181538 | 0.0570251128623789 | 0.0449840588288683 | 0.105487198184018 | 0.129359938219911 | 0.0904552326718693 | 0.0946787298042704 |
| Dethiobiotin | 3.01811138187534 | 2.06834107957733 | 2.56190978338272 | 2.80266028724141 | 0.805223382008093 | 2.48990526426118 | 3.22564734338937 | 2.70560408215726 | 1.83366683613179 | 2.59878045361619 | 1.72115392846131 | 2.70760748886621 | 2.94781351424673 | 3.11456569516937 | 1.97899832393598 |
| cis-5,8,11,14,17-Eicosapentaenoic acid | 2.45002325866031 | 2.56875382352416 | 2.00844663879707 | 6.67319673773013 | 4.81652201721732 | 4.85608660720745 | 1.19507399171332 | 1.16770053604917 | 2.43551955854488 | 4.03635326525406 | 3.62037044318046 | 2.37685310554436 | 5.03537178846602 | 5.01703833972005 | 4.02394810001788 |
| cis-4,7,10,13,16,19-Docosahexaenoic acid | 0.00719517245841716 | 0.140221663573392 | 0.153134697431088 | 0.0306249241370212 | 0.0278030205210916 | 0.219720681547809 | 0.00335201742299044 | 0.0208549796671308 | 0.0079186198392641 | 0.873551688576241 | 0.290182153388092 | 0.23354594638132 | 0.154045097254624 | 0.0370780742808146 | 0.0636562052370566 |
| Bombykol | 24.1587986763297 | 27.1787719634177 | 30.1376279260786 | 111.177883427972 | 94.5628645694756 | 54.1704953806151 | 10.7044857486427 | 11.6309927452174 | 12.5851165013731 | 284.904920263628 | 58.9427793238867 | 48.4724911945209 | 1065.5791139584 | 125.896720710234 | 49.3293005509145 |
| Azelaic acid | 153.859906154853 | 122.730999066816 | 140.641963067073 | 70.8808370220702 | 54.1986472158942 | 88.9944791474861 | 111.161254843146 | 91.9545701275259 | 97.6335085519419 | 41.31110596 | 47.2052923315257 | 46.0415034262565 | 57.7615642327253 | 48.553331458498 | 65.1920584473651 |
| Arachidonic acid | 0.35602179184077 | 0.503545651395459 | 0.376310331403334 | 1.71807588497836 | 1.81901301187646 | 1.82139418492059 | 0.176479809792311 | 0.0866950794345984 | 0.379880573670279 | 0.655613107983861 | 0.221995055337878 | 0.53412552770368 | 1.37758831577752 | 1.44979345101345 | 1.22216059219855 |
| Adipic acid | 0.651590133170856 | 0.655809515494889 | 0.642718085120819 | 1.67859855907228 | 1.18879903693422 | 1.95812968799993 | 0.436233823685006 | 0.585870512385375 | 0.661436613685982 | 0.821056952383088 | 0.778115925089035 | 0.869280870898688 | 2.11156919565635 | 0.857244162385373 | 1.74233896200186 |
| 10-Hydroxydecanoate | 0.490031946710863 | 0.370132535504103 | 0.644846604139524 | 13.1208488497942 | 6.1215709747833 | 9.3187315882705 | 0.617554624658967 | 0.508257835997149 | 0.260193672335782 | 2.12320197391135 | 0.28093193475255 | 0.306504962706256 | 8.56264123215365 | 8.58434567262508 | 2.63749291402203 |
| (+/-)-2-Propyl-4-pentenoic acid | 0.330668929529963 | 0.301803119875016 | 0.250327832435537 | 1.09559981973594 | 0.885358526900822 | 1.14176179343989 | 0.0254238670130996 | 0.164593198276143 | 0.199880702004181 | 0.0343295165857603 | 0.162878032597397 | 0.369680399273938 | 0.122302019426715 | 0.0964775556844205 | 0.0744578384854246 |
| Octadecanoic acid | 1.41865650350302 | 2.23394806080484 | 4.30639035651538 | 5.31427322140424 | 12.4503893926481 | 9.90801126635579 | 0.698354775187356 | 1.29011041922445 | 2.0350398428421 | 3.31651049154703 | 2.34227531411974 | 8.80957020355559 | 6.23184575401399 | 4.72731894715333 | 2.33893861993641 |
| Heptadecatrienal | 2.27504669121309 | 1.80138907176432 | 1.84343950542358 | 4.48220204720033 | 5.67763700491901 | 4.35121868181067 | 0.884972444689883 | 1.07415462848713 | 1.93981482665151 | 0.974702906272793 | 1.46958861987578 | 0.919553073144681 | 1.36147610111084 | 1.47039046036168 | 1.80106865931109 |
| Estriol | 4.55872722303393 | 6.34569544256335 | 5.70000992577351 | 3.04798031153101 | 2.91723568470739 | 2.78859359934609 | 2.07069351624514 | 2.34967000120248 | 4.94545755443028 | 4.30048180259257 | 4.2542675042205 | 3.27895463158511 | 33.1664960926885 | 27.7381578537198 | 29.2853410902904 |
| Estrane | 0.546699695224053 | 0.457063588992849 | 1.91567632155101 | 4.85890092417417 | 4.33114236360208 | 1.26427456630016 | 0.854518780407991 | 0.777476078963455 | 0.0419893697994311 | 25.0393255776183 | 4.43147290149658 | 2.48288518889118 | 82.5059124590608 | 10.6723420574464 | 2.08083798542706 |
| Dodecanamide | 40.0501734053546 | 44.8971284807452 | 39.89903683 | 105.69232559619 | 90.1076176959425 | 128.325352958821 | 17.2730296577656 | 21.0624534635003 | 28.6793722041831 | 40.3260912218592 | 22.4650892002487 | 39.9151267395559 | 39.0908694595992 | 44.4535066395066 | 57.3408244287838 |
| Dihydromonacolin L acid | 6.79651527909694 | 4.64225183117513 | 6.76768558070798 | 17.9403422809761 | 23.9604948762468 | 18.1932228567799 | 5.14647339403537 | 3.78119050113406 | 7.11150324443744 | 6.07523922613568 | 4.50855039464553 | 6.75645494237589 | 5.5739773338804 | 5.31957559687518 | 6.94321845794657 |
| Dibutyl adipate | 10.299582944576 | 9.84375331061321 | 10.0677873954243 | 11.2647301820546 | 11.4896526130969 | 9.02124915334975 | 57.4255823696125 | 48.3913536707925 | 168.965118029777 | 3.63752186508415 | 4.96750697960554 | 5.68117415558159 | 3.52105629070826 | 5.36540425475953 | 3.71922638711101 |
| alpha-Isopropylmalate | 34.1777020732559 | 45.4749189528794 | 31.4210118521237 | 91.5581332746499 | 99.9184341965554 | 91.4749529174773 | 18.4661413432351 | 21.2465687690526 | 26.889866526903 | 38.4493033106753 | 36.4109602106145 | 47.3661253100852 | 50.9440545755013 | 53.6990609308045 | 44.9305419788954 |
| 9-Oxononanoic acid | 1.51910960112745 | 1.78969808559122 | 2.29791051980354 | 3.02042795314539 | 3.21457416124909 | 3.61778741686969 | 2.6293260460044 | 2.32518121598352 | 2.78101892704103 | 2.04420812148821 | 1.16105508935668 | 1.70354370124425 | 1.93087111408417 | 3.17504117407759 | 2.51233921897891 |
| 9,10-Epoxy-13-hydroxy-11-octadecenoate | 6.11180025508804 | 5.9474094834433 | 7.40489024051717 | 5.34263874864578 | 6.1969646507119 | 4.48495081833785 | 23.2105857864468 | 13.3689590422258 | 70.6085028675418 | 2.07739994418848 | 2.79393293853201 | 2.31621877409342 | 2.70152722624371 | 3.10463952157828 | 7.53104277129866 |
| 9,10-Dihydroxystearate | 13.0375862465281 | 17.1706025410483 | 16.1818337385079 | 5.11557720065602 | 11.3055664054526 | 3.64852585810571 | 25.9688094462345 | 24.5703412612773 | 92.7022480018707 | 5.62050989646595 | 7.59524045583362 | 8.56215560319073 | 11.1847553910579 | 13.7672308282393 | 29.0297753489057 |

**Table S3** *Cont.*

| Compound name | A1_1 | A1_2 | A1_3 | B3_1 | B3_2 | B3_3 | C2_1 | C2_2 | C2_3 | C3_1 | C3_2 | C3_3 | M0_1 | M0_2 | M0_3 |
| --- | --- | --- | --- | --- | --- | --- | --- | --- | --- | --- | --- | --- | --- | --- | --- |
| 9,10-DiHOME | 13.6406287332238 | 22.8381017992177 | 27.1905044254062 | 72.0665043417292 | 87.0543581309931 | 73.0497731643569 | 15.6278902281794 | 8.5750198753197 | 23.9785132388599 | 21.9272654540997 | 8.24271728147655 | 15.2418833946063 | 10.4758561430176 | 11.9027986058236 | 12.3616695345753 |
| 9,10,13-TriHOME | 2.77574326333119 | 2.15935493096007 | 2.75918223721309 | 0.210193080942184 | 0.65760882029521 | 0.0560513637437724 | 23.4625196975824 | 18.386009084711 | 72.8252660550783 | 1.71631754847841 | 2.37500376258288 | 2.32275812283898 | 3.56706154023255 | 3.30649643233551 | 8.82095595422938 |
| 3-Deoxyestradiol | 0.37481649918681 | 0.479381116789521 | 0.348979983968121 | 1.13407418023558 | 0.701493697498777 | 0.675801936552072 | 0.198884042057498 | 0.182653924407475 | 0.233307572069386 | 0.157422475759194 | 0.172493503687467 | 0.216138868914706 | 0.234139381519916 | 0.314027980028182 | 0.33477942003935 |
| 12,13-Epoxy-9-hydroxy-10-octadecenoate | 10.1485247751411 | 10.7047773906717 | 7.26981167084568 | 12.7771065497805 | 18.9446055558963 | 12.8908750372901 | 42.5908373522367 | 29.4933984274747 | 106.212820680505 | 8.62347862560008 | 7.30963323990951 | 6.61589752270668 | 11.4754457357787 | 6.29384216673408 | 19.133567646503 |
| 12-(2,3-Dihydroxycyclopentyl)-2-dodecanone | 1.18582462695477 | 1.1562183256414 | 0.8983982923242 | 25.3632493913206 | 36.4995467990628 | 28.3987586010795 | 3.0151947810177 | 1.68935648944219 | 2.49919551240339 | 3.69913740141775 | 3.4242566554529 | 3.27801326406749 | 3.71524008283314 | 2.49595237 | 3.48904649516007 |
| 11-Aminoundecanoic acid | 3.22920053662366 | 3.44547597973299 | 3.01220528403798 | 3.56513923655778 | 3.70139487054791 | 2.95805536643826 | 3.47415307401913 | 2.81833254087719 | 1.85630586613113 | 2.5695357953199 | 3.24073975666993 | 2.83186395609392 | 3.24297726106826 | 3.90254247293012 | 3.80152210233402 |
| 1,4-Bis(2-ethylhexyl) sulfosuccinate | 14.001551782662 | 23.7868607412023 | 23.0128509956529 | 1.40860936084146 | 2.0868674238928 | 1.56066177120209 | 4.14908772079349 | 15.3559911919945 | 10.2480665005079 | 5.20911092285959 | 8.44035373144185 | 10.1873832909609 | 3.09926844720771 | 3.03414426718314 | 2.69096945466019 |
| Epiandrosterone | 0.12418487783837 | 0.203058522995834 | 0.496820190846869 | 4.08049122897104 | 3.73744629876548 | 4.55294121018633 | 0.380351999544878 | 0.250856625402254 | 0.290948354547074 | 1.14539547733542 | 0.496095297837486 | 0.687058629440741 | 1.56729332509712 | 1.84457683607326 | 0.995394224421216 |
| Biotin sulfone | 0.060383096138122 | 0.0453110624688307 | 0.052787508556571 | 0.3367172660895 | 0.382706800852338 | 0.362164332301378 | 0.694525714439061 | 0.417424814663691 | 1.57616048628226 | 0.130952473258402 | 0.0454513600586711 | 0.166933928131614 | 0.104083971169261 | 0.146521558624076 | 0.0243384839700209 |
| Adrenosterone | 0.598825396760262 | 0.683689974687717 | 0.551108606357123 | 0.248624447004424 | 0.0573473430882456 | 0.0670284513512306 | 0.483249252952689 | 0.314000327140682 | 0.821644156667858 | 0.227929742330345 | 0.209166640659266 | 0.0222625939595564 | 1.34283296540965 | 1.60527591076343 | 1.13182153559771 |
| 21-Hydroxypregnenolone | 3.77829755420207 | 2.21629808404495 | 1.93192644255123 | 1.06954153449763 | 1.42000124805934 | 1.54414911967902 | 2.25411344866421 | 1.45895348702042 | 1.32001845418076 | 1.03641841894337 | 0.962352706588476 | 1.34367555767243 | 1.03084779864376 | 2.83206522003343 | 1.22596309488674 |
| 11beta-Hydroxyprogesterone | 6.5211623403567 | 4.40593502611208 | 4.1556699135128 | 0.226962646743208 | 0.410562878059999 | 0.41614460608602 | 5.54462593521792 | 3.72010123386531 | 3.0282459094468 | 0.423431640431372 | 0.550969142408974 | 0.790004981274712 | 0.415369337 | 1.15129032486017 | 0.511097991475671 |
| 11beta-Hydroxyandrosterone | 5.41211639327046 | 6.46182379505105 | 5.58216490164575 | 26.055038468595 | 25.3012176360807 | 18.3935091880164 | 7.58823802974332 | 4.98592646941611 | 5.14583003479333 | 6.30891865047716 | 6.88526827564306 | 11.9476889815793 | 8.25422458086957 | 14.4425568784981 | 17.9214802325567 |
| SM(d18:0/16:1(9Z)) | 6.40005306445709 | 5.38981813895464 | 3.88251712480291 | 26.6943973291913 | 14.8270457023894 | 19.7635252114959 | 4.15431581763894 | 7.34438866700207 | 3.38137475091506 | 9.61027700284564 | 6.11554288496633 | 9.39449986841874 | 10.4126569233849 | 9.13850336103071 | 7.60365544950246 |
| Pumiliotoxin 251D | 1.64127449846742 | 2.85568273728163 | 3.58922581066988 | 3.15555179753864 | 6.96521409857715 | 5.79561254380097 | 0.769746884279841 | 1.21404926097127 | 4.05319594679562 | 4.63202737240139 | 2.22602149127403 | 3.37595034372188 | 9.42358293575326 | 4.18759477663772 | 1.6831145270799 |
| Decarbamoylsaxitoxin | 74.4991907516193 | 43.8288314184283 | 56.5930819455638 | 5.3833041129666 | 2.37827766267539 | 5.15176519666897 | 4.66955519486167 | 3.68028798026927 | 3.16573500593892 | 2.88841861365059 | 2.39420375440429 | 2.44173672071164 | 7.07088970941816 | 2.38070292353255 | 3.34151354314223 |
| Tributyrin | 26.5674655980282 | 10.5658986156138 | 15.3422197698691 | 2.00218004794335 | 3.58061533836697 | 4.33342763752286 | 9.47648397653033 | 9.22633473192089 | 6.99289265448045 | 4.35928939809393 | 6.12082607626495 | 3.57958746324479 | 4.23683093623386 | 10.1344715990234 | 5.17924538571859 |
| L-Malate | 4.28831713421085 | 4.30791169691426 | 8.26617115625867 | 0.0762260999683927 | 0.289125282747595 | 0.0813174486963461 | 1.99491936337445 | 3.74408107311779 | 2.73325264697432 | 39.0844443302519 | 755.759593396605 | 32.7629624105631 | 0.144440051473196 | 0.031461527812564 | 0.433051203898284 |
| D-alpha-Hydroxyglutaric acid | 0.427487722829397 | 0.636831064282951 | 0.389703426202134 | 0.819324132117772 | 0.857010243688875 | 0.297134185908323 | 0.215387427159339 | 0.122855861251063 | 0.347158832383039 | 0.416289227882058 | 0.429864801593527 | 0.731366567448489 | 1.16916939280272 | 0.665745176694352 | 0.526379506724017 |
| Oxindole | 2.62598024749498 | 2.74938586216499 | 2.11140921658467 | 0.237128209099016 | 10.6473493891867 | 18.670366210612 | 1.73602825988339 | 1.02673700149113 | 1.81069322842577 | 0.097175305036142 | 1.15112416962315 | 2.71473611350423 | 78.1448717908528 | 61.0884378041807 | 109.59221033794 |
| N-Acetylserotonin | 0.10984362079085 | 0.0440352616956396 | 0.106425646716368 | 0.166467523763341 | 0.251123935192564 | 0.168315763581267 | 0.0328221012633519 | 0.0147365496777566 | 0.026610734268693 | 0.0685911647014889 | 0.0907444105813347 | 0.0627232967213477 | 0.098922013790641 | 0.167302637326288 | 0.0661492325627964 |
| L-Tryptophan | 15.3333768210574 | 5.88674083810503 | 11.2363445913428 | 6.38693053268794 | 6.59108839331337 | 8.28114485262139 | 15.6372985979621 | 30.2070595060163 | 27.9333670754366 | 17.3973511108995 | 29.2031135917476 | 38.1348101440122 | 6.61413408960638 | 8.94136544365502 | 10.0998041165563 |
| Indole-3-pyruvic acid | 0.0269849914966955 | 0.0543434640112668 | 0.0712742779615702 | 0.332657736747035 | 0.230507666162564 | 0.467669443738995 | 0.0317924733989335 | 0.172100749423538 | 0.0527091518914641 | 0.132716233648947 | 0.0797570569187685 | 0.0416285161514302 | 0.251927822824704 | 0.293658555890325 | 0.0294160246567439 |

**Table S3** *Cont.*

| Compound name | A1_1 | A1_2 | A1_3 | B3_1 | B3_2 | B3_3 | C2_1 | C2_2 | C2_3 | C3_1 | C3_2 | C3_3 | M0_1 | M0_2 | M0_3 |
| --- | --- | --- | --- | --- | --- | --- | --- | --- | --- | --- | --- | --- | --- | --- | --- |
| Indole-3-carboxaldehyde | 2.59395719640559 | 2.75196980984444 | 3.19814524268703 | 63.4541663496831 | 73.419472410155 | 77.102291237815 | 16.8129609319864 | 9.65456552410516 | 13.5717432862553 | 12.1224652423657 | 10.1968366536132 | 16.2645338065739 | 27.383380331672 | 25.8270823931078 | 24.6909825192413 |
| Indole-3-butyric acid | 0.116264039541412 | 0.119872400811791 | 0.115887761642293 | 0.0534240301047485 | 0.105863636808035 | 0.0949024890177163 | 0.0550274028454477 | 0.0376023771383097 | 0.0328170877513692 | 0.0554982199969865 | 0.096296395810765 | 0.113625820078051 | 0.0258283875846731 | 0.0292512671801612 | 0.0605982226358873 |
| Indole | 0.209117120186224 | 0.317041308831829 | 0.249826623331448 | 7.511439156 | 5.1922966271487 | 8.14192142905158 | 0.582303512901428 | 0.144467710901582 | 0.137467639585289 | 1.98127368097637 | 0.166592737681119 | 0.597184391213767 | 4.09116585795408 | 3.71930686641673 | 2.49353987598906 |
| 5-Methoxyindoleacetate | 0.161532342118942 | 0.181889549001651 | 0.160468757886513 | 0.153897951063197 | 0.314119907328086 | 0.315939735138138 | 0.242198704248423 | 0.269839492912845 | 0.218482887079527 | 0.361112762767876 | 0.247818159990362 | 0.732244362078017 | 0.462251584573052 | 0.302089432349819 | 0.590522075142939 |
| Norharmane | 2.01574782458618 | 3.3004135589966 | 2.30354647872591 | 4.1603135141924 | 4.67395182576329 | 4.80709031628072 | 0.59921389596546 | 1.24085898999717 | 1.74251540468059 | 1.26580797287945 | 2.25962432896443 | 3.83020184567705 | 2.5586181080608 | 3.61506543974533 | 2.8912992031821 |
| N,N-Dihydroxy-L-tryptophan | 0.530496622388591 | 0.271665559281622 | 0.699140748000364 | 2.04916888466691 | 2.69347454242033 | 2.37253896803122 | 2.54399695488285 | 2.04281336785396 | 2.18232445051152 | 1.11938850108532 | 3.81092134597686 | 5.76352805170752 | 2.67733311069254 | 2.89894114217743 | 3.10751010365318 |
| Slaframine | 0.911634301897881 | 1.13611206535526 | 0.862048977163862 | 3.76153618019398 | 1.38039766622786 | 3.88784096922384 | 1.16022871533055 | 0.636154503307744 | 0.705108249202111 | 1.43918293422801 | 0.666684554343149 | 0.568018300446127 | 5.65022268353681 | 5.82090512912242 | 5.63404739909696 |
| isoferulic acid | 9.61105613717473 | 12.8068971078271 | 8.81082586607431 | 57.5442047301129 | 58.7058646712388 | 56.8294421865867 | 7.93118997440795 | 6.24391831020096 | 7.08726392205097 | 18.9288782020003 | 11.1769989033011 | 19.9354661982941 | 23.5551993370219 | 24.124570449865 | 19.3053280855775 |
| Elaeokanine C | 11.0128434472732 | 10.0040685700829 | 9.63663637649028 | 3.10891864376672 | 2.67361986477723 | 2.55563936776728 | 5.32146226189708 | 6.51772925073053 | 4.40164834283231 | 8.37872462749835 | 12.2827418426537 | 12.2733107250117 | 12.132692079972 | 11.4767102990493 | 20.3422068889886 |
| Coumarin | 0.585573599687129 | 0.746091191208396 | 0.846579423589715 | 0.716953539425301 | 0.780623468820992 | 0.933754488100696 | 0.570788846738589 | 0.503566926453295 | 0.721555069250522 | 0.653304218218019 | 0.709963747501947 | 0.70347679979837 | 1.39813849067412 | 1.28903525039403 | 0.996107212195198 |
| N-Dimethyl-2-aminoethylphosphonate | 0.0437523305071524 | 0.0794575155258566 | 0.043212944189123 | 0.444179528235995 | 0.500347524451286 | 0.317529776843506 | 0.00619998800877479 | 0.00621947724004262 | 0.0174925656955538 | 0.135504037944123 | 0.0258410460081434 | 0.0157312089784625 | 0.233847807486218 | 0.138337830259501 | 0.0900318432845686 |
| L-CARNITINE | 2.09199944134372 | 2.59446599028783 | 2.29185077521659 | 0.35533449930856 | 0.557200839738447 | 0.327173977435591 | 1.21187469789724 | 1.84104427070912 | 1.36924498816698 | 1.26645168854185 | 2.18797307773655 | 1.40338194751822 | 3.55236166417184 | 4.84821222177457 | 3.45354110484294 |
| 4-Methylthiobutanaldoxime | 9.95092872546974 | 6.94779761705079 | 7.82147583238408 | 4.39796602856128 | 4.41883650614353 | 5.08885064674098 | 2.49210662968095 | 4.54179704240836 | 1.4228172101643 | 5.79784184977459 | 19.9114677884778 | 5.00567167766994 | 5.89536012855752 | 8.36978845940296 | 3.07060778220248 |
| 2-epi-5-epi-Valiolone 7-phosphate | 3.27678172885044 | 3.39612999363427 | 4.8430908010327 | 0.0800840934173209 | 0.0373878067628801 | 0.811229918277385 | 4.68616517533593 | 4.23038665334646 | 13.0718351698766 | 0.0418201018138867 | 0.450782763515682 | 0.22055685875103 | 0.291777880497824 | 0.385333116880522 | 0.448531605467552 |
| trans-urocanate | 207.549807497007 | 201.467691395259 | 215.42736990776 | 38.046544021852 | 58.2203480613506 | 26.9706815832585 | 186.878971463534 | 214.20906527884 | 181.506466699268 | 67.475720045365 | 94.8945179165665 | 132.56664289939 | 42.0205458863907 | 49.4115112868228 | 60.739133532343 |
| Terephthalic acid | 30.1433758162884 | 31.4785115582001 | 33.9719224442209 | 31.6433430264914 | 27.3252511823532 | 31.448003948367 | 21.1999077900352 | 23.654752564876 | 23.6993877915168 | 17.7948784192714 | 13.7727013144514 | 25.5175724233554 | 22.4250034791721 | 25.2180598995628 | 27.0524327424884 |
| T-2 Toxin | 0.135769899814982 | 0.21125888992852 | 0.286042888728555 | 0.204296216447807 | 0.0390429018814711 | 0.090541616624748 | 0.130380668810761 | 0.0588015638708517 | 0.150062341433596 | 0.0623121017747183 | 0.0940194939150094 | 0.03549814989834 | 0.178972362749564 | 0.118779405423867 | 0.100159162981691 |
| Stearamide | 9.89224775139816 | 12.8037082932953 | 10.3261151070284 | 44.3618022858167 | 28.6604357274108 | 25.9430048531117 | 5.45218224944302 | 6.31050123590931 | 6.46363942311214 | 35.0953502712694 | 17.8295293358401 | 22.0301777882717 | 264.366608881232 | 49.64580495 | 25.1956726854562 |
| p-Octopamine | 1.26526955082495 | 1.59129968138245 | 0.943334446804802 | 2.5763165505092 | 2.84016716860879 | 2.70138852946205 | 0.55425861129579 | 0.929964881372531 | 1.15225606360254 | 1.57548253329335 | 1.32900864936001 | 1.78419887041487 | 1.96223300967834 | 1.6542050845407 | 1.34405304139316 |
| Picolinic acid | 60.1878398476197 | 52.1925827368688 | 46.1892912865228 | 56.3685502910346 | 63.5022628926092 | 145.965908227187 | 22.4576093807111 | 12.5145730129935 | 19.0573860423911 | 25.9497526718192 | 44.6384734401655 | 25.4728801938394 | 71.6442899862428 | 44.7761339537646 | 131.915805195612 |
| Phenylethylamine | 3.35513357240888 | 3.52511732965137 | 2.86600488024215 | 15.1361775827927 | 15.5405943780242 | 16.980042733835 | 7.97082401556786 | 7.20667536377338 | 8.26077287345684 | 11.3035079085334 | 17.7340300118983 | 13.9701773264894 | 11.9520006778523 | 12.0196528347465 | 10.3533882409402 |
| Palmitoylethanolamide | 2.22298515836565 | 3.08694651777351 | 2.32351355986169 | 5.15451055458818 | 6.14430249793494 | 5.15752282239575 | 0.828073595796335 | 0.917126639109398 | 1.92833797254971 | 1.36013042162732 | 1.80890444050134 | 2.22472727029113 | 2.56952435419048 | 3.07446938693262 | 2.24339099321563 |
| Nudifloramide | 0.11921805732155 | 0.0453187237862386 | 0.0198747436868275 | 1.04655815381447 | 1.77520751636176 | 1.3856963854092 | 0.423139073095662 | 0.232624413351878 | 0.240927361901073 | 0.125329921096432 | 0.212822196523308 | 0.13050838095238 | 0.208777822665184 | 0.147080196695288 | 0.152989021839247 |

**Table S3** *Cont.*

| Compound name | A1_1 | A1_2 | A1_3 | B3_1 | B3_2 | B3_3 | C2_1 | C2_2 | C2_3 | C3_1 | C3_2 | C3_3 | M0_1 | M0_2 | M0_3 |
| --- | --- | --- | --- | --- | --- | --- | --- | --- | --- | --- | --- | --- | --- | --- | --- |
| Nicotinamide | 17.1944430707886 | 19.5922149565209 | 15.3577797662159 | 95.9002980593557 | 94.2800003204714 | 92.0367802076359 | 9.70710240748942 | 8.91577161539212 | 10.3911195678705 | 37.7741179857805 | 26.4427991833408 | 30.648130693135 | 82.8439433711734 | 76.2373234585298 | 51.1435080076646 |
| Methyl p-tert-butylphenylacetate | 0.132073789130512 | 0.280001049985789 | 0.0675587220938954 | 0.098958619525321 | 0.0950100091222584 | 0.160084578889397 | 0.158080368325007 | 0.0742921088651608 | 0.027193414552442 | 0.289342181766852 | 0.490521222313045 | 0.125774765752831 | 2.35055216254265 | 3.1817396354928 | 2.96470582520183 |
| Linoleic acid | 1.9546007221776 | 2.19684396218983 | 1.70169583391483 | 1.56110752996104 | 2.23128355075316 | 1.81957218171756 | 1.50120227717042 | 2.39344504423731 | 7.64561342960376 | 0.880710116121732 | 1.07948731495314 | 1.18058276955306 | 1.14551706376707 | 1.12728957793438 | 1.86845573016364 |
| Kaurenoic acid | 3.62804581591747 | 3.69772108145038 | 2.61352281091872 | 1.67845332240188 | 3.7311308511712 | 2.56417938634264 | 3.22974410375738 | 4.54463852256665 | 3.13648385101815 | 4.98429422029108 | 3.33765276909475 | 16.1807020717379 | 2.70232729640784 | 1.56687008583306 | 1.94789524260532 |
| Isopimaric acid | 0.330309163601237 | 0.126451631527289 | 0.141732442520634 | 0.280313433148471 | 0.019664160316859 | 0.0262869357701043 | 0.0938750816391167 | 0.0952618748856197 | 0.161456733665246 | 0.0638132602261777 | 0.0766112707612667 | 1.15117744776469 | 0.059258049886842 | 0.310955354287132 | 0.103506891383122 |
| Geraniol | 5.32471482644586 | 4.62319171662886 | 4.67252429820409 | 6.6019126119791 | 5.09081324749734 | 5.13308576140772 | 4.06030076222227 | 3.58477783376421 | 2.87315928460792 | 3.80419326055859 | 7.40840938003434 | 2.81222063643499 | 3.85958232129341 | 3.87239521182586 | 5.23457042818821 |
| Farnesol | 0.315392888773294 | 0.386643781116891 | 0.313692353602013 | 1.0164924083278 | 1.04157410532273 | 1.10838173552152 | 0.193646294746392 | 0.208509093088137 | 0.272368669162377 | 0.436908100849998 | 0.395796335620876 | 0.58269847096129 | 0.643691501725898 | 0.226733103558263 | 0.443053066172096 |
| D-Glucurono-6,3-lactone | 0.0737414197782919 | 0.0886479841671775 | 0.102048668014249 | 0.010642068650656 | 0.116210164262418 | 0.00728680591826238 | 0.093123095532427 | 0.121355053058613 | 0.0960427867219119 | 0.296722788508618 | 0.469445812090212 | 0.234654626335363 | 0.111955655345977 | 0.130499347619865 | 0.22327197264125 |
| D-(+)-Phenyllactic acid | 10.8586422453811 | 7.78434227155698 | 8.7770078017961 | 10.2018351481688 | 8.12077560955141 | 10.882166602194 | 4.76064679525457 | 7.99859991306168 | 4.0729315746152 | 16.2268782348836 | 18.6952214363564 | 27.8591269998525 | 11.3785394787988 | 10.9598462780643 | 22.3027417786003 |
| Beta-Zearalanol | 2.78605091598817 | 2.95156090869633 | 3.04046954150183 | 1.68480517975877 | 1.46668263150143 | 1.59544333066387 | 1.65436802205652 | 1.23979620836855 | 1.99757493710781 | 2.66956152979878 | 3.66789127550504 | 1.63181107141146 | 6.68896635604617 | 6.99125315266887 | 6.13290118851327 |
| beta-D-Glucopyranosyl abscisate | 0.603635361262739 | 0.588739442426462 | 0.599075428335946 | 0.471940591637002 | 0.21421877954773 | 0.236953719095579 | 0.273707885252624 | 0.10408348662176 | 0.177083950307196 | 2.06162112877509 | 22.9658276248636 | 0.839083904565214 | 0.658569294236434 | 0.571499610665472 | 0.611829526879061 |
| Benzamide | 2.57474569105286 | 2.11398817656049 | 2.23905161689023 | 4.29899908062106 | 2.90102478996575 | 4.85473934317736 | 1.13456707943285 | 0.96776028024944 | 1.17495615960158 | 2.15817549339275 | 1.12666432909327 | 1.11411243497694 | 4.57210994326957 | 4.07934769994101 | 4.22518304669951 |
| 4-Hydroxyphenylpyruvic acid | 0.133327552186549 | 0.182744737174121 | 0.107934894349995 | 0.512479610088144 | 0.459286004143268 | 0.572388629068467 | 0.00281410458739952 | 0.0448371506631704 | 0.0671341579574518 | 0.101739244425998 | 0.03660473175505 | 0.0448233068666891 | 0.300620041954201 | 0.334611241162925 | 0.345655747537823 |
| 4-Hydroxybenzaldehyde | 17.0830027231125 | 21.0509845875622 | 20.5304498473546 | 55.9131853549486 | 103.342808954733 | 49.4646836431733 | 28.5752741640654 | 23.2589126932845 | 24.2585410209616 | 36.8911363341515 | 20.4496510943118 | 17.9526609698901 | 32.6895145379473 | 35.1835172512567 | 61.3909005453289 |
| 4-Ethylphenol | 32.6051661281816 | 30.4186720409875 | 31.9419429746173 | 45.2792501085785 | 40.3678688629255 | 41.7725416653724 | 24.7111922300745 | 0.0900963965382872 | 30.9543652965986 | 11.7650725352193 | 34.5493193991085 | 19.5697022592081 | 34.8670181332338 | 43.6813943607738 | 35.5398002886696 |
| 4-(1,2-Dihydroxyethyl)benzene-1,2-diol | 0.0938463705742415 | 0.369029061848028 | 0.192661702739963 | 0.034439164356616 | 0.0590415731965146 | 0.137781271733604 | 0.0358886619310689 | 0.0886227296580948 | 0.0576144008156357 | 0.222003517810828 | 0.164553038962704 | 0.155963945328889 | 0.0667631340806218 | 0.199787447984464 | 0.0503190965785784 |
| 3-Hydroxybenzoic acid | 107.162446532101 | 101.563909635061 | 232.827720357399 | 712.665120354725 | 645.116914017575 | 668.888100569227 | 69.8840195686501 | 57.7482974057165 | 53.5042625661629 | 245.225982858496 | 124.007257650427 | 173.940130629749 | 421.477363120749 | 370.417357379621 | 266.300376680676 |
| 2-Phenylacetamide | 9.48517466165974 | 12.0559590656278 | 8.47606891681577 | 44.7722098366999 | 44.3666777549891 | 50.6438556618866 | 38.2122775883236 | 33.7470932314868 | 33.976911152557 | 24.6127210019995 | 19.1632570620362 | 19.8215130563389 | 17.3712062763727 | 16.2407288774479 | 14.7729798480896 |
| 2-Hydroxymethyl benzoic acid | 14.4286803488216 | 11.6142446904005 | 12.2965557012894 | 17.1471116640188 | 17.5367334008445 | 16.1954941951179 | 8.76558381575294 | 12.6712762483655 | 13.3393850320429 | 6.58984176171728 | 8.86482516065507 | 7.67289580903169 | 8.47943510471104 | 11.2436077688263 | 7.98681986546915 |
| Victoxinine | 0.210780178044202 | 0.21634636765104 | 0.145946880717681 | 2.13256124532083 | 1.94084329162358 | 2.16017349287748 | 0.165575712483754 | 0.08281404423043 | 0.0300723665008271 | 0.89770786544865 | 0.240509031383458 | 0.383814614915855 | 1.38097536448474 | 1.56095647230326 | 0.852156399899849 |
| Tetrahydrocytisine | 0.141193876092494 | 0.176541904817569 | 0.169993093164298 | 2.46502078252213 | 1.24055670804073 | 2.67884791335665 | 0.0566690198349368 | 0.0792373691772187 | 0.0296054832307976 | 0.264886131148357 | 0.0586519772387339 | 0.0673845150438943 | 0.885526913663294 | 1.10482466471663 | 0.476522110178825 |
| Styrene | 10.9681185564476 | 8.2213462451529 | 7.58072499700879 | 31.6239478831778 | 34.6851123531707 | 36.0264109999633 | 19.2846319495524 | 18.5426495780934 | 20.9537093537695 | 28.2433878890639 | 48.4352887689145 | 35.7091638940752 | 31.6317626856858 | 31.9472193777567 | 27.6044192030041 |
| Strongylophorin 22 | 0.264686034239243 | 0.571579251931873 | 0.4074806189757 | 2.32915368291912 | 2.49127544873129 | 2.61543015631991 | 0.226432886724828 | 0.267096888387918 | 0.305339936235429 | 0.600285469101803 | 0.499216979622837 | 0.84071790874165 | 0.899020348840891 | 0.694146425997373 | 0.700813030777942 |

**Table S3** *Cont.*

| Compound name | A1_1 | A1_2 | A1_3 | B3_1 | B3_2 | B3_3 | C2_1 | C2_2 | C2_3 | C3_1 | C3_2 | C3_3 | M0_1 | M0_2 | M0_3 |
| --- | --- | --- | --- | --- | --- | --- | --- | --- | --- | --- | --- | --- | --- | --- | --- |
| Sapropterin | 0.554702411615248 | 0.256187734441055 | 0.46486674505134 | 0.375834868444884 | 0.328311384585447 | 0.507005881888431 | 0.0183012778153393 | 0.0846253423375095 | 0.00458962585951629 | 0.199100582195123 | 0.947905812107251 | 0.361661178452471 | 0.307971250848462 | 0.373769763749737 | 0.366806189838548 |
| Quinolactacin A | 0.104561705945942 | 0.373959813189534 | 0.0576422751278157 | 0.399813947952769 | 0.322036527564165 | 0.327118696050604 | 0.165188077510156 | 0.165684008387477 | 0.145892133183658 | 0.485976924780102 | 0.552478400325818 | 0.394663706948042 | 5.0513578332879 | 4.56916815993151 | 4.2935974752008 |
| P-Toluenesulfonic acid | 259.604925231273 | 63.8310145554353 | 199.972518122666 | 795.757570262484 | 679.422684190656 | 495.240799974727 | 155.295412098743 | 167.58571338962 | 89.8019725955743 | 750.501438977124 | 1254.18528026894 | 1762.92090121965 | 41853.4885105099 | 88209.6229587623 | 37328.6613968016 |
| Plakortic acid | 0.396109157849518 | 0.303692472689506 | 0.264016060783532 | 0.170791934399454 | 0.53565039373559 | 0.27775618827432 | 5.74649923900358 | 2.87489145560818 | 4.25808296804138 | 0.0217946794921813 | 0.0137313878140583 | 0.0153068189384363 | 0.270393036124475 | 0.857516240260964 | 0.52575892340526 |
| Pandangolide 1 | 17.2509467537927 | 13.3416439665487 | 17.0854018150948 | 6.56531287206083 | 5.51262454270053 | 6.8786735779507 | 14.9124201472081 | 20.6352453395113 | 33.89549407 | 4.83141105249848 | 6.98160325464347 | 5.70465248385179 | 6.89044861103236 | 6.59167124485124 | 8.61825971832911 |
| nigerapyrone A | 1.53503188017987 | 0.555925621029807 | 0.800688059434941 | 0.0338093610246335 | 0.0188945158370373 | 0.0215500252523879 | 0.663614801632127 | 2.72489461383423 | 0.334035286770018 | 7.17128591121705 | 9.64362867528712 | 1.44460549611899 | 0.142785152015325 | 0.254079087875924 | 0.0445364257832096 |
| Neopterin | 25.174514066338 | 20.5148865212307 | 23.4635364596356 | 4.23075549513757 | 3.53665044764325 | 3.75557381993532 | 5.15998274090373 | 10.9624965343499 | 5.26880279056612 | 10.7985473730956 | 12.9700213320765 | 8.82230316489989 | 2.36730403575526 | 3.02154469883778 | 2.62076948789959 |
| Naphthalene-2-sulfonic acid | 143.251859623828 | 144.692195109361 | 161.143354891545 | 7.96567013251422 | 5.66602626535678 | 9.02612294940917 | 45.1045734082755 | 78.6389845398503 | 31.7886108528483 | 142.257579647425 | 185.012813222384 | 44.849979296913 | 18.0722673046287 | 22.4291335689652 | 26.0645437489619 |
| Myxopyronin A | 4.36823400057871 | 5.8541798796116 | 1.97390653661027 | 14.2988858257806 | 15.7229060419931 | 11.9799501561401 | 0.604927672732395 | 1.04198920234587 | 2.7924018164222 | 2.65128221225244 | 3.3091266490848 | 7.94043935231981 | 3.03007872098236 | 2.52450874892613 | 2.61369750513718 |
| Monanchorin | 0.238196116671215 | 0.312754289706635 | 0.127800475415838 | 0.787032882165698 | 0.742171963850481 | 1.85212469238656 | 0.644331689805823 | 0.369445842955122 | 0.453602093423121 | 1.50327597528017 | 0.349730282565475 | 0.911767331183497 | 3.90910483380912 | 3.5648509369345 | 3.65468694521746 |
| Lophophorine | 0.85191472682609 | 1.05997245348389 | 0.971660538113462 | 1.16877019569973 | 1.06994028364516 | 0.946092987750255 | 0.528171117344768 | 0.56036370205236 | 0.61761244604478 | 0.655124370141601 | 0.589064498528245 | 0.956293460968702 | 0.980705092904137 | 0.968334753100194 | 0.976014325531178 |
| Loliolide | 31.3782348543994 | 39.2486207623232 | 27.7225964013111 | 11.7284106670733 | 9.11343237965777 | 12.8105641941155 | 7.47682377248934 | 5.26992954883815 | 8.76521865265197 | 23.5877586721723 | 26.206378415041 | 14.0611263324814 | 246.104726664213 | 255.864794138939 | 224.828954987514 |
| Ketoleucine | 2.42065524492029 | 3.01576293738469 | 1.68773793845093 | 4.11245161656041 | 4.77994048237722 | 4.24841579566524 | 0.851593793828368 | 1.01724908945251 | 1.85990700838801 | 1.76203895358175 | 2.10126180204689 | 5.5500729669475 | 4.84416119888653 | 3.06075922283643 | 2.29830109294301 |
| Indolo[2,1-b]quinazoline-6,12-dione | 0.0202512581989147 | 0.0150348716390297 | 0.0233763913483923 | 0.299281110107794 | 0.385497906304613 | 0.284983250892971 | 0.0337367310216388 | 0.0192118859354578 | 0.0235692814792386 | 0.176563998028819 | 0.0619127126472606 | 0.268962907026982 | 0.127145177533212 | 0.0701469562178528 | 0.0477919984814985 |
| Hyrtioerectine B | 1.37847516038993 | 2.79839914598284 | 1.76274685792912 | 0.183172426614486 | 0.183732039429478 | 0.223523161882839 | 0.0878737711881042 | 0.115928094659609 | 5.22339163860462 | 1.39268873399017 | 1.64486842711968 | 0.854708369591021 | 1.43801914425537 | 1.94698641080877 | 2.26025617729049 |
| Hymenoflorin | 7.60114426452594 | 11.6334466636792 | 8.92194999532355 | 13.2797976815042 | 14.6650749069708 | 13.4984754530882 | 3.76568562421642 | 6.1777523656704 | 5.48986040260313 | 8.93317475668591 | 12.1880781912816 | 28.9054077198597 | 9.6371099655864 | 7.89373279847404 | 7.80369038417262 |
| Gliocladic acid_130132 | 39.5575793002495 | 30.0246174031773 | 47.8985780002268 | 8.17476268268821 | 7.24097261492356 | 8.60317291112364 | 19.9721992637593 | 18.6517072000289 | 31.7193445492666 | 9.40198649363818 | 11.738426736546 | 7.01403118750692 | 37.2242356535838 | 40.9307255930264 | 35.3684212390163 |
| Gibberellin A63 | 2.15240084320942 | 1.44251563425326 | 1.44332649407753 | 0.0993517043681718 | 0.172625372421382 | 0.126082818596738 | 0.164852643989447 | 0.101478068982349 | 0.408126698350678 | 0.206459201401932 | 0.261299232537192 | 0.0644949939488047 | 0.216929597919303 | 0.264315115125893 | 0.293318139966515 |
| Gibberellin A52 | 15.4306937385315 | 6.9820782697865 | 13.1247493039411 | 1.15874752697533 | 1.27667758313222 | 1.18942430559382 | 1.80854152344393 | 1.54309362545384 | 2.26830199117292 | 1.99643452721837 | 3.06342569992613 | 1.39874703643628 | 4.82131524787707 | 4.86669074675296 | 4.61496210267257 |
| Fiscalin C | 7.65555123957105 | 6.81131437432423 | 8.18572482768503 | 0.961824978313743 | 0.266300480962972 | 0.988626580443248 | 1.8351477067612 | 2.05933095810029 | 1.15432671920346 | 0.555425587032118 | 0.833922174630877 | 0.327030519127812 | 0.591359376675338 | 0.349786708931513 | 1.60392456608001 |
| Drosopterin | 1.11588809842695 | 0.948906593766953 | 1.25425853724852 | 0.412551467429409 | 0.360745153916583 | 0.446693552337914 | 2.15984620426281 | 1.83008471394255 | 3.42112945747351 | 0.425206520692035 | 0.573031664666542 | 0.604002615114987 | 0.533637097756881 | 0.786714630437212 | 0.439206028167807 |
| Dinocton 6 | 0.165602687432963 | 0.365999465116519 | 0.272266353547988 | 0.987280903109046 | 1.23301081015927 | 0.746198201786324 | 0.572932237628032 | 0.540578818592547 | 0.280908585165955 | 0.488893215144786 | 0.36291070307404 | 0.615305388162347 | 3.18674335317455 | 0.0937149178104608 | 0.0487225099926988 |
| destruxin A | 0.111761320250925 | 0.273243940404307 | 0.312701833905408 | 0.209944998029022 | 0.0787689515615202 | 0.507245205152533 | 0.147686992878104 | 0.0478966341021268 | 0.0410193955944004 | 1.18242276342003 | 0.475697863445077 | 0.0838495123479266 | 0.0612778953101867 | 0.0588539671834296 | 0.0662242667812577 |

**Table S3** *Cont.*

| Compound name | A1_1 | A1_2 | A1_3 | B3_1 | B3_2 | B3_3 | C2_1 | C2_2 | C2_3 | C3_1 | C3_2 | C3_3 | M0_1 | M0_2 | M0_3 |
| --- | --- | --- | --- | --- | --- | --- | --- | --- | --- | --- | --- | --- | --- | --- | --- |
| Dehydroascorbic acid | 2.35920136093222 | 2.42239705850716 | 2.75394808727863 | 16.0564706734894 | 11.0515660913841 | 18.7020273477153 | 5.55163112441335 | 3.82642572026915 | 3.02727678440352 | 8.69226090451353 | 3.84526076273118 | 5.98367236420957 | 11.5405242923379 | 9.88850348494488 | 9.48364712318775 |
| cis-2-Hydroxymethyl-3-methylcyclopentanone | 4.0671966098852 | 4.67161930120598 | 2.80237045942799 | 6.65582263704855 | 6.97729528840521 | 6.93049255277572 | 1.21981844277666 | 1.38183967100388 | 2.15830929042657 | 2.76358462047132 | 2.62455896344503 | 4.12105694097217 | 4.02726696236851 | 3.73210494271004 | 3.28566774904893 |
| Chloriolide | 4.29416859149698 | 4.2036002100973 | 5.2054827375453 | 3.45727841853997 | 3.55683977616337 | 3.79837163776683 | 4.42917949657466 | 4.14939269777648 | 8.40050830537972 | 3.11066382156777 | 2.80517475680226 | 2.19718218162287 | 5.10055102486198 | 5.06222858922877 | 5.36920127307288 |
| Aigialone | 0.115200756664722 | 0.100461137602087 | 0.0790731909991179 | 0.293133448943192 | 0.629018760645836 | 0.619300706803772 | 0.0355728711742503 | 0.0811548631539914 | 0.187417614023795 | 0.274655488269634 | 0.10428839787866 | 0.28479170461234 | 0.506641158986061 | 0.445803832639879 | 0.373710737539563 |
| 9(S)-HpOTrE | 3.11366466855242 | 2.9920849677257 | 3.38672867717445 | 18.8951412064917 | 12.1005157097612 | 19.1951756653639 | 5.07440718962318 | 3.28890784711962 | 4.73396703115118 | 8.50185502790104 | 3.80032769419685 | 3.54331035406513 | 2.8742102733624 | 2.29363588245076 | 3.57914396866251 |
| 9(S)-HOTrE | 32.6405582970499 | 10.1233704180657 | 42.2499549551605 | 13.0496825856025 | 13.2298925615198 | 9.41436577978144 | 21.5001015778593 | 14.747943892134 | 49.810856092298 | 7.38522911581333 | 15.0965066091766 | 5.66118103265965 | 7.92848231300241 | 9.84561635902513 | 16.5080303987851 |
| 4-Nitrophenol | 19.7120123281019 | 22.8578768452671 | 32.5139670405246 | 44.8542378539559 | 42.6109474239346 | 34.722337632097 | 20.1713568236165 | 13.7265649788735 | 19.0290775349223 | 7.92038628828333 | 5.9655870910892 | 7.53659881339517 | 16.1366091 | 15.8758912549972 | 15.0831438420607 |
| 4-Isopropylbenzyl alcohol | 17.6874051088873 | 17.3145341584609 | 16.5865477406532 | 20.9464117237267 | 17.7978288197069 | 21.3885269763171 | 12.6905674890605 | 10.6181235486669 | 16.416445276356 | 17.710183107394 | 28.1198986606498 | 12.5703590659445 | 26.5346159010269 | 28.6765089581068 | 27.1226473931991 |
| 3-Methylsalicylic acid | 0.15917648475031 | 0.384050798592394 | 0.156616838541796 | 0.373737795779902 | 0.447414072612903 | 0.500803705197438 | 0.126548163456341 | 0.17694242989179 | 0.187540411628552 | 0.187786852033941 | 0.33144180637869 | 0.352914711660217 | 0.521813390917122 | 0.420028775520255 | 0.244103426374141 |
| 3-Methyl-2-oxovaleric acid | 0.784592222277556 | 0.788406399949851 | 0.781220481796583 | 0.410643720901074 | 0.4185589749564 | 0.439902927858588 | 0.175043182315253 | 0.24785004690814 | 0.137313559347929 | 0.281144426463331 | 0.365562249713781 | 0.0937819955870491 | 0.298158597590532 | 0.38411411672645 | 0.305736296150515 |
| 3,4-Dihydroxyhydrocinnamic acid | 3.46889592184344 | 4.06310416338818 | 2.9114515229141 | 10.0362955192894 | 11.196982465726 | 9.04991191315618 | 1.61964575474626 | 1.90001922378336 | 2.34271489114556 | 4.15191811335753 | 3.00612769459747 | 4.23861676438649 | 5.19172277954083 | 6.73908433194203 | 5.031140551 |
| 2-Hydroxycampholonic acid | 1.44771767148039 | 1.5391459390824 | 0.722061431112632 | 13.8420172126695 | 12.1694230322813 | 13.5773827483945 | 0.729991532785753 | 0.392293549756821 | 0.629405206574425 | 4.02965676231411 | 1.09127338289666 | 1.35213794313237 | 6.8536715810863 | 7.70148110550889 | 3.55589728606065 |
| 2-Aminophenoxazin-3-one | 0.205687095385176 | 0.243443726359217 | 0.290424780463173 | 0.0764534130868557 | 0.0713226751872728 | 0.426077553358034 | 0.0748569228081338 | 0.124066833250697 | 1.41513985085538 | 0.204587858504829 | 0.151933826436447 | 0.147464099112935 | 0.382383810813753 | 0.229813416546451 | 0.524015393767708 |
| 1-METHYL-HYDANTOIN | 1.61726912 | 1.895779992 | 1.68295698568943 | 5.91425730341056 | 5.83853194683889 | 6.96509889765159 | 1.51384458314868 | 0.0209023945537982 | 1.95100755161111 | 3.88198496229439 | 2.61393558093525 | 4.43121750608337 | 9.44120810933496 | 8.43532531226943 | 5.76612589602345 |
| 1-Methoxyphenanthrene | 0.503453261617892 | 0.520103142989914 | 0.611445991206964 | 7.20539693484099 | 4.19921877383729 | 15.117055757527 | 1.08483740452772 | 0.355388853414738 | 0.441754275419783 | 1.94799856977363 | 0.29190471686717 | 0.341729122738412 | 0.920406341824213 | 1.4564414732828 | 1.06834479816661 |
| 1-Hydroxy-2-naphthoate | 0.341161087943427 | 0.48519302622238 | 0.347214542502682 | 0.688407269893198 | 0.730433494673408 | 0.659793571313256 | 0.147992516867502 | 0.123840600184619 | 0.281794114788041 | 0.464053037103144 | 0.342386401676172 | 0.982992498952108 | 1.01049613461814 | 0.950696718749821 | 0.285823845909932 |
| 13-OxoODE | 36.2418725428043 | 38.1366031271251 | 41.7324216151861 | 34.2997878169739 | 41.6815816413647 | 30.2826493877729 | 37.9128922060063 | 25.90221061 | 27.236242896262 | 42.3072319784935 | 34.9575880976038 | 36.9857190571224 | 19.5964508559016 | 15.9441507604625 | 28.7350950424816 |
| 1,8-Diazacyclotetradecane-2,9-dione | 10.7684092685619 | 11.5781351336127 | 13.5591361119833 | 1.75620957240712 | 1.84511994204773 | 2.24844452589479 | 10.2735704858675 | 16.239507876807 | 9.85169741486548 | 8.51080879181253 | 11.142645821954 | 13.4482580062846 | 2.8820250110771 | 6.20761656056268 | 3.85881186028439 |
| (ent-6alpha,7alpha)-6,7-Dihydroxy-16-kauren-19-oic acid | 5.75835955810424 | 5.09634811688148 | 5.6061888715556 | 6.28127049656618 | 4.28871693684798 | 3.03324650747644 | 5.07642379423442 | 5.37743673689721 | 3.42930680756755 | 9.32084217251427 | 8.45628888753614 | 25.8836225709962 | 6.53933665266467 | 6.33439086240914 | 7.53974148340178 |
| (1R,6R)-6-Hydroxy-2-succinylcyclohexa-2,4-diene-1-carboxylate | 1.75448643673419 | 1.28227231047689 | 2.12667128311748 | 0.0306491687250988 | 0.588560104766271 | 0.547655851658837 | 1.54005694952741 | 1.20703110206007 | 4.60011685238275 | 0.193302812418123 | 0.00921780554327931 | 0.0837218492366154 | 0.067301106668625 | 0.0265601669714221 | 0.0353781265777729 |

Table S4 Identification and classification information of compounds

| name | kegg_id | CAS | IonMode | Formula | MolecularWeight | m/z | Mass Error | Adduct | RT (min) | Score | Level | ClassI | ClassII | ClassIII | ClassⅣ |
| --- | --- | --- | --- | --- | --- | --- | --- | --- | --- | --- | --- | --- | --- | --- | --- |
| Norvaline | C01799 | 760-78-1 | P | C5H11NO2 | 117.07898 | 118.0866 | 2.74908 | [M+H]+ | 1.625 | 0.75 | 1 | Amino acids, amino acid derivatives | Organic acids and derivatives | Carboxylic acids and derivatives | Amino acids, peptides, and analogues |
| N-Lactoyl-Phenylalanine | NA | 183241-73-8 | N | C12H15NO4 | 237.10011 | 236.09297 | 0.68509 | [M-H]- | 5.671 | 0.83 | 1 | Amino acids, amino acid derivatives | Organic acids and derivatives | Carboxylic acids and derivatives | Amino acids, peptides, and analogues |
| N-acetylvaline | NA | 96-81-1 | P | C7H13NO3 | 159.08954 | 182.07909 | 2.21606 | [M+Na]+ | 4.801 | 0.76 | 1 | Amino acids, amino acid derivatives | Organic acids and derivatives | Carboxylic acids and derivatives | Amino acids, peptides, and analogues |
| N-acetylphenylalanine | C03519 | 2018-61-3 | P | C11H13NO3 | 207.08954 | 208.0968 | 0.19575 | [M+H]+ | 5.621 | 0.56 | 1 | Amino acids, amino acid derivatives | Organic acids and derivatives | Carboxylic acids and derivatives | Amino acids, peptides, and analogues |
| N-acetylleucine | C02710 | 1188-21-2 | P | C8H15NO3 | 173.10519 | 196.09473 | 1.97857 | [M+Na]+ | 5.333 | 0.58 | 1 | Amino acids, amino acid derivatives | Organic acids and derivatives | Carboxylic acids and derivatives | Amino acids, peptides, and analogues |
| L-Norleucine | C01933 | 327-57-1 | P | C6H13NO2 | 131.09463 | 132.10189 | 0.30937 | [M+H]+ | 3.402 | 0.83 | 1 | Amino acids, amino acid derivatives | Organic acids and derivatives | Carboxylic acids and derivatives | Amino acids, peptides, and analogues |
| L-Isoleucine | C00407 | 73-32-5 | P | C6H13NO2 | 131.09463 | 132.10197 | 0.33435 | [M+H]+ | 3.762 | 0.82 | 1 | Amino acids, amino acid derivatives | Organic acids and derivatives | Carboxylic acids and derivatives | Amino acids, peptides, and analogues |
| L-Cysteic acid | C00506 | 23537-25-9 | P | C3H7NO5S | 169.00449 | 191.99273 | 5.66509 | [M+Na]+ | 2.983 | 0.62 | 1 | Amino acids, amino acid derivatives | Organic acids and derivatives | Carboxylic acids and derivatives | Amino acids, peptides, and analogues |
| Kainic acid | C12819 | 487-79-6 | P | C10H15NO4 | 213.10011 | 236.0896 | 1.3566 | [M+Na]+ | 5.304 | 0.56 | 1 | Organic acids | Organic acids and derivatives | Carboxylic acids and derivatives | Amino acids, peptides, and analogues |
| Creatinine | C00791 | 60-27-5 | P | C4H7N3O | 113.05891 | 114.06647 | 2.31834 | [M+H]+ | 2.521 | 0.73 | 1 | Amines | Organic acids and derivatives | Carboxylic acids and derivatives | Amino acids, peptides, and analogues |
| beta-cyano-L-Alanine | C02512 | 6232-19-5 | P | C4H6N2O2 | 114.04293 | 115.05058 | 3.09067 | [M+H]+ | 0.883 | 0.52 | 1 | Amino acids, amino acid derivatives | Organic acids and derivatives | Carboxylic acids and derivatives | Amino acids, peptides, and analogues |
| 5-oxoproline | C01879 | 149-87-1 | P | C5H7NO3 | 129.04259 | 130.04999 | 0.8071 | [M+H]+ | 1.915 | 0.89 | 1 | Amino acids, amino acid derivatives | Organic acids and derivatives | Carboxylic acids and derivatives | Amino acids, peptides, and analogues |

**Table S4** *Cont.*

| name | kegg_id | CAS | IonMode | Formula | MolecularWeight | m/z | Mass Error | Adduct | RT (min) | Score | Level | ClassI | ClassII | ClassIII | ClassⅣ |
| --- | --- | --- | --- | --- | --- | --- | --- | --- | --- | --- | --- | --- | --- | --- | --- |
| 5-Hydroxylysine | C16741 | 13204-98-3 | P | C6H14N2O3 | 162.10044 | 185.08965 | 0.06327 | [M+Na]+ | 2.665 | 0.65 | 1 | Amino acids, amino acid derivatives | Organic acids and derivatives | Carboxylic acids and derivatives | Amino acids, peptides, and analogues |
| Vitamin U | C04078 | 1115-84-0 | P | C6H13NO2S | 163.0667 | 181.10006 | 2.67082 | [M+NH4]+ | 5.749 | 0.58 | 2 | Amino acids, amino acid derivatives | Organic acids and derivatives | Carboxylic acids and derivatives | Amino acids, peptides, and analogues |
| L-Cysteinesulfinic acid | C00606 | 1115-65-7 | P | C3H7NO4S | 153.00958 | 171.04305 | 2.16059 | [M+NH4]+ | 4.571 | 0.67 | 2 | Amino acids, amino acid derivatives | Organic acids and derivatives | Carboxylic acids and derivatives | Amino acids, peptides, and analogues |
| Dopaquinone | C00822 | - | P | C9H9NO4 | 195.05316 | 196.0607 | 1.23525 | [M+H]+ | 5.448 | 0.51 | 2 | Amino acids, amino acid derivatives | Organic acids and derivatives | Carboxylic acids and derivatives | Amino acids, peptides, and analogues |
| trans-urocanate | C00785 | 104-98-3 | P | C6H6N2O2 | 138.04293 | 121.03975 | 0.88328 | [M+H-H2O]+ | 2.305 | 1 | 1 | Organic acids | Organoheterocyclic compounds | Azoles | Imidazoles |
| Thymidine | C00214 | 50-89-5 | P | C10H14N2O5 | 242.09027 | 265.07967 | 0.83346 | [M+Na]+ | 4.873 | 0.93 | 1 | Nucleosides, ribose, nucleotides and their derivatives | Nucleosides, nucleotides, and analogues | Pyrimidine nucleosides | Pyrimidine 2'-deoxyribonucleosides |
| N6-Methyl-2'-deoxyadenosine | C03795 | 2002-35-9 | P | C11H15N5O3 | 265.11749 | 266.12494 | 0.57256 | [M+H]+ | 4.888 | 0.97 | 1 | Nucleosides, ribose, nucleotides and their derivatives | Nucleosides, nucleotides, and analogues | Purine nucleosides | Purine 2'-deoxyribonucleosides |
| inosine | C00294 | 58-63-9 | P | C10H12N4O5 | 268.08077 | 291.07005 | 0.29997 | [M+Na]+ | 3.618 | 0.56 | 1 | Nucleosides, ribose, nucleotides and their derivatives | Nucleosides, nucleotides, and analogues | Purine nucleosides | - |
| Guanosine | C00387 | 118-00-3 | P | C10H13N5O5 | 283.09167 | 284.09903 | 0.20808 | [M+H]+ | 3.618 | 0.68 | 1 | Nucleosides, ribose, nucleotides and their derivatives | Nucleosides, nucleotides, and analogues | Purine nucleosides | - |
| Adenosine | C00212 | 58-61-7 | P | C10H13N5O4 | 267.09676 | 268.10395 | 0.42233 | [M+H]+ | 3.2 | 1 | 1 | Nucleosides, ribose, nucleotides and their derivatives | Nucleosides, nucleotides, and analogues | Purine nucleosides | - |
| 3-Methyluridine | NA | 2140-69-4 | N | C10H14N2O6 | 258.08519 | 239.06732 | 0.25949 | [M-H-H2O]- | 6.728 | 0.54 | 1 | Nucleosides, ribose, nucleotides and their derivatives | Nucleosides, nucleotides, and analogues | Pyrimidine nucleosides | - |

**Table S4** *Cont.*

| name | kegg_id | CAS | IonMode | Formula | MolecularWeight | m/z | Mass Error | Adduct | RT (min) | Score | Level | ClassI | ClassII | ClassIII | ClassⅣ |
| --- | --- | --- | --- | --- | --- | --- | --- | --- | --- | --- | --- | --- | --- | --- | --- |
| 2'-O-Methyladenosine | NA | 2140-79-6 | P | C11H15N5O4 | 281.11241 | 282.11993 | 0.76717 | [M+H]+ | 4.801 | 0.71 | 1 | Nucleosides, ribose, nucleotides and their derivatives | Nucleosides, nucleotides, and analogues | Purine nucleosides | - |
| 2'-Deoxyuridine | C00526 | 951-78-0 | P | C9H12N2O5 | 228.07462 | 251.06393 | 0.49105 | [M+Na]+ | 3.091 | 0.54 | 1 | Nucleosides, ribose, nucleotides and their derivatives | Nucleosides, nucleotides, and analogues | Pyrimidine nucleosides | Pyrimidine 2'-deoxyribonucleosides |
| 2'-Deoxyguanosine | C00330 | 961-07-9 | P | C10H13N5O4 | 267.09676 | 268.10398 | 0.29811 | [M+H]+ | 4.376 | 0.91 | 1 | Nucleosides, ribose, nucleotides and their derivatives | Nucleosides, nucleotides, and analogues | Purine nucleosides | Purine 2'-deoxyribonucleosides |
| 2'-Deoxycytidine | C00881 | 951-77-9 | P | C9H13N3O4 | 227.09061 | 250.08004 | 1.02512 | [M+Na]+ | 1.915 | 0.68 | 1 | Nucleosides, ribose, nucleotides and their derivatives | Nucleosides, nucleotides, and analogues | Pyrimidine nucleosides | Pyrimidine 2'-deoxyribonucleosides |
| 2'-Deoxyadenosine | C00559 | 958-09-8 | P | C10H13N5O3 | 251.10184 | 252.10894 | 0.79791 | [M+H]+ | 3.647 | 1 | 1 | Nucleosides, ribose, nucleotides and their derivatives | Nucleosides, nucleotides, and analogues | Purine nucleosides | Purine 2'-deoxyribonucleosides |
| N,N-Dimethylguanosine | NA | - | P | C12H17N5O5 | 311.12297 | 312.13015 | 0.37014 | [M+H]+ | 5.003 | 0.5 | 2 | Nucleosides, ribose, nucleotides and their derivatives | Nucleosides, nucleotides, and analogues | Purine nucleosides | - |
| N(6)-OH-Me-Adenosine | NA | - | P | C11H15N5O5 | 297.10732 | 298.11483 | 0.7082 | [M+H]+ | 4.869 | 0.75 | 2 | Nucleosides, ribose, nucleotides and their derivatives | Nucleosides, nucleotides, and analogues | Purine nucleosides | - |
| 9-Riburonosyladenine | C11501 | - | P | C10H11N5O5 | 281.07602 | 282.08336 | 0.15259 | [M+H]+ | 4.614 | 0.66 | 2 | Nucleosides, ribose, nucleotides and their derivatives | Nucleosides, nucleotides, and analogues | Purine nucleosides | - |
| 5'-N-Methylcarboxamidoadenosine | NA | - | P | C11H14N6O4 | 294.10765 | 295.11569 | 2.5034 | [M+H]+ | 5.591 | 0.53 | 2 | Nucleosides, ribose, nucleotides and their derivatives | Nucleosides, nucleotides, and analogues | Purine nucleosides | - |

**Table S4** *Cont.*

| name | kegg_id | CAS | IonMode | Formula | MolecularWeight | m/z | Mass Error | Adduct | RT (min) | Score | Level | ClassI | ClassII | ClassIII | ClassⅣ |
| --- | --- | --- | --- | --- | --- | --- | --- | --- | --- | --- | --- | --- | --- | --- | --- |
| 5'-Dehydroadenosine | C11500 | - | P | C10H11N5O4 | 265.08111 | 266.08843 | 0.09403 | [M+H]+ | 3.963 | 0.8 | 2 | Nucleosides, ribose, nucleotides and their derivatives | Nucleosides, nucleotides, and analogues | Purine nucleosides | - |
| 5,6-Dihydrouridine | NA | 1361384 | P | C9H14N2O6 | 246.08519 | 269.07377 | 2.50998 | [M+Na]+ | 5.99 | 0.53 | 2 | Nucleosides, ribose, nucleotides and their derivatives | Nucleosides, nucleotides, and analogues | Pyrimidine nucleosides | - |
| 2'-Amino-2'-deoxyadenosine | NA | 10414-81-0 | N | C10H14N6O3 | 266.11274 | 265.10607 | 2.38143 | [M-H]- | 5.757 | 0.77 | 2 | Nucleosides, ribose, nucleotides and their derivatives | Nucleosides, nucleotides, and analogues | Purine nucleosides | Purine 2'-deoxyribonucleosides |
| Sphingosine (d17:1) | NA | 6918-48-5 | P | C17H35NO2 | 285.26678 | 308.2563 | 1.13779 | [M+Na]+ | 7.725 | 0.66 | 1 | Lipids and lipid like substances | Organic nitrogen compounds | Organonitrogen compounds | Amines |
| Phytosphingosine | C12144 | 554-62-1 | P | C18H39NO3 | 317.29299 | 340.28223 | 0.11367 | [M+Na]+ | 6.677 | 0.52 | 1 | Lipids and lipid like substances | Organic nitrogen compounds | Organonitrogen compounds | Amines |
| oleoyl ethanolamide | C20792 | 111-58-0 | P | C20H39NO2 | 325.29808 | 348.28729 | 0.0204 | [M+Na]+ | 8.099 | 0.58 | 1 | Amines | Organic nitrogen compounds | Organonitrogen compounds | Amines |
| N-Feruloylserotonin | NA | 68573-23-9 | P | C20H20N2O4 | 352.14231 | 375.131 | 1.44207 | [M+Na]+ | 6.604 | 0.61 | 1 | Amines | Organoheterocyclic compounds | Indoles and derivatives | Tryptamines and derivatives |
| Histamine | C00388 | 51-74-1 | P | C5H9N3 | 111.07965 | 112.08728 | 2.97083 | [M+H]+ | 1.875 | 0.66 | 1 | Amines | Organic nitrogen compounds | Organonitrogen compounds | Amines |
| 2-Amino-1-phenylethanol | C02735 | 7568-93-6 | P | C8H11NO | 137.08406 | 120.08095 | 1.35661 | [M+H-H2O]+ | 4.989 | 0.93 | 1 | Alcohols | Organic nitrogen compounds | Organonitrogen compounds | Amines |
| Xestoaminol C | NA | - | P | C14H31NO | 229.24056 | 230.24803 | 0.76195 | [M+H]+ | 6.255 | 0.61 | 2 | Alcohols | Organic nitrogen compounds | Organonitrogen compounds | Amines |
| Serotonin | C00780 | 50-67-9 | P | C10H12N2O | 176.09496 | 159.09259 | 5.27523 | [M+H-H2O]+ | 0.684 | 0.81 | 2 | Amines | Organoheterocyclic compounds | Indoles and derivatives | Tryptamines and derivatives |
| Palmitoleoyl Ethanolamide | NA | - | P | C18H35NO2 | 297.26678 | 320.25592 | 0.21205 | [M+Na]+ | 7.465 | 0.79 | 2 | Amines | Organic nitrogen compounds | Organonitrogen compounds | Amines |
| N,N-Dimethylaniline | C02846 | 121-69-7 | P | C8H11N | 121.08915 | 122.09668 | 1.93346 | [M+H]+ | 4.816 | 0.68 | 2 | Amines | Organic nitrogen compounds | Organonitrogen compounds | Amines |
| Taurodeoxycholate | C05463 | 1180-95-6 | P | C26H45NO6S | 499.29676 | 522.28623 | 0.53833 | [M+Na]+ | 6.867 | 0.88 | 1 | Organic acids | Lipids and lipid-like molecules | Steroids and steroid derivatives | Bile acids, alcohols and derivatives |
| Taurocholic acid | C05122 | 81-24-3 | N | C26H45NO7S | 515.29168 | 514.28491 | 1.01984 | [M-H]- | 6.465 | 0.93 | 1 | Organic acids | Lipids and lipid-like molecules | Steroids and steroid derivatives | Bile acids, alcohols and derivatives |
| Taurochenodeoxycholic acid | C05465 | 516-35-8 | N | C26H45NO6S | 499.29676 | 498.29012 | 1.31902 | [M-H]- | 6.859 | 1 | 1 | Organic acids | Lipids and lipid-like molecules | Steroids and steroid derivatives | Bile acids, alcohols and derivatives |

**Table S4** *Cont.*

| name | kegg_id | CAS | IonMode | Formula | MolecularWeight | m/z | Mass Error | Adduct | RT (min) | Score | Level | ClassI | ClassII | ClassIII | ClassⅣ |
| --- | --- | --- | --- | --- | --- | --- | --- | --- | --- | --- | --- | --- | --- | --- | --- |
| Glycocholic acid | C01921 | 475-31-0 | P | C26H43NO6 | 465.30904 | 488.29829 | 0.0977 | [M+Na]+ | 6.721 | 0.87 | 1 | Organic acids | Lipids and lipid-like molecules | Steroids and steroid derivatives | Bile acids, alcohols and derivatives |
| Glycochenodeoxycholic acid | C05466 | 640-79-9 | N | C26H43NO5 | 449.31412 | 448.30727 | 0.99769 | [M-H]- | 7.165 | 0.95 | 1 | Organic acids | Lipids and lipid-like molecules | Steroids and steroid derivatives | Bile acids, alcohols and derivatives |
| 5alpha-Cholestan-3-one | C03238 | 566-88-1 | P | C27H46O | 386.35487 | 369.35098 | 1.52438 | [M+H-H2O]+ | 10.329 | 0.98 | 2 | Steroid hormones, steroids | Lipids and lipid-like molecules | Steroids and steroid derivatives | Cholestane steroids |
| Ureidopropionic acid | C02642 | 462-88-4 | P | C4H8N2O3 | 132.05349 | 155.04302 | 2.47366 | [M+Na]+ | 1.509 | 0.69 | 1 | Organic acids | Organic acids and derivatives | Organic carbonic acids and derivatives | Ureas |
| 1,3,7-Trimethyluric acid | C16361 | 5415-44-1 | P | C8H10N4O3 | 210.07529 | 211.08217 | 1.97635 | [M+H]+ | 5.821 | 0.55 | 1 | Organic acids | Organoheterocyclic compounds | Imidazopyrimidines | Purines and purine derivatives |
| Xanthine | C00385 | 69-89-6 | P | C5H4N4O2 | 152.03343 | 153.04109 | 2.36982 | [M+H]+ | 5.233 | 0.51 | 2 | Nucleosides, ribose, nucleotides and their derivatives | Organoheterocyclic compounds | Imidazopyrimidines | Purines and purine derivatives |
| Thymine | C00178 | 65-71-4 | P | C5H6N2O2 | 126.04293 | 127.05038 | 1.20844 | [M+H]+ | 4.873 | 0.92 | 2 | Nucleosides, ribose, nucleotides and their derivatives | Organoheterocyclic compounds | Diazines | Pyrimidines and pyrimidine derivatives |
| cytosine | C00380 | 71-30-7 | P | C4H5N3O | 111.04326 | 112.05092 | 3.24911 | [M+H]+ | 1.915 | 0.88 | 2 | Nucleosides, ribose, nucleotides and their derivatives | Organoheterocyclic compounds | Diazines | Pyrimidines and pyrimidine derivatives |
| Alloxan | C07599 | 50-71-5 | P | C4H2N2O4 | 142.00146 | 143.00862 | 1.01022 | [M+H]+ | 1.573 | 0.59 | 2 | Nucleosides, ribose, nucleotides and their derivatives | Organoheterocyclic compounds | Diazines | Pyrimidines and pyrimidine derivatives |
| 8-Chloroxanthine | NA | 13548-68-0 | P | C5H3ClN4O2 | 185.99445 | 187.00243 | 3.63686 | [M+H]+ | 4.742 | 0.6 | 2 | Nucleosides, ribose, nucleotides and their derivatives | Organoheterocyclic compounds | Imidazopyrimidines | Purines and purine derivatives |
| 5,6-Dihydroxyuracil | NA | - | P | C4H4N2O4 | 144.01711 | 145.0244 | 0.09431 | [M+H]+ | 1.958 | 0.58 | 2 | Pyrimidines | Organoheterocyclic compounds | Diazines | Pyrimidines and pyrimidine derivatives |
| 3,7-Dimethylguanine | NA | - | P | C7H9N5O | 179.08071 | 180.08715 | 4.80519 | [M+H]+ | 5.749 | 0.55 | 2 | Nucleosides, ribose, nucleotides and their derivatives | Organoheterocyclic compounds | Imidazopyrimidines | Purines and purine derivatives |
| N-Cyclohexylformamide | C11519 | 766-93-8 | P | C7H13NO | 127.09971 | 128.10721 | 1.61073 | [M+H]+ | 5.018 | 0.9 | 1 | Amines | Organic acids and derivatives | Carboxylic acids and derivatives | Carboxylic acid derivatives |

**Table S4** *Cont.*

| name | kegg_id | CAS | IonMode | Formula | MolecularWeight | m/z | Mass Error | Adduct | RT (min) | Score | Level | ClassI | ClassII | ClassIII | ClassⅣ |
| --- | --- | --- | --- | --- | --- | --- | --- | --- | --- | --- | --- | --- | --- | --- | --- |
| N-Acetylmuramate | C02713 | 10597-89-4 | P | C11H19NO8 | 293.11107 | 276.10789 | 0.41358 | [M+H-H2O]+ | 4.83 | 0.9 | 1 | Amines | Organic oxygen compounds | Organooxygen compounds | Carbohydrates and carbohydrate conjugates |
| Methyl beta-D-Galactopyranoside | C03619 | 1824-94-8 | P | C7H14O6 | 194.07904 | 217.06858 | 1.73279 | [M+Na]+ | 1.509 | 0.7 | 1 | Sugar derivative | Organic oxygen compounds | Organooxygen compounds | Carbohydrates and carbohydrate conjugates |
| D-Arabinono-1,4-lactone | C00652 | 322392 | P | C5H8O5 | 148.03717 | 171.02622 | 0.97978 | [M+Na]+ | 0.618 | 0.8 | 1 | Sugar derivative | Organic oxygen compounds | Organooxygen compounds | Carbohydrates and carbohydrate conjugates |
| MUCIC ACID | C00879 | 526-99-8 | P | C6H10O8 | 210.03757 | 211.04534 | 2.22477 | [M+H]+ | 1.188 | 0.54 | 2 | Organic acids | Organic oxygen compounds | Organooxygen compounds | Carbohydrates and carbohydrate conjugates |
| D-Mannitol 1-phosphate | C00644 | - | P | C6H15O9P | 262.04537 | 263.05292 | 0.93781 | [M+H]+ | 5.347 | 0.53 | 2 | Sugar derivative | Organic oxygen compounds | Organooxygen compounds | Carbohydrates and carbohydrate conjugates |
| D-Gluconic Acid | C00257 | 526-95-4 | P | C6H12O7 | 196.05831 | 219.04785 | 1.73905 | [M+Na]+ | 5.98 | 0.69 | 2 | Sugar derivative | Organic oxygen compounds | Organooxygen compounds | Carbohydrates and carbohydrate conjugates |
| alpha-Cyclogeraniol acetate | NA | - | P | C13H22O2 | 210.16198 | 211.16947 | 0.9264 | [M+H]+ | 0.838 | 0.71 | 2 | Alcohols | Organic acids and derivatives | Carboxylic acids and derivatives | Carboxylic acid derivatives |
| 1-Cyclohexenecarboxylic acid | C12101 | 636-82-8 | P | C7H10O2 | 126.06808 | 127.07553 | 1.15842 | [M+H]+ | 0.676 | 0.74 | 2 | Organic acids | Organic acids and derivatives | Carboxylic acids and derivatives | Carboxylic acids |
| Prostaglandin E1 | C04741 | 745-65-3 | P | C20H34O5 | 354.24063 | 377.22965 | 0.52223 | [M+Na]+ | 7.086 | 0.59 | 1 | Lipids and lipid like substances | Lipids and lipid-like molecules | Fatty Acyls | Eicosanoids |
| all-trans-4-Oxoretinoic acid | C16678 | 38030-57-8 | P | C20H26O3 | 314.18819 | 337.17734 | 0.17084 | [M+Na]+ | 7.556 | 0.87 | 1 | Organic acids | Lipids and lipid-like molecules | Prenol lipids | Retinoids |
| 9-cis-Retinoic acid | C15493 | 1241893 | P | C20H28O2 | 300.20893 | 283.20647 | 2.81204 | [M+H-H2O]+ | 8.428 | 0.64 | 1 | Organic acids | Lipids and lipid-like molecules | Prenol lipids | Retinoids |
| 2,5-Furandicarboxylic acid | C20450 | 3238-40-2 | P | C6H4O5 | 156.00588 | 178.99507 | 0.06917 | [M+Na]+ | 2.132 | 0.78 | 2 | Organic acids | Organoheterocyclic compounds | Furans | Furoic acid and derivatives |
| 13,14-Dihydro-15-keto-PGE2 | C04671 | 363-23-5 | N | C20H32O5 | 352.22498 | 351.21684 | 2.37228 | [M-H]- | 6.772 | 0.53 | 2 | Lipids and lipid like substances | Lipids and lipid-like molecules | Fatty Acyls | Eicosanoids |
| Vitamin B5 | C00864 | 79-83-4 | P | C9H17NO5 | 219.11067 | 242.10013 | 1.18004 | [M+Na]+ | 5.047 | 0.74 | 1 | Organic acids | Organic oxygen compounds | Organooxygen compounds | Alcohols and polyols |
| Undecylenic acid | C13910 | 112-38-9 | N | C11H20O2 | 184.14633 | 229.14459 | 0.31331 | [M+HCOOH-H]- | 5.829 | 0.58 | 1 | Organic acids | Lipids and lipid-like molecules | Fatty Acyls | Fatty acids and conjugates |
| Traumatic Acid | C16308 | 6402-36-4 | N | C12H20O4 | 228.13616 | 227.12881 | 0.21052 | [M-H]- | 6.291 | 0.63 | 1 | Organic acids | Lipids and lipid-like molecules | Fatty Acyls | Fatty acids and conjugates |
| trans-vaccenic acid | C08367 | 693-72-1 | N | C18H34O2 | 282.25588 | 327.254 | 0.28432 | [M+HCOOH-H]- | 9.166 | 0.77 | 1 | Organic acids | Lipids and lipid-like molecules | Fatty Acyls | Fatty acids and conjugates |

**Table S4** *Cont.*

| name | kegg_id | CAS | IonMode | Formula | MolecularWeight | m/z | Mass Error | Adduct | RT (min) | Score | Level | ClassI | ClassII | ClassIII | ClassⅣ |
| --- | --- | --- | --- | --- | --- | --- | --- | --- | --- | --- | --- | --- | --- | --- | --- |
| Tetradecanoic acid | C06424 | 544-63-8 | N | C14H28O2 | 228.20893 | 227.20147 | 0.70601 | [M-H]- | 9.342 | 0.67 | 1 | Organic acids | Lipids and lipid-like molecules | Fatty Acyls | Fatty acids and conjugates |
| Suberic acid | C08278 | 505-48-6 | N | C8H14O4 | 174.08921 | 173.08146 | 2.57116 | [M-H]- | 5.628 | 0.64 | 1 | Organic acids | Lipids and lipid-like molecules | Fatty Acyls | Fatty acids and conjugates |
| Sebacic acid | C08277 | 111-20-6 | N | C10H18O4 | 202.12051 | 201.113 | 1.03948 | [M-H]- | 5.988 | 0.62 | 1 | Organic acids | Lipids and lipid-like molecules | Fatty Acyls | Fatty acids and conjugates |
| Sabinic acid | C08317 | 505-95-3 | P | C12H24O3 | 216.17255 | 199.16941 | 0.72484 | [M+H-H2O]+ | 6.125 | 0.77 | 1 | Organic acids | Organic acids and derivatives | Hydroxy acids and derivatives | Medium-chain hydroxy acids and derivatives |
| Ricinoleic acid | C08365 | 141-22-0 | N | C18H34O3 | 298.2508 | 297.24357 | 0.24983 | [M-H]- | 8.036 | 0.63 | 1 | Organic acids | Lipids and lipid-like molecules | Fatty Acyls | Fatty acids and conjugates |
| Potassium | D05892 | 24634-61-5 | N | C6H8O2 | 112.05243 | 171.0658 | 4.77252 | [M+CH3COOH-H]- | 5.728 | 0.51 | 1 | Organic acids | Lipids and lipid-like molecules | Fatty Acyls | Fatty acids and conjugates |
| Pimelic acid | C02656 | 111-16-0 | N | C7H12O4 | 160.07356 | 159.06576 | 3.10432 | [M-H]- | 5.44 | 0.79 | 1 | Organic acids | Lipids and lipid-like molecules | Fatty Acyls | Fatty acids and conjugates |
| Panthenol | C05944 | 81-13-0 | P | C9H19NO4 | 205.13141 | 206.13889 | 0.86498 | [M+H]+ | 5.061 | 0.98 | 1 | Alcohols | Lipids and lipid-like molecules | Fatty Acyls | Fatty amides |
| Palmitoleic acid | C08362 | 373-49-9 | N | C16H30O2 | 254.22458 | 253.21733 | 0.19856 | [M-H]- | 9.608 | 0.81 | 1 | Alcohols | Lipids and lipid-like molecules | Fatty Acyls | Fatty acids and conjugates |
| Methylsuccinic acid | C08645 | 498-21-5 | P | C5H8O4 | 132.04226 | 115.03931 | 2.6617 | [M+H-H2O]+ | 5.09 | 0.7 | 1 | Organic acids | Lipids and lipid-like molecules | Fatty Acyls | Fatty acids and conjugates |
| L-Rhamnonate | C01934 | 159929-82-5 | P | C6H11O6- | 179.05557 | 203.05283 | 1.31846 | [M+Na]+ | 1.452 | 0.66 | 1 | Organic acids | Organic acids and derivatives | Hydroxy acids and derivatives | Medium-chain hydroxy acids and derivatives |
| Hexadecanedioic acid | C19615 | 505-54-4 | P | C16H30O4 | 286.21441 | 309.20384 | 0.79672 | [M+Na]+ | 7.028 | 0.78 | 1 | Organic acids | Lipids and lipid-like molecules | Fatty Acyls | Fatty acids and conjugates |
| Hendecanoic acid | C17715 | 112-37-8 | N | C11H22O2 | 186.16198 | 231.1602 | 0.12863 | [M+HCOOH-H]- | 6.234 | 0.6 | 1 | Organic acids | Lipids and lipid-like molecules | Fatty Acyls | Fatty acids and conjugates |
| Elaidic acid | C01712 | 112-79-8 | N | C18H34O2 | 282.25588 | 281.24869 | 0.40705 | [M-H]- | 10.422 | 0.51 | 1 | Organic acids | Lipids and lipid-like molecules | Fatty Acyls | Fatty acids and conjugates |
| Dodecanoic acid | C02679 | 143-07-7 | N | C12H24O2 | 200.17763 | 259.1917 | 0.83051 | [M+CH3COOH-H]- | 6.801 | 0.83 | 1 | Organic acids | Lipids and lipid-like molecules | Fatty Acyls | Fatty acids and conjugates |
| Dodecanedioic acid | C02678 | 693-23-2 | N | C12H22O4 | 230.15181 | 229.14446 | 0.22037 | [M-H]- | 6.413 | 0.69 | 1 | Organic acids | Lipids and lipid-like molecules | Fatty Acyls | Fatty acids and conjugates |

**Table S4** *Cont.*

| name | kegg_id | CAS | IonMode | Formula | MolecularWeight | m/z | Mass Error | Adduct | RT (min) | Score | Level | ClassI | ClassII | ClassIII | ClassⅣ |
| --- | --- | --- | --- | --- | --- | --- | --- | --- | --- | --- | --- | --- | --- | --- | --- |
| Docosanoic acid | C08281 | 112-85-6 | N | C22H44O2 | 340.33413 | 339.32683 | 0.0098 | [M-H]- | 10.856 | 0.62 | 1 | Organic acids | Lipids and lipid-like molecules | Fatty Acyls | Fatty acids and conjugates |
| Dethiobiotin | C01909 | 533-48-2 | P | C10H18N2O3 | 214.13174 | 197.12849 | 0.22811 | [M+H-H2O]+ | 5.678 | 0.67 | 1 | Organic acids | Lipids and lipid-like molecules | Fatty Acyls | Fatty acids and conjugates |
| cis-5,8,11,14,17-Eicosapentaenoic acid | C06428 | 10417-94-4 | P | C20H30O2 | 302.22458 | 303.23187 | 0.03696 | [M+H]+ | 7.783 | 0.88 | 1 | Organic acids | Lipids and lipid-like molecules | Fatty Acyls | Fatty acids and conjugates |
| cis-4,7,10,13,16,19-Docosahexaenoic acid | C06429 | 6217-54-5 | N | C22H32O2 | 328.24023 | 327.23307 | 0.41294 | [M-H]- | 9.653 | 0.77 | 1 | Organic acids | Lipids and lipid-like molecules | Fatty Acyls | Fatty acids and conjugates |
| Bombykol | C16873 | 765-17-3 | P | C16H30O | 238.22966 | 256.26321 | 1.05872 | [M+NH4]+ | 9.488 | 0.82 | 1 | Alcohols | Lipids and lipid-like molecules | Fatty Acyls | Fatty alcohols |
| Azelaic acid | C08261 | 123-99-9 | P | C9H16O4 | 188.10486 | 211.09413 | 0.39132 | [M+Na]+ | 5.807 | 0.62 | 1 | Organic acids | Lipids and lipid-like molecules | Fatty Acyls | Fatty acids and conjugates |
| Arachidonic acid | C00219 | 506-32-1 | P | C20H32O2 | 304.24023 | 287.23684 | 0.30057 | [M+H-H2O]+ | 7.841 | 0.77 | 1 | Organic acids | Lipids and lipid-like molecules | Fatty Acyls | Fatty acids and conjugates |
| Adipic acid | C06104 | 124-04-9 | N | C6H10O4 | 146.05791 | 127.03916 | 6.50698 | [M-H-H2O]- | 5.411 | 0.54 | 1 | Organic acids | Lipids and lipid-like molecules | Fatty Acyls | Fatty acids and conjugates |
| 10-Hydroxydecanoate | C02774 | 1679-53-4 | P | C10H20O3 | 188.14124 | 211.13082 | 2.02522 | [M+Na]+ | 5.534 | 0.53 | 1 | Organic acids | Organic acids and derivatives | Hydroxy acids and derivatives | Medium-chain hydroxy acids and derivatives |
| (+/-)-2-Propyl-4-pentenoic acid | C16648 | 1575-72-0 | P | C8H14O2 | 142.09938 | 165.08953 | 6.66709 | [M+Na]+ | 4.758 | 0.92 | 1 | Organic acids | Lipids and lipid-like molecules | Fatty Acyls | Fatty acids and conjugates |
| Octadecanoic acid | C01530 | 21128 | P | C18H36O2 | 284.27153 | 302.3053 | 0.1028 | [M+NH4]+ | 7.276 | 0.73 | 2 | Organic acids | Lipids and lipid-like molecules | Fatty Acyls | Fatty acids and conjugates |
| Heptadecatrienal | C16343 | - | P | C17H28O | 248.21401 | 249.22142 | 0.4379 | [M+H]+ | 7.805 | 0.81 | 2 | Others | Lipids and lipid-like molecules | Fatty Acyls | Fatty aldehydes |
| Estriol | C05141 | 50-27-1 | P | C18H24O3 | 288.17255 | 289.17966 | 0.65444 | [M+H]+ | 6.212 | 0.76 | 2 | Steroid hormones, steroids | Lipids and lipid-like molecules | Steroids and steroid derivatives | Estrane steroids |
| Estrane | C19641 | - | P | C18H30 | 246.23475 | 247.24194 | 0.46203 | [M+H]+ | 9.701 | 0.91 | 2 | Steroid hormones, steroids | Lipids and lipid-like molecules | Steroids and steroid derivatives | Estrane steroids |
| Dodecanamide | C13831 | 112-01-6 | P | C12H25NO | 199.19361 | 200.20093 | 0.0885 | [M+H]+ | 7.685 | 0.93 | 2 | Amines | Lipids and lipid-like molecules | Fatty Acyls | Fatty amides |
| Dihydromonacolin L acid | C20851 | - | P | C19H32O4 | 324.23006 | 325.2367 | 2.02359 | [M+H]+ | 7.101 | 0.78 | 2 | Organic acids | Organic acids and derivatives | Hydroxy acids and derivatives | Medium-chain hydroxy acids and derivatives |

**Table S4** *Cont.*

| name | kegg_id | CAS | IonMode | Formula | MolecularWeight | m/z | Mass Error | Adduct | RT (min) | Score | Level | ClassI | ClassII | ClassIII | ClassⅣ |
| --- | --- | --- | --- | --- | --- | --- | --- | --- | --- | --- | --- | --- | --- | --- | --- |
| Dibutyl adipate | C14253 | 105-99-7 | P | C14H26O4 | 258.18311 | 259.19042 | 0.04883 | [M+H]+ | 7.363 | 0.58 | 2 | Esters | Lipids and lipid-like molecules | Fatty Acyls | Fatty acid esters |
| alpha-Isopropylmalate | C02504 | 3237-44-3 | P | C7H12O5 | 176.06848 | 199.05767 | 0.04489 | [M+Na]+ | 11.314 | 0.68 | 2 | Organic acids | Lipids and lipid-like molecules | Fatty Acyls | Fatty acids and conjugates |
| 9-Oxononanoic acid | C16322 | - | N | C9H16O3 | 172.10995 | 171.10212 | 3.09946 | [M-H]- | 6.103 | 0.52 | 2 | Organic acids | Lipids and lipid-like molecules | Fatty Acyls | Fatty acids and conjugates |
| 9,10-Epoxy-13-hydroxy-11-octadecenoate | C14834 | - | P | C18H32O4 | 312.23006 | 313.23711 | 0.81139 | [M+H]+ | 7.189 | 0.91 | 2 | Esters | Organic acids and derivatives | Hydroxy acids and derivatives | Medium-chain hydroxy acids and derivatives |
| 9,10-Dihydroxystearate | C19622 | - | N | C18H36O4 | 316.26136 | 315.25379 | 0.86844 | [M-H]- | 7.586 | 0.82 | 2 | Esters | Lipids and lipid-like molecules | Fatty Acyls | Fatty acids and conjugates |
| 9,10-DiHOME | C14828 | 263399-34-4 | N | C18H34O4 | 314.24571 | 313.23867 | 0.84244 | [M-H]- | 7.181 | 0.69 | 2 | Organic acids | Lipids and lipid-like molecules | Fatty Acyls | Fatty acids and conjugates |
| 9,10,13-TriHOME | C14835 | - | P | C18H34O5 | 330.24063 | 331.24784 | 0.2649 | [M+H]+ | 6.458 | 0.82 | 2 | Organic acids | Lipids and lipid-like molecules | Fatty Acyls | Fatty acids and conjugates |
| 3-Deoxyestradiol | C14239 | - | P | C18H24O | 256.18272 | 257.18983 | 0.74073 | [M+H]+ | 8.732 | 0.53 | 2 | Steroid hormones, steroids | Lipids and lipid-like molecules | Steroids and steroid derivatives | Estrane steroids |
| 12,13-Epoxy-9-hydroxy-10-octadecenoate | C14832 | - | N | C18H32O4 | 312.23006 | 311.22307 | 0.97789 | [M-H]- | 7.181 | 0.83 | 2 | Organic acids | Lipids and lipid-like molecules | Fatty Acyls | Fatty acids and conjugates |
| 12-(2,3-Dihydroxycyclopentyl)-2-dodecanone | C14996 | - | N | C17H32O3 | 284.23514 | 283.22748 | 1.26401 | [M-H]- | 7.687 | 0.7 | 2 | Alcohols | Organic oxygen compounds | Organooxygen compounds | Alcohols and polyols |
| 11-Aminoundecanoic acid | C19325 | 2432-99-7 | P | C11H23NO2 | 201.17288 | 202.18039 | 1.05579 | [M+H]+ | 5.922 | 0.77 | 2 | Organic acids | Lipids and lipid-like molecules | Fatty Acyls | Fatty acids and conjugates |
| 1,4-Bis(2-ethylhexyl) sulfosuccinate | C07874 | - | N | C20H38O7S | 422.23383 | 421.22677 | 0.56773 | [M-H]- | 8.151 | 0.64 | 2 | Organic acids | Lipids and lipid-like molecules | Fatty Acyls | Fatty acid esters |
| Epiandrosterone | C07635 | 481-29-8 | P | C19H30O2 | 290.22458 | 273.22113 | 0.52784 | [M+H-H2O]+ | 7.667 | 0.68 | 1 | Steroid hormones, steroids | Lipids and lipid-like molecules | Steroids and steroid derivatives | Androstane steroids |
| Biotin sulfone | C20387 | 40720-05-6 | P | C10H16N2O5S | 276.07799 | 299.06751 | 1.16106 | [M+Na]+ | 5.617 | 0.7 | 1 | Others | Organoheterocyclic compounds | Biotin and derivatives | - |
| Adrenosterone | C05285 | 382-45-6 | P | C19H24O3 | 300.17255 | 283.16937 | 0.38958 | [M+H-H2O]+ | 6.779 | 0.71 | 1 | Steroid hormones, steroids | Lipids and lipid-like molecules | Steroids and steroid derivatives | Androstane steroids |
| 21-Hydroxypregnenolone | C05485 | 1164-98-3 | P | C21H32O3 | 332.23514 | 355.22412 | 0.65235 | [M+Na]+ | 8.441 | 0.75 | 1 | Steroid hormones, steroids | Lipids and lipid-like molecules | Steroids and steroid derivatives | Hydroxysteroids |

**Table S4** *Cont.*

| name | kegg_id | CAS | IonMode | Formula | MolecularWeight | m/z | Mass Error | Adduct | RT (min) | Score | Level | ClassI | ClassII | ClassIII | ClassⅣ |
| --- | --- | --- | --- | --- | --- | --- | --- | --- | --- | --- | --- | --- | --- | --- | --- |
| 11beta-Hydroxyprogesterone | C05498 | 600-57-7 | P | C21H30O3 | 330.2195 | 353.2086 | 0.31334 | [M+Na]+ | 8.199 | 0.57 | 1 | Steroid hormones, steroids | Lipids and lipid-like molecules | Steroids and steroid derivatives | Pregnane steroids |
| 11beta-Hydroxyandrosterone | C14606 | 57-61-4 | P | C19H30O3 | 306.2195 | 329.20831 | 1.27383 | [M+Na]+ | 7.624 | 0.56 | 1 | Steroid hormones, steroids | Lipids and lipid-like molecules | Steroids and steroid derivatives | Androstane steroids |
| SM(d18:0/16:1(9Z)) | C00550 | - | P | C39H79N2O6P | 702.56757 | 703.57426 | 0.87155 | [M+H]+ | 10.907 | 0.59 | 2 | Lipids and lipid like substances | Lipids and lipid-like molecules | Sphingolipids | Phosphosphingolipids |
| Pumiliotoxin 251D | C20031 | - | P | C16H29NO | 251.22491 | 252.23222 | 0.04979 | [M+H]+ | 8.341 | 0.73 | 2 | Amines | Alkaloids and derivatives | Pumiliotoxins, homopumiliotoxins, and allopumiliotoxins | - |
| Decarbamoylsaxitoxin | C20021 | - | P | C9H16N6O3 | 256.12839 | 257.13634 | 2.54299 | [M+H]+ | 5.577 | 0.51 | 2 | Amines | Phenylpropanoids and polyketides | Saxitoxins, gonyautoxins, and derivatives | - |
| Tributyrin | C13870 | 21920 | P | C15H26O6 | 302.17294 | 325.16229 | 0.49657 | [M+Na]+ | 6.284 | 0.56 | 1 | Lipids and lipid like substances | Lipids and lipid-like molecules | Glycerolipids | Triradylcglycerols |
| L-Malate | C00149 | 97-67-6 | N | C4H6O5 | 134.02152 | 133.0133 | 6.85978 | [M-H]- | 1.544 | 0.72 | 1 | Organic acids | Organic acids and derivatives | Hydroxy acids and derivatives | Beta hydroxy acids and derivatives |
| D-alpha-Hydroxyglutaric acid | C01087 | 103404-90-6 | P | C5H8O5 | 148.03717 | 131.03405 | 1.20778 | [M+H-H2O]+ | 0.684 | 0.7 | 1 | Amino acids, amino acid derivatives | Organic acids and derivatives | Hydroxy acids and derivatives | Short-chain hydroxy acids and derivatives |
| Oxindole | C12312 | 59-48-3 | P | C8H7NO | 133.05276 | 134.06016 | 0.77388 | [M+H]+ | 5.621 | 0.94 | 1 | Others | Organoheterocyclic compounds | Indoles and derivatives | Indolines |
| N-Acetylserotonin | C00978 | 1210-83-9 | P | C12H14N2O2 | 218.10553 | 241.09396 | 3.51251 | [M+Na]+ | 5.57 | 0.64 | 1 | Amines | Organoheterocyclic compounds | Indoles and derivatives | Hydroxyindoles |
| L-Tryptophan | C00078 | 73-22-3 | P | C11H12N2O2 | 204.08988 | 205.0974 | 1.07357 | [M+H]+ | 5.233 | 0.81 | 1 | Amino acids, amino acid derivatives | Organoheterocyclic compounds | Indoles and derivatives | Indolyl carboxylic acids and derivatives |
| Indole-3-pyruvic acid | C00331 | 392-12-1 | P | C11H9NO3 | 203.05824 | 226.04702 | 2.06348 | [M+Na]+ | 5.836 | 0.67 | 1 | Organic acids | Organoheterocyclic compounds | Indoles and derivatives | Indolyl carboxylic acids and derivatives |
| Indole-3-carboxaldehyde | C08493 | 487-89-8 | N | C9H7NO | 145.05276 | 144.04474 | 4.97398 | [M-H]- | 5.685 | 0.75 | 1 | Amines | Organoheterocyclic compounds | Indoles and derivatives | Indoles |
| Indole-3-butyric acid | C11284 | 133-32-4 | P | C12H13NO2 | 203.09463 | 226.08417 | 1.67257 | [M+Na]+ | 5.52 | 0.53 | 1 | Organic acids | Organoheterocyclic compounds | Indoles and derivatives | Indoles |
| Indole | C00463 | 120-72-9 | P | C8H7N | 117.05785 | 135.09173 | 0.71912 | [M+NH4]+ | 5.534 | 0.86 | 1 | Amines | Organoheterocyclic compounds | Indoles and derivatives | Indoles |
| 5-Methoxyindoleacetate | C05660 | 3471-31-6 | P | C11H11NO3 | 205.07389 | 228.06347 | 1.86437 | [M+Na]+ | 5.591 | 0.6 | 1 | Organic acids | Organoheterocyclic compounds | Indoles and derivatives | Indolyl carboxylic acids and derivatives |
| Norharmane | C20157 | 244-63-3 | P | C11H8N2 | 168.06875 | 151.06478 | 3.99537 | [M+H-H2O]+ | 0.633 | 0.91 | 2 | Amines | Organoheterocyclic compounds | Indoles and derivatives | Pyridoindoles |

**Table S4** *Cont.*

| name | kegg_id | CAS | IonMode | Formula | MolecularWeight | m/z | Mass Error | Adduct | RT (min) | Score | Level | ClassI | ClassII | ClassIII | ClassⅣ |
| --- | --- | --- | --- | --- | --- | --- | --- | --- | --- | --- | --- | --- | --- | --- | --- |
| N,N-Dihydroxy-L-tryptophan | C19717 | - | P | C11H12N2O4 | 236.07971 | 237.08708 | 0.29974 | [M+H]+ | 4.31 | 0.63 | 2 | Amino acids, amino acid derivatives | Organoheterocyclic compounds | Indoles and derivatives | Indolyl carboxylic acids and derivatives |
| Slaframine | C06185 | - | P | C10H18N2O2 | 198.13683 | 199.14433 | 1.00243 | [M+H]+ | 4.946 | 0.58 | 2 | Amines | Organoheterocyclic compounds | Indolizidines | - |
| isoferulic acid | C10470 | 537-73-5 | P | C10H10O4 | 194.05791 | 217.04733 | 1.10849 | [M+Na]+ | 5.821 | 0.56 | 2 | Organic acids | Phenylpropanoids and polyketides | Cinnamic acids and derivatives | Hydroxycinnamic acids and derivatives |
| Elaeokanine C | C10592 | - | P | C12H21NO2 | 211.15723 | 212.16472 | 0.90161 | [M+H]+ | 5.548 | 0.69 | 2 | Amines | Organoheterocyclic compounds | Indolizidines | - |
| Coumarin | C05851 | 91-64-5 | N | C9H6O2 | 146.03678 | 191.03472 | 1.80893 | [M+HCOOH-H]- | 5.512 | 0.78 | 2 | Others | Phenylpropanoids and polyketides | Coumarins and derivatives | - |
| N-Dimethyl-2-aminoethylphosphonate | C05680 | - | N | C4H12NO3P | 153.05548 | 152.04726 | 6.04138 | [M-H]- | 5.915 | 0.52 | 2 | Amines | Organic acids and derivatives | Organic phosphonic acids and derivatives | Organic phosphonic acids |
| L-CARNITINE | C00318 | 5541-15-1 | P | C7H15NO3 | 161.10519 | 184.09442 | 0.17022 | [M+Na]+ | 5.204 | 0.51 | 2 | Amines | Organic nitrogen compounds | Organonitrogen compounds | Quaternary ammonium salts |
| 4-Methylthiobutanaldoxime | C17241 | - | P | C5H11NOS | 133.05613 | 134.06355 | 0.89726 | [M+H]+ | 5.134 | 0.74 | 2 | Others | Organic nitrogen compounds | Organonitrogen compounds | Oximes |
| 2-epi-5-epi-Valiolone 7-phosphate | C17692 | - | P | C7H13O9P | 272.02972 | 273.03708 | 0.23181 | [M+H]+ | 5.505 | 0.63 | 2 | Alcohols | Organic acids and derivatives | Organic phosphoric acids and derivatives | Phosphate esters |
| trans-urocanate | C00785 | 104-98-3 | P | C6H6N2O2 | 138.04293 | 121.03975 | 0.88328 | [M+H-H2O]+ | 2.305 | 1 | 1 | Organic acids | Organoheterocyclic compounds | Azoles | Imidazoles |
| Terephthalic acid | C06337 | 100-21-0 | N | C8H6O4 | 166.02661 | 165.01876 | 3.31131 | [M-H]- | 5.397 | 0.7 | 1 | Organic acids | Benzenoids | Benzene and substituted derivatives | Benzoic acids and derivatives |
| T-2 Toxin | C09738 | 21259-20-1 | P | C24H34O9 | 466.22028 | 489.20946 | 0.05209 | [M+Na]+ | 5.859 | 0.55 | 1 | Esters | Lipids and lipid-like molecules | Prenol lipids | Sesquiterpenoids |
| Stearamide | C13846 | 124-26-5 | P | C18H37NO | 283.28751 | 284.29461 | 0.70022 | [M+H]+ | 10.333 | 0.8 | 1 | Lipids and lipid like substances | Organic acids and derivatives | Carboximidic acids and derivatives | Carboximidic acids |
| p-Octopamine | C04227 | 104-14-3 | P | C8H11NO2 | 153.07898 | 136.07582 | 0.90617 | [M+H-H2O]+ | 0.853 | 0.81 | 1 | Amines | Benzenoids | Phenols | 1-hydroxy-2-unsubstituted benzenoids |
| Picolinic acid | C10164 | 98-98-6 | P | C6H5NO2 | 123.03203 | 124.03948 | 1.23351 | [M+H]+ | 1.944 | 0.71 | 1 | Organic acids | Organoheterocyclic compounds | Pyridines and derivatives | Pyridinecarboxylic acids and derivatives |
| Phenylethylamine | C05332 | 64-04-0 | P | C8H11N | 121.08915 | 122.09668 | 1.91166 | [M+H]+ | 5.148 | 0.67 | 1 | Amines | Benzenoids | Benzene and substituted derivatives | Phenethylamines |
| Palmitoylethanolamide | C16512 | 544-31-0 | P | C18H37NO2 | 299.28243 | 282.27924 | 0.36454 | [M+H-H2O]+ | 7.792 | 0.78 | 1 | Lipids and lipid like substances | Organic acids and derivatives | Carboximidic acids and derivatives | Carboximidic acids |
| Nudifloramide | C05842 | 701-44-0 | P | C7H8N2O2 | 152.05858 | 135.05542 | 0.89506 | [M+H-H2O]+ | 5.304 | 0.61 | 1 | Amines | Organoheterocyclic compounds | Pyridines and derivatives | Pyridinecarboxylic acids and derivatives |
| Nicotinamide | C00153 | 98-92-0 | P | C6H6N2O | 122.04801 | 123.05549 | 1.44143 | [M+H]+ | 2.016 | 0.81 | 1 | Amines | Organoheterocyclic compounds | Pyridines and derivatives | Pyridinecarboxylic acids and derivatives |

**Table S4** *Cont.*

| name | kegg_id | CAS | IonMode | Formula | MolecularWeight | m/z | Mass Error | Adduct | RT (min) | Score | Level | ClassI | ClassII | ClassIII | ClassⅣ |
| --- | --- | --- | --- | --- | --- | --- | --- | --- | --- | --- | --- | --- | --- | --- | --- |
| Methyl p-tert-butylphenylacetate | NA | 3549-23-3 | P | C13H18O2 | 206.13068 | 229.12022 | 1.6384 | [M+Na]+ | 5.821 | 0.59 | 1 | Esters | Benzenoids | Benzene and substituted derivatives | Phenylpropanes |
| Linoleic acid | C01595 | 60-33-3 | P | C18H32O2 | 280.24023 | 263.23698 | 0.16211 | [M+H-H2O]+ | 8.682 | 0.91 | 1 | Organic acids | Lipids and lipid-like molecules | Fatty Acyls | Lineolic acids and derivatives |
| Kaurenoic acid | C11874 | 6730-83-2 | N | C20H30O2 | 302.22458 | 301.21744 | 0.51943 | [M-H]- | 9.624 | 0.7 | 1 | Organic acids | Lipids and lipid-like molecules | Prenol lipids | Diterpenoids |
| Isopimaric acid | C09118 | 5835-26-7 | P | C20H30O2 | 302.22458 | 285.22119 | 0.31072 | [M+H-H2O]+ | 9.63 | 0.59 | 1 | Organic acids | Lipids and lipid-like molecules | Prenol lipids | Diterpenoids |
| Geraniol | C01500 | 106-24-1 | P | C10H18O | 154.13576 | 137.13256 | 0.67089 | [M+H-H2O]+ | 5.893 | 0.71 | 1 | Alcohols | Lipids and lipid-like molecules | Prenol lipids | Monoterpenoids |
| Farnesol | C01126 | 4602-84-0 | P | C15H26O | 222.19836 | 245.1877 | 0.61079 | [M+Na]+ | 8.428 | 0.54 | 1 | Alcohols | Lipids and lipid-like molecules | Prenol lipids | Sesquiterpenoids |
| D-Glucurono-6,3-lactone | C02670 | 32449-92-6 | P | C6H8O6 | 176.03209 | 199.02148 | 1.05986 | [M+Na]+ | 2.738 | 0.54 | 1 | Sugar derivative | Organoheterocyclic compounds | Furofurans | Isosorbides |
| D-(+)-Phenyllactic acid | C05607 | 7326-19-4 | N | C9H10O3 | 166.063 | 165.05513 | 3.40672 | [M-H]- | 5.656 | 0.73 | 1 | Organic acids | Phenylpropanoids and polyketides | Phenylpropanoic acids | - |
| Beta-Zearalanol | C14753 | 42422-68-4 | P | C18H26O5 | 322.17802 | 345.16758 | 1.10962 | [M+Na]+ | 6.168 | 0.56 | 1 | Alcohols | Phenylpropanoids and polyketides | Macrolides and analogues | - |
| beta-D-Glucopyranosyl abscisate | C15970 | 21414-42-6 | P | C21H30O9 | 426.18899 | 449.17848 | 0.67233 | [M+Na]+ | 5.52 | 0.63 | 1 | Sugar derivative | Lipids and lipid-like molecules | Prenol lipids | Sesquiterpenoids |
| Benzamide | C09815 | 55-21-0 | P | C7H7NO | 121.05276 | 122.0603 | 1.97741 | [M+H]+ | 5.347 | 0.64 | 1 | Amines | Benzenoids | Benzene and substituted derivatives | Benzoic acids and derivatives |
| 4-Hydroxyphenylpyruvic acid | C01179 | 156-39-8 | P | C9H8O4 | 180.04226 | 163.03847 | 2.72622 | [M+H-H2O]+ | 4.7 | 0.87 | 1 | Organic acids | Benzenoids | Benzene and substituted derivatives | Phenylpyruvic acid derivatives |
| 4-Hydroxybenzaldehyde | C00633 | 123-08-0 | N | C7H6O2 | 122.03678 | 121.02861 | 7.13817 | [M-H]- | 5.455 | 1 | 1 | Others | Organic oxygen compounds | Organooxygen compounds | Carbonyl compounds |
| 4-Ethylphenol | C13637 | 123-07-9 | P | C8H10O | 122.07317 | 123.08067 | 1.67897 | [M+H]+ | 5.606 | 0.79 | 1 | Others | Benzenoids | Phenols | 1-hydroxy-2-unsubstituted benzenoids |
| 4-(1,2-Dihydroxyethyl)benzene-1,2-diol | C05576 | 28822-73-3 | P | C8H10O4 | 170.05791 | 193.04807 | 5.66806 | [M+Na]+ | 3.712 | 0.51 | 1 | Alcohols | Benzenoids | Phenols | Benzenediols |
| 3-Hydroxybenzoic acid | C00587 | 36320 | N | C7H6O3 | 138.0317 | 137.02347 | 6.76193 | [M-H]- | 5.915 | 0.8 | 1 | Organic acids | Benzenoids | Benzene and substituted derivatives | Benzoic acids and derivatives |
| 2-Phenylacetamide | C02505 | 103-81-1 | P | C8H9NO | 135.06841 | 136.07579 | 0.59035 | [M+H]+ | 5.448 | 0.86 | 1 | Amines | Benzenoids | Benzene and substituted derivatives | Phenylacetamides |

**Table S4** *Cont.*

| name | kegg_id | CAS | IonMode | Formula | MolecularWeight | m/z | Mass Error | Adduct | RT (min) | Score | Level | ClassI | ClassII | ClassIII | ClassⅣ |
| --- | --- | --- | --- | --- | --- | --- | --- | --- | --- | --- | --- | --- | --- | --- | --- |
| 2-Hydroxymethyl benzoic acid | C10804 | 612-20-4 | P | C8H8O3 | 152.04735 | 135.0442 | 0.98574 | [M+H-H2O]+ | 5.563 | 0.79 | 1 | Organic acids | Benzenoids | Benzene and substituted derivatives | Benzoic acids and derivatives |
| Victoxinine | NA | - | P | C17H29NO | 263.22491 | 264.23244 | 0.88754 | [M+H]+ | 5.937 | 0.64 | 2 | Amines | Lipids and lipid-like molecules | Prenol lipids | Sesquiterpenoids |
| Tetrahydrocytisine | NA | - | P | C11H18N2O | 194.14191 | 195.14927 | 0.32867 | [M+H]+ | 6.212 | 0.63 | 2 | Amines | Organoheterocyclic compounds | Quinolizidines | Quinolizidinones |
| Styrene | C07083 | 100-42-5 | P | C8H8 | 104.0626 | 105.07028 | 3.60963 | [M+H]+ | 5.148 | 0.94 | 2 | Others | Benzenoids | Benzene and substituted derivatives | Styrenes |
| Strongylophorin 22 | NA | - | N | C26H38O2 | 382.28718 | 381.2801 | 0.5818 | [M-H]- | 9.836 | 0.58 | 2 | Steroid hormones, steroids | Organoheterocyclic compounds | Benzopyrans | 1-benzopyrans |
| Sapropterin | C00272 | 69056-38-8 | P | C9H15N5O3 | 241.11749 | 242.12484 | 0.21329 | [M+H]+ | 4.571 | 0.51 | 2 | Amines | Organoheterocyclic compounds | Pteridines and derivatives | Pterins and derivatives |
| Quinolactacin A | NA | - | P | C16H18N2O2 | 270.13683 | 271.14413 | 0.01254 | [M+H]+ | 6.429 | 0.54 | 2 | Amines | Organoheterocyclic compounds | Quinolines and derivatives | Pyrroloquinolines |
| P-Toluenesulfonic acid | C06677 | 104-15-4 | N | C7H8O3S | 172.01942 | 171.01151 | 3.56269 | [M-H]- | 5.211 | 1 | 2 | Organic acids | Benzenoids | Benzene and substituted derivatives | Benzenesulfonic acids and derivatives |
| Plakortic acid | C17158 | - | P | C17H30O4 | 298.21441 | 299.22154 | 0.58101 | [M+H]+ | 7.841 | 0.64 | 2 | Organic acids | Organoheterocyclic compounds | Dioxanes | 1,2-dioxanes |
| Pandangolide 1 | NA | - | N | C12H20O5 | 244.13108 | 243.12404 | 1.05635 | [M-H]- | 5.742 | 0.71 | 2 | Alcohols | Phenylpropanoids and polyketides | Macrolides and analogues | - |
| nigerapyrone A | NA | - | P | C20H18O3 | 306.12559 | 307.13388 | 3.24204 | [M+H]+ | 6.983 | 0.57 | 2 | Esters | Benzenoids | Benzene and substituted derivatives | Biphenyls and derivatives |
| Neopterin | C05926 | - | N | C9H11N5O4 | 253.08111 | 252.07366 | 0.59656 | [M-H]- | 6.074 | 0.85 | 2 | Amines | Organoheterocyclic compounds | Pteridines and derivatives | Pterins and derivatives |
| Naphthalene-2-sulfonic acid | C16202 | 120-18-3 | N | C10H8O3S | 208.01942 | 207.01203 | 0.43501 | [M-H]- | 5.483 | 0.58 | 2 | Organic acids | Benzenoids | Naphthalenes | Naphthalene sulfonic acids and derivatives |
| Myxopyronin A | NA | - | P | C23H31NO6 | 417.21514 | 418.22214 | 0.7271 | [M+H]+ | 6.371 | 0.84 | 2 | Amines | Organic oxygen compounds | Organooxygen compounds | Carbonyl compounds |
| Monanchorin | NA | - | P | C11H21N3O | 211.16846 | 212.17612 | 1.69103 | [M+H]+ | 5.735 | 0.54 | 2 | Amines | Organoheterocyclic compounds | Diazepines | 1,3-diazepines |
| Lophophorine | C09573 | - | P | C13H17NO3 | 235.12084 | 236.12828 | 0.58072 | [M+H]+ | 4.77 | 0.59 | 2 | Amines | Organoheterocyclic compounds | Tetrahydroisoquinolines | - |
| Loliolide | NA | - | P | C11H16O3 | 196.10995 | 197.11752 | 1.38018 | [M+H]+ | 5.635 | 0.97 | 2 | Esters | Organoheterocyclic compounds | Benzofurans | - |
| Ketoleucine | C00233 | 816-66-0 | P | C6H10O3 | 130.063 | 131.07046 | 1.21023 | [M+H]+ | 0.647 | 0.63 | 2 | Amino acids, amino acid derivatives | Organic acids and derivatives | Keto acids and derivatives | Short-chain keto acids and derivatives |
| Indolo[2,1-b]quinazoline-6,12-dione | C10742 | - | P | C15H8N2O2 | 248.05858 | 249.065 | 3.52904 | [M+H]+ | 5.635 | 0.57 | 2 | Amines | Organoheterocyclic compounds | Diazanaphthalenes | Benzodiazines |

**Table S4** *Cont.*

| name | kegg_id | CAS | IonMode | Formula | MolecularWeight | m/z | Mass Error | Adduct | RT (min) | Score | Level | ClassI | ClassII | ClassIII | ClassⅣ |
| --- | --- | --- | --- | --- | --- | --- | --- | --- | --- | --- | --- | --- | --- | --- | --- |
| Hyrtioerectine B | NA | - | P | C13H14N2O3 | 246.10044 | 247.108 | 1.06225 | [M+H]+ | 5.276 | 0.63 | 2 | Amines | Alkaloids and derivatives | Harmala alkaloids | - |
| Hymenoflorin | C09480 | - | P | C15H20O5 | 280.13108 | 281.13831 | 0.25277 | [M+H]+ | 6.689 | 0.9 | 2 | Esters | Lipids and lipid-like molecules | Prenol lipids | Terpene lactones |
| Gliocladic acid_130132 | NA | - | P | C14H22O4 | 254.15181 | 277.14136 | 1.37295 | [M+Na]+ | 5.994 | 0.57 | 2 | Organic acids | Lipids and lipid-like molecules | Prenol lipids | Monoterpenoids |
| Gibberellin A63 | NA | 63351-80-4 | P | C19H24O6 | 348.15729 | 349.16509 | 1.44149 | [M+H]+ | 5.435 | 0.87 | 2 | Organic acids | Lipids and lipid-like molecules | Prenol lipids | Diterpenoids |
| Gibberellin A52 | NA | 68062-24-8 | P | C20H26O7 | 378.16785 | 379.17584 | 1.82402 | [M+H]+ | 5.462 | 0.79 | 2 | Organic acids | Lipids and lipid-like molecules | Prenol lipids | Diterpenoids |
| Fiscalin C | NA | - | P | C27H29N5O4 | 487.22196 | 488.22925 | 0.01112 | [M+H]+ | 6.328 | 0.68 | 2 | Amines | Organoheterocyclic compounds | Diazanaphthalenes | Benzodiazines |
| Drosopterin | NA | - | P | C15H16N10O2 | 368.14577 | 369.15215 | 2.49003 | [M+H]+ | 6.633 | 0.65 | 2 | Amines | Organoheterocyclic compounds | Pteridines and derivatives | Pterins and derivatives |
| Dinocton 6 | C18922 | - | P | C16H22N2O7 | 354.1427 | 355.14964 | 1.0054 | [M+H]+ | 6.575 | 0.67 | 2 | Esters | Benzenoids | Benzene and substituted derivatives | Nitrobenzenes |
| destruxin A | NA | - | P | C29H47N5O7 | 577.34755 | 600.33685 | 0.17421 | [M+Na]+ | 6.255 | 0.55 | 2 | Amines | Organic acids and derivatives | Peptidomimetics | Depsipeptides |
| Dehydroascorbic acid | C05422 | 490-83-5 | P | C6H6O6 | 174.01644 | 175.02294 | 4.57931 | [M+H]+ | 2.506 | 0.61 | 2 | Organic acids | Organoheterocyclic compounds | Lactones | Gamma butyrolactones |
| cis-2-Hydroxymethyl-3-methylcyclopentanone | NA | - | P | C7H12O2 | 128.08373 | 129.09115 | 0.91526 | [M+H]+ | 0.676 | 0.62 | 2 | Alcohols | Organic oxygen compounds | Organooxygen compounds | Carbonyl compounds |
| Chloriolide | NA | - | N | C12H18O4 | 226.12051 | 225.1133 | 0.41612 | [M-H]- | 5.699 | 0.65 | 2 | Esters | Phenylpropanoids and polyketides | Macrolides and analogues | - |
| Aigialone | NA | - | P | C16H26O5 | 298.17802 | 299.18577 | 1.51422 | [M+H]+ | 8.099 | 0.64 | 2 | Others | Organoheterocyclic compounds | Furopyrans | - |
| 9(S)-HpOTrE | C16321 | 111004-08-1 | N | C18H30O4 | 310.21441 | 309.20741 | 0.97119 | [M-H]- | 7.064 | 0.67 | 2 | Organic acids | Lipids and lipid-like molecules | Fatty Acyls | Lineolic acids and derivatives |
| 9(S)-HOTrE | C16326 | 89886-42-0 | P | C18H30O3 | 294.21949 | 277.21619 | 0.00222 | [M+H-H2O]+ | 6.757 | 0.85 | 2 | Organic acids | Lipids and lipid-like molecules | Fatty Acyls | Lineolic acids and derivatives |
| 4-Nitrophenol | C00870 | 100-02-7 | N | C6H5NO3 | 139.02694 | 138.01889 | 5.40405 | [M-H]- | 5.786 | 0.82 | 2 | Others | Benzenoids | Phenols | Nitrophenols |
| 4-Isopropylbenzyl alcohol | C06576 | 536-60-7 | P | C10H14O | 150.10447 | 133.10131 | 0.93202 | [M+H-H2O]+ | 5.865 | 0.77 | 2 | Alcohols | Lipids and lipid-like molecules | Prenol lipids | Monoterpenoids |
| 3-Methylsalicylic acid | C14088 | 83-40-9 | P | C8H8O3 | 152.04735 | 153.05507 | 2.75528 | [M+H]+ | 0.647 | 0.84 | 2 | Organic acids | Benzenoids | Benzene and substituted derivatives | Benzoic acids and derivatives |
| 3-Methyl-2-oxovaleric acid | C00671 | 1460-34-0 | P | C6H10O3 | 130.063 | 153.05259 | 2.96662 | [M+Na]+ | 1.9 | 0.78 | 2 | Organic acids | Organic acids and derivatives | Keto acids and derivatives | Short-chain keto acids and derivatives |

**Table S4** *Cont.*

| name | kegg_id | CAS | IonMode | Formula | MolecularWeight | m/z | Mass Error | Adduct | RT (min) | Score | Level | ClassI | ClassII | ClassIII | ClassⅣ |
| --- | --- | --- | --- | --- | --- | --- | --- | --- | --- | --- | --- | --- | --- | --- | --- |
| 3,4-Dihydroxyhydrocinnamic acid | C10447 | 1078-61-1 | P | C9H10O4 | 182.05791 | 165.05392 | 3.80632 | [M+H-H2O]+ | 1.188 | 0.86 | 2 | Organic acids | Phenylpropanoids and polyketides | Phenylpropanoic acids | - |
| 2-Hydroxycampholonic acid | NA | - | P | C10H16O4 | 200.10486 | 201.11217 | 0.0514 | [M+H]+ | 6.27 | 0.54 | 2 | Organic acids | Lipids and lipid-like molecules | Prenol lipids | Monoterpenoids |
| 2-Aminophenoxazin-3-one | C02161 | - | P | C12H8N2O2 | 212.05858 | 213.06628 | 1.8673 | [M+H]+ | 5.491 | 0.52 | 2 | Amines | Organoheterocyclic compounds | Benzoxazines | Phenoxazines |
| 1-METHYL-HYDANTOIN | NA | - | P | C4H6N2O2 | 114.04293 | 115.05051 | 2.47583 | [M+H]+ | 2.16 | 0.56 | 2 | Amines | Organoheterocyclic compounds | Azolines | Imidazolines |
| 1-Methoxyphenanthrene | C11433 | 834-99-1 | P | C15H12O | 208.08881 | 209.09532 | 3.78344 | [M+H]+ | 6.487 | 0.64 | 2 | Others | Benzenoids | Phenanthrenes and derivatives | - |
| 1-Hydroxy-2-naphthoate | C03203 | 86-48-6 | P | C11H8O3 | 188.04735 | 171.04305 | 5.31609 | [M+H-H2O]+ | 1.188 | 0.84 | 2 | Organic acids | Benzenoids | Naphthalenes | Naphthalenecarboxylic acids and derivatives |
| 13-OxoODE | C14765 | - | P | C18H30O3 | 294.21949 | 317.20873 | 0.1202 | [M+Na]+ | 7.725 | 0.86 | 2 | Organic acids | Lipids and lipid-like molecules | Fatty Acyls | Lineolic acids and derivatives |
| 1,8-Diazacyclotetradecane-2,9-dione | C04277 | - | P | C12H22N2O2 | 226.16813 | 227.17559 | 0.72663 | [M+H]+ | 5.247 | 0.73 | 2 | Amines | Phenylpropanoids and polyketides | Macrolactams | - |
| (ent-6alpha,7alpha)-6,7-Dihydroxy-16-kauren-19-oic acid | C11876 | 26109-32-0 | N | C20H30O4 | 334.21441 | 333.20751 | 1.1828 | [M-H]- | 6.743 | 0.57 | 2 | Organic acids | Lipids and lipid-like molecules | Prenol lipids | Diterpenoids |
| (1R,6R)-6-Hydroxy-2-succinylcyclohexa-2,4-diene-1-carboxylate | C05817 | - | P | C11H12O6 | 240.06339 | 241.07073 | 0.1463 | [M+H]+ | 6.067 | 0.63 | 2 | Organic acids | Organic acids and derivatives | Keto acids and derivatives | Gamma-keto acids and derivatives |

**Note:** P: Positive ion mode; N: Negative ion mode.

Table S5 Information on OTU abundance of marine benthic fungi

| OTU ID | A1-1 | A1-2 | A1-3 | B3-1 | B3-2 | B3-3 | C2-1 | C2-2 | C2-3 | C3-1 | C3-2 | C3-3 | M0-1 | M0-2 | M0-3 |
| --- | --- | --- | --- | --- | --- | --- | --- | --- | --- | --- | --- | --- | --- | --- | --- |
| OTU000001 | 1650 | 8155 | 9261 | 73336 | 75236 | 54519 | 340 | 317 | 359 | 832 | 290 | 2754 | 29287 | 19204 | 413 |
| OTU000003 | 6 | 33620 | 0 | 1063 | 844 | 1264 | 0 | 6 | 8 | 2 | 0 | 0 | 15259 | 5752 | 0 |
| OTU000004 | 1580 | 4123 | 452 | 551 | 219 | 76 | 8046 | 7378 | 4599 | 6801 | 84 | 567 | 2162 | 69 | 4164 |
| OTU000005 | 291 | 2 | 236 | 420 | 0 | 266 | 6033 | 8015 | 2280 | 4177 | 2125 | 2739 | 4 | 0 | 238 |
| OTU000007 | 1 | 13557 | 83 | 263 | 198 | 153 | 0 | 0 | 2 | 1 | 0 | 0 | 5989 | 4354 | 0 |
| OTU000010 | 364 | 315 | 5727 | 257 | 76 | 6 | 1797 | 1847 | 973 | 1475 | 50 | 68 | 6 | 3601 | 37 |
| OTU000011 | 1 | 1670 | 10791 | 149 | 23 | 22 | 15 | 0 | 0 | 0 | 0 | 0 | 40 | 25 | 0 |
| OTU000012 | 2 | 4308 | 0 | 78 | 99 | 78 | 0 | 0 | 2 | 1 | 0 | 0 | 5302 | 2038 | 0 |
| OTU000013 | 31 | 570 | 1738 | 2315 | 1423 | 811 | 44 | 48 | 35 | 20 | 22 | 25 | 707 | 3775 | 44 |
| OTU000014 | 17 | 106 | 4254 | 1877 | 497 | 1082 | 16 | 19 | 24 | 28 | 26 | 3 | 1216 | 1009 | 48 |
| OTU000015 | 3282 | 1065 | 171 | 19 | 119 | 14 | 64 | 28 | 50 | 2622 | 624 | 112 | 953 | 18 | 2 |
| OTU000016 | 1 | 3200 | 0 | 94 | 106 | 97 | 0 | 0 | 0 | 0 | 0 | 0 | 5356 | 39 | 0 |
| OTU000017 | 348 | 24 | 147 | 259 | 7 | 1 | 3661 | 2078 | 1797 | 4 | 4 | 5 | 4 | 289 | 22 |
| OTU000018 | 2 | 4514 | 0 | 106 | 128 | 84 | 0 | 0 | 0 | 0 | 0 | 0 | 3701 | 72 | 0 |
| OTU000019 | 479 | 0 | 0 | 0 | 0 | 0 | 4182 | 180 | 2994 | 11 | 3 | 3 | 0 | 0 | 1 |
| OTU000020 | 1425 | 591 | 0 | 46 | 18 | 20 | 3311 | 57 | 74 | 1549 | 20 | 41 | 681 | 15 | 3 |
| OTU000021 | 1310 | 2166 | 0 | 30 | 130 | 19 | 1461 | 27 | 27 | 141 | 803 | 806 | 36 | 594 | 8 |
| OTU000022 | 257 | 13 | 0 | 4 | 13 | 3 | 3340 | 55 | 938 | 1178 | 9 | 107 | 603 | 0 | 868 |
| OTU000023 | 1037 | 490 | 3453 | 10 | 131 | 7 | 22 | 10 | 13 | 2045 | 16 | 50 | 8 | 16 | 4 |
| OTU000024 | 240 | 47 | 3 | 64 | 1743 | 19 | 2393 | 1230 | 437 | 646 | 4 | 21 | 25 | 0 | 1 |
| OTU000025 | 216 | 2 | 338 | 1 | 125 | 1 | 1213 | 4393 | 555 | 8 | 5 | 11 | 1 | 0 | 2 |
| OTU000027 | 0 | 2712 | 0 | 136 | 135 | 97 | 0 | 0 | 0 | 0 | 0 | 0 | 3588 | 36 | 0 |
| OTU000028 | 235 | 0 | 0 | 18 | 1 | 0 | 2141 | 1235 | 784 | 1055 | 632 | 87 | 0 | 0 | 3 |
| OTU000029 | 0 | 2501 | 0 | 52 | 48 | 54 | 0 | 0 | 0 | 0 | 0 | 0 | 2043 | 1409 | 0 |
| OTU000030 | 2317 | 0 | 0 | 0 | 0 | 0 | 3 | 12 | 876 | 2652 | 2 | 11 | 0 | 0 | 0 |
| OTU000031 | 51 | 0 | 0 | 12 | 37 | 0 | 929 | 10 | 481 | 96 | 620 | 1984 | 2 | 0 | 1505 |
| OTU000032 | 1808 | 2 | 0 | 0 | 0 | 0 | 57 | 42 | 919 | 203 | 31 | 2347 | 0 | 0 | 22 |
| OTU000033 | 248 | 0 | 0 | 0 | 0 | 0 | 176 | 3934 | 833 | 5 | 3 | 2 | 0 | 0 | 0 |
| OTU000034 | 148 | 288 | 679 | 4 | 4 | 1 | 3461 | 299 | 96 | 12 | 8 | 10 | 4 | 0 | 5 |
| OTU000035 | 0 | 2405 | 0 | 43 | 48 | 37 | 0 | 0 | 0 | 0 | 0 | 0 | 1696 | 741 | 0 |
| OTU000036 | 2 | 2910 | 0 | 77 | 49 | 26 | 0 | 0 | 0 | 0 | 0 | 0 | 1201 | 623 | 0 |
| OTU000037 | 6 | 0 | 2117 | 0 | 0 | 0 | 6 | 1 | 3 | 157 | 46 | 2409 | 0 | 0 | 37 |
| OTU000038 | 692 | 153 | 0 | 6 | 175 | 1 | 16 | 5 | 11 | 3503 | 48 | 90 | 2 | 3 | 3 |
| OTU000039 | 1 | 2167 | 0 | 123 | 57 | 37 | 1 | 0 | 0 | 4 | 1 | 68 | 1415 | 52 | 628 |
| OTU000040 | 1401 | 0 | 0 | 0 | 0 | 0 | 23 | 14 | 23 | 2912 | 23 | 60 | 0 | 0 | 0 |
| OTU000041 | 229 | 105 | 373 | 32 | 14 | 4 | 146 | 521 | 1610 | 1021 | 30 | 64 | 280 | 0 | 14 |

**Table S5** *Cont.*

| OTU ID | A1-1 | A1-2 | A1-3 | B3-1 | B3-2 | B3-3 | C2-1 | C2-2 | C2-3 | C3-1 | C3-2 | C3-3 | M0-1 | M0-2 | M0-3 |
| --- | --- | --- | --- | --- | --- | --- | --- | --- | --- | --- | --- | --- | --- | --- | --- |
| OTU000042 | 1474 | 0 | 0 | 4 | 129 | 0 | 22 | 10 | 13 | 892 | 495 | 1177 | 2 | 0 | 9 |
| OTU000044 | 60 | 0 | 0 | 0 | 0 | 0 | 530 | 42 | 189 | 171 | 52 | 3094 | 0 | 0 | 40 |
| OTU000045 | 174 | 0 | 113 | 0 | 0 | 0 | 1050 | 866 | 1947 | 1 | 0 | 5 | 0 | 0 | 0 |
| OTU000046 | 2198 | 0 | 536 | 0 | 0 | 0 | 81 | 26 | 48 | 210 | 25 | 769 | 0 | 0 | 11 |
| OTU000047 | 896 | 0 | 0 | 0 | 0 | 0 | 18 | 15 | 17 | 163 | 34 | 2553 | 0 | 0 | 37 |
| OTU000049 | 946 | 0 | 0 | 0 | 0 | 0 | 15 | 8 | 19 | 111 | 15 | 2507 | 0 | 0 | 34 |
| OTU000051 | 106 | 0 | 0 | 0 | 0 | 0 | 1 | 0 | 0 | 3072 | 7 | 5 | 0 | 0 | 0 |
| OTU000054 | 2157 | 86 | 0 | 3 | 0 | 0 | 27 | 15 | 21 | 89 | 6 | 35 | 3 | 2 | 715 |
| OTU000055 | 91 | 0 | 0 | 0 | 0 | 0 | 2911 | 22 | 52 | 2 | 0 | 2 | 0 | 0 | 0 |
| OTU000056 | 105 | 233 | 102 | 51 | 3 | 3 | 0 | 2 | 1 | 2477 | 32 | 58 | 2 | 6 | 0 |
| OTU000057 | 174 | 0 | 0 | 0 | 0 | 0 | 467 | 1554 | 430 | 43 | 8 | 388 | 0 | 0 | 10 |
| OTU000058 | 184 | 0 | 11 | 0 | 0 | 0 | 1022 | 1678 | 89 | 6 | 2 | 5 | 0 | 0 | 1 |
| OTU000059 | 0 | 0 | 2985 | 0 | 0 | 0 | 0 | 0 | 0 | 0 | 0 | 0 | 0 | 0 | 0 |
| OTU000060 | 0 | 82 | 0 | 2 | 0 | 0 | 5 | 2 | 0 | 84 | 28 | 2736 | 1 | 3 | 40 |
| OTU000061 | 0 | 1253 | 0 | 46 | 46 | 22 | 0 | 0 | 0 | 0 | 0 | 0 | 1597 | 11 | 0 |
| OTU000062 | 2559 | 0 | 0 | 0 | 0 | 0 | 63 | 55 | 75 | 173 | 8 | 2 | 0 | 0 | 1 |
| OTU000063 | 34 | 11 | 0 | 66 | 362 | 15 | 26 | 6 | 683 | 25 | 12 | 809 | 10 | 0 | 810 |
| OTU000064 | 1 | 0 | 0 | 0 | 0 | 0 | 5 | 2 | 1 | 69 | 9 | 2725 | 0 | 0 | 48 |
| OTU000065 | 3 | 0 | 0 | 0 | 0 | 0 | 0 | 5 | 2 | 21 | 2794 | 15 | 0 | 0 | 0 |
| OTU000066 | 3 | 1 | 0 | 5 | 211 | 1 | 0 | 1 | 0 | 70 | 2408 | 53 | 7 | 0 | 67 |
| OTU000067 | 2536 | 0 | 1 | 0 | 0 | 0 | 75 | 35 | 44 | 103 | 7 | 0 | 0 | 0 | 1 |
| OTU000068 | 871 | 0 | 0 | 0 | 0 | 0 | 7 | 1 | 16 | 40 | 0 | 101 | 0 | 0 | 1754 |
| OTU000069 | 0 | 224 | 0 | 37 | 22 | 20 | 0 | 3 | 0 | 0 | 1 | 0 | 1744 | 725 | 0 |
| OTU000070 | 211 | 0 | 0 | 0 | 0 | 0 | 5 | 3 | 1 | 2340 | 35 | 85 | 0 | 0 | 1 |
| OTU000071 | 105 | 0 | 0 | 0 | 0 | 0 | 2489 | 28 | 47 | 3 | 1 | 0 | 0 | 0 | 1 |
| OTU000072 | 2329 | 0 | 0 | 0 | 0 | 0 | 72 | 34 | 58 | 155 | 0 | 5 | 0 | 0 | 0 |
| OTU000073 | 0 | 0 | 0 | 0 | 0 | 0 | 0 | 1 | 0 | 69 | 11 | 2525 | 0 | 0 | 40 |
| OTU000074 | 2416 | 1 | 0 | 0 | 0 | 0 | 29 | 11 | 21 | 133 | 3 | 0 | 0 | 0 | 1 |
| OTU000075 | 49 | 190 | 190 | 1 | 1 | 2 | 1317 | 18 | 641 | 165 | 0 | 8 | 0 | 4 | 0 |
| OTU000076 | 38 | 66 | 0 | 46 | 33 | 3 | 48 | 372 | 1460 | 0 | 0 | 1 | 7 | 485 | 0 |
| OTU000077 | 3 | 0 | 0 | 0 | 0 | 0 | 1 | 1 | 2 | 93 | 29 | 2394 | 0 | 0 | 34 |
| OTU000078 | 2 | 0 | 0 | 0 | 0 | 0 | 2 | 0 | 2 | 83 | 19 | 2398 | 0 | 0 | 41 |
| OTU000079 | 1 | 2238 | 0 | 15 | 29 | 173 | 0 | 0 | 0 | 0 | 0 | 0 | 42 | 48 | 0 |
| OTU000080 | 125 | 118 | 0 | 5 | 2 | 1 | 26 | 1 | 4 | 1743 | 37 | 433 | 2 | 3 | 7 |
| OTU000081 | 2265 | 0 | 0 | 0 | 0 | 0 | 54 | 28 | 36 | 109 | 0 | 2 | 0 | 0 | 0 |
| OTU000082 | 2 | 0 | 0 | 0 | 0 | 0 | 0 | 3 | 0 | 45 | 14 | 2403 | 0 | 0 | 26 |
| OTU000083 | 16 | 0 | 0 | 0 | 0 | 0 | 23 | 10 | 13 | 83 | 23 | 2284 | 0 | 0 | 28 |

**Table S5** *Cont.*

| OTU ID | A1-1 | A1-2 | A1-3 | B3-1 | B3-2 | B3-3 | C2-1 | C2-2 | C2-3 | C3-1 | C3-2 | C3-3 | M0-1 | M0-2 | M0-3 |
| --- | --- | --- | --- | --- | --- | --- | --- | --- | --- | --- | --- | --- | --- | --- | --- |
| OTU000085 | 1 | 0 | 0 | 0 | 0 | 0 | 1 | 3 | 0 | 57 | 19 | 2277 | 0 | 0 | 36 |
| OTU000086 | 0 | 60 | 0 | 1 | 0 | 0 | 0 | 0 | 0 | 3 | 0 | 20 | 0 | 1156 | 1146 |
| OTU000087 | 2147 | 0 | 0 | 0 | 0 | 0 | 37 | 20 | 24 | 130 | 0 | 1 | 0 | 0 | 7 |
| OTU000088 | 1 | 0 | 893 | 0 | 0 | 0 | 2 | 0 | 1 | 92 | 1286 | 47 | 0 | 0 | 1 |
| OTU000089 | 55 | 0 | 0 | 0 | 0 | 0 | 40 | 26 | 2113 | 2 | 2 | 1 | 0 | 0 | 0 |
| OTU000090 | 0 | 0 | 0 | 0 | 0 | 0 | 1 | 1 | 1 | 84 | 28 | 2082 | 0 | 0 | 41 |
| OTU000091 | 0 | 0 | 0 | 0 | 0 | 0 | 0 | 0 | 0 | 59 | 498 | 1645 | 0 | 0 | 14 |
| OTU000092 | 0 | 0 | 35 | 0 | 0 | 0 | 1 | 0 | 0 | 8 | 2 | 2129 | 0 | 0 | 21 |
| OTU000093 | 1 | 511 | 0 | 11 | 26 | 14 | 0 | 0 | 0 | 37 | 776 | 23 | 746 | 3 | 1 |
| OTU000094 | 0 | 0 | 2128 | 0 | 0 | 0 | 0 | 0 | 0 | 0 | 0 | 0 | 0 | 0 | 0 |
| OTU000095 | 0 | 1895 | 1 | 69 | 38 | 33 | 1 | 0 | 0 | 0 | 0 | 0 | 32 | 28 | 0 |
| OTU000097 | 112 | 0 | 0 | 0 | 0 | 0 | 0 | 0 | 0 | 1882 | 24 | 47 | 0 | 0 | 0 |
| OTU000100 | 0 | 261 | 0 | 16 | 267 | 1 | 0 | 0 | 0 | 1 | 0 | 0 | 4 | 1502 | 0 |
| OTU000101 | 4 | 0 | 0 | 0 | 0 | 0 | 0 | 3 | 3 | 66 | 1915 | 40 | 0 | 0 | 0 |
| OTU000103 | 3 | 0 | 0 | 0 | 0 | 0 | 2 | 0 | 0 | 141 | 22 | 1808 | 0 | 0 | 25 |
| OTU000104 | 17 | 0 | 0 | 0 | 0 | 0 | 7 | 5 | 236 | 83 | 23 | 1592 | 0 | 0 | 16 |
| OTU000105 | 4 | 0 | 0 | 0 | 0 | 0 | 1 | 5 | 1 | 70 | 1845 | 48 | 0 | 0 | 2 |
| OTU000106 | 1765 | 0 | 0 | 0 | 0 | 0 | 38 | 16 | 40 | 102 | 0 | 4 | 0 | 0 | 0 |
| OTU000107 | 46 | 0 | 0 | 0 | 0 | 0 | 709 | 1173 | 29 | 0 | 1 | 2 | 0 | 0 | 0 |
| OTU000108 | 0 | 0 | 1946 | 0 | 0 | 0 | 0 | 1 | 3 | 5 | 0 | 0 | 0 | 0 | 1 |
| OTU000109 | 140 | 0 | 0 | 0 | 0 | 0 | 1 | 0 | 2 | 1721 | 11 | 38 | 0 | 0 | 6 |
| OTU000110 | 45 | 0 | 0 | 0 | 0 | 0 | 1600 | 30 | 224 | 4 | 0 | 3 | 0 | 0 | 1 |
| OTU000114 | 3 | 0 | 0 | 0 | 0 | 0 | 3 | 1 | 2 | 119 | 1579 | 60 | 0 | 0 | 1 |
| OTU000115 | 62 | 0 | 0 | 0 | 0 | 0 | 31 | 1641 | 27 | 2 | 1 | 2 | 0 | 0 | 1 |
| OTU000116 | 40 | 0 | 0 | 0 | 0 | 1 | 29 | 1643 | 30 | 1 | 0 | 4 | 0 | 0 | 0 |
| OTU000117 | 1501 | 0 | 0 | 0 | 0 | 0 | 34 | 15 | 24 | 160 | 0 | 2 | 0 | 0 | 0 |
| OTU000118 | 1587 | 1 | 0 | 0 | 0 | 0 | 25 | 11 | 21 | 87 | 0 | 4 | 0 | 0 | 0 |
| OTU000119 | 1535 | 0 | 0 | 0 | 0 | 0 | 47 | 18 | 24 | 100 | 1 | 3 | 0 | 0 | 1 |
| OTU000120 | 46 | 0 | 0 | 0 | 0 | 0 | 26 | 18 | 1625 | 1 | 0 | 5 | 0 | 0 | 0 |
| OTU000122 | 1 | 0 | 811 | 0 | 0 | 0 | 3 | 3 | 22 | 50 | 774 | 42 | 0 | 0 | 1 |
| OTU000123 | 77 | 0 | 1 | 0 | 0 | 0 | 40 | 37 | 1545 | 0 | 0 | 2 | 0 | 0 | 1 |
| OTU000124 | 182 | 0 | 855 | 26 | 42 | 0 | 5 | 15 | 351 | 126 | 0 | 9 | 3 | 0 | 89 |
| OTU000125 | 0 | 1590 | 0 | 32 | 20 | 13 | 0 | 0 | 0 | 0 | 0 | 0 | 21 | 13 | 0 |
| OTU000126 | 39 | 0 | 0 | 0 | 0 | 0 | 32 | 22 | 1584 | 0 | 1 | 5 | 0 | 0 | 0 |
| OTU000129 | 0 | 341 | 0 | 5 | 9 | 3 | 0 | 0 | 0 | 1 | 0 | 0 | 0 | 1240 | 0 |
| OTU000130 | 1419 | 0 | 0 | 0 | 0 | 0 | 35 | 18 | 27 | 71 | 0 | 0 | 0 | 0 | 0 |
| OTU000131 | 3 | 0 | 0 | 0 | 0 | 0 | 1 | 1 | 0 | 108 | 1400 | 56 | 0 | 0 | 0 |

**Table S5** *Cont.*

| OTU ID | A1-1 | A1-2 | A1-3 | B3-1 | B3-2 | B3-3 | C2-1 | C2-2 | C2-3 | C3-1 | C3-2 | C3-3 | M0-1 | M0-2 | M0-3 |
| --- | --- | --- | --- | --- | --- | --- | --- | --- | --- | --- | --- | --- | --- | --- | --- |
| OTU000133 | 14 | 61 | 123 | 0 | 0 | 0 | 7 | 10 | 408 | 31 | 7 | 872 | 0 | 0 | 16 |
| OTU000134 | 1394 | 0 | 0 | 0 | 0 | 0 | 42 | 28 | 32 | 47 | 0 | 1 | 0 | 0 | 1 |
| OTU000136 | 1329 | 0 | 0 | 0 | 0 | 0 | 25 | 14 | 24 | 61 | 0 | 1 | 0 | 0 | 0 |
| OTU000137 | 3 | 0 | 0 | 0 | 0 | 0 | 0 | 2 | 1 | 50 | 12 | 1365 | 0 | 0 | 19 |
| OTU000138 | 5 | 0 | 0 | 0 | 0 | 0 | 2 | 1 | 0 | 58 | 1347 | 32 | 0 | 0 | 2 |
| OTU000140 | 12 | 54 | 22 | 2 | 4 | 3 | 11 | 194 | 178 | 0 | 0 | 0 | 1 | 960 | 0 |
| OTU000141 | 2 | 0 | 0 | 0 | 0 | 0 | 0 | 2 | 0 | 40 | 12 | 1343 | 0 | 0 | 7 |
| OTU000142 | 107 | 0 | 0 | 0 | 0 | 0 | 0 | 3 | 10 | 1201 | 17 | 26 | 0 | 0 | 0 |
| OTU000143 | 0 | 6 | 357 | 1 | 976 | 4 | 0 | 0 | 0 | 0 | 0 | 0 | 1 | 0 | 0 |
| OTU000144 | 2 | 2 | 1082 | 147 | 5 | 4 | 15 | 14 | 14 | 22 | 15 | 12 | 5 | 3 | 1 |
| OTU000145 | 69 | 0 | 0 | 0 | 0 | 0 | 3 | 0 | 0 | 1227 | 7 | 29 | 0 | 0 | 0 |
| OTU000146 | 39 | 0 | 69 | 0 | 0 | 0 | 346 | 352 | 526 | 0 | 1 | 1 | 0 | 0 | 0 |
| OTU000147 | 0 | 0 | 0 | 0 | 0 | 0 | 0 | 2 | 0 | 68 | 13 | 1218 | 0 | 0 | 25 |
| OTU000149 | 1149 | 0 | 0 | 0 | 0 | 0 | 20 | 26 | 22 | 62 | 1 | 2 | 0 | 0 | 0 |
| OTU000150 | 36 | 0 | 0 | 0 | 0 | 0 | 34 | 22 | 1154 | 1 | 2 | 1 | 0 | 0 | 1 |
| OTU000152 | 1180 | 1 | 0 | 0 | 0 | 0 | 10 | 4 | 6 | 39 | 0 | 0 | 0 | 0 | 0 |
| OTU000154 | 1132 | 0 | 0 | 0 | 0 | 0 | 22 | 11 | 4 | 54 | 1 | 1 | 0 | 0 | 0 |
| OTU000155 | 119 | 0 | 0 | 0 | 0 | 0 | 0 | 0 | 0 | 1039 | 20 | 45 | 0 | 0 | 0 |
| OTU000156 | 1 | 345 | 0 | 48 | 2 | 3 | 0 | 0 | 1 | 37 | 746 | 30 | 2 | 8 | 0 |
| OTU000158 | 87 | 27 | 0 | 0 | 0 | 0 | 1 | 3 | 1 | 1025 | 18 | 50 | 0 | 0 | 0 |
| OTU000159 | 94 | 0 | 0 | 0 | 0 | 0 | 0 | 0 | 0 | 1078 | 10 | 22 | 0 | 0 | 0 |
| OTU000160 | 610 | 0 | 39 | 0 | 0 | 0 | 18 | 8 | 19 | 41 | 12 | 421 | 0 | 0 | 7 |
| OTU000162 | 0 | 0 | 1162 | 0 | 0 | 0 | 0 | 0 | 0 | 0 | 0 | 0 | 0 | 0 | 0 |
| OTU000163 | 0 | 0 | 0 | 0 | 0 | 0 | 0 | 4 | 0 | 1 | 0 | 35 | 0 | 0 | 1119 |
| OTU000165 | 21 | 0 | 0 | 0 | 0 | 0 | 20 | 13 | 1079 | 2 | 0 | 0 | 0 | 0 | 0 |
| OTU000166 | 52 | 0 | 0 | 0 | 0 | 0 | 25 | 1018 | 32 | 1 | 0 | 4 | 0 | 0 | 1 |
| OTU000167 | 43 | 0 | 0 | 0 | 0 | 0 | 0 | 0 | 0 | 1066 | 3 | 9 | 0 | 0 | 0 |
| OTU000168 | 1 | 1051 | 0 | 8 | 3 | 7 | 0 | 0 | 0 | 0 | 0 | 0 | 12 | 9 | 0 |
| OTU000169 | 99 | 0 | 0 | 0 | 0 | 0 | 2 | 0 | 0 | 925 | 23 | 39 | 0 | 0 | 0 |
| OTU000170 | 32 | 0 | 0 | 0 | 0 | 0 | 28 | 1005 | 16 | 1 | 0 | 1 | 0 | 0 | 4 |
| OTU000171 | 966 | 0 | 0 | 0 | 0 | 0 | 33 | 16 | 14 | 46 | 2 | 1 | 0 | 0 | 0 |
| OTU000172 | 19 | 0 | 0 | 0 | 0 | 0 | 9 | 2 | 269 | 0 | 0 | 47 | 0 | 0 | 721 |
| OTU000173 | 60 | 0 | 0 | 0 | 0 | 0 | 0 | 0 | 0 | 970 | 9 | 25 | 0 | 0 | 0 |
| OTU000174 | 21 | 0 | 0 | 0 | 0 | 0 | 986 | 18 | 13 | 2 | 0 | 2 | 0 | 0 | 1 |
| OTU000176 | 0 | 976 | 1 | 16 | 10 | 4 | 0 | 0 | 0 | 0 | 0 | 0 | 18 | 13 | 0 |
| OTU000177 | 0 | 67 | 0 | 2 | 0 | 2 | 0 | 0 | 0 | 0 | 0 | 0 | 2 | 962 | 0 |
| OTU000178 | 0 | 53 | 0 | 1 | 0 | 3 | 0 | 0 | 0 | 0 | 0 | 0 | 0 | 962 | 2 |

**Table S5** *Cont.*

| OTU ID | A1-1 | A1-2 | A1-3 | B3-1 | B3-2 | B3-3 | C2-1 | C2-2 | C2-3 | C3-1 | C3-2 | C3-3 | M0-1 | M0-2 | M0-3 |
| --- | --- | --- | --- | --- | --- | --- | --- | --- | --- | --- | --- | --- | --- | --- | --- |
| OTU000180 | 36 | 0 | 0 | 0 | 0 | 0 | 36 | 6 | 919 | 3 | 0 | 0 | 0 | 0 | 0 |
| OTU000181 | 0 | 0 | 0 | 0 | 0 | 0 | 0 | 0 | 0 | 15 | 5 | 975 | 0 | 0 | 4 |
| OTU000182 | 856 | 0 | 0 | 0 | 0 | 0 | 30 | 14 | 17 | 51 | 0 | 3 | 3 | 0 | 0 |
| OTU000183 | 1 | 0 | 0 | 0 | 0 | 0 | 0 | 1 | 1 | 49 | 882 | 34 | 0 | 0 | 2 |
| OTU000184 | 0 | 197 | 0 | 4 | 1 | 1 | 0 | 0 | 0 | 0 | 0 | 0 | 3 | 763 | 0 |
| OTU000185 | 70 | 0 | 0 | 0 | 0 | 0 | 860 | 17 | 16 | 2 | 0 | 2 | 0 | 0 | 0 |
| OTU000186 | 826 | 0 | 0 | 0 | 0 | 0 | 21 | 10 | 18 | 83 | 0 | 5 | 0 | 0 | 0 |
| OTU000187 | 24 | 0 | 0 | 0 | 0 | 0 | 1 | 4 | 0 | 316 | 4 | 49 | 0 | 0 | 560 |
| OTU000188 | 2 | 0 | 0 | 0 | 0 | 0 | 0 | 0 | 0 | 21 | 903 | 27 | 0 | 0 | 0 |
| OTU000189 | 0 | 0 | 0 | 0 | 0 | 0 | 1 | 0 | 0 | 46 | 884 | 21 | 0 | 0 | 0 |
| OTU000190 | 0 | 0 | 945 | 0 | 0 | 0 | 0 | 0 | 0 | 0 | 0 | 0 | 0 | 0 | 0 |
| OTU000193 | 0 | 0 | 0 | 0 | 0 | 0 | 0 | 0 | 1 | 70 | 13 | 837 | 0 | 0 | 17 |
| OTU000194 | 0 | 545 | 0 | 15 | 10 | 354 | 0 | 1 | 0 | 0 | 0 | 0 | 10 | 3 | 0 |
| OTU000195 | 539 | 267 | 0 | 3 | 66 | 1 | 7 | 12 | 9 | 28 | 1 | 0 | 3 | 0 | 0 |
| OTU000196 | 38 | 0 | 0 | 0 | 0 | 0 | 1 | 1 | 0 | 871 | 8 | 17 | 0 | 0 | 0 |
| OTU000197 | 0 | 16 | 0 | 62 | 30 | 10 | 0 | 0 | 0 | 0 | 0 | 0 | 813 | 0 | 0 |
| OTU000198 | 58 | 0 | 0 | 0 | 0 | 0 | 833 | 17 | 19 | 0 | 0 | 1 | 0 | 0 | 0 |
| OTU000200 | 43 | 0 | 0 | 0 | 0 | 0 | 863 | 4 | 11 | 5 | 0 | 0 | 0 | 0 | 0 |
| OTU000201 | 0 | 25 | 898 | 1 | 0 | 0 | 0 | 0 | 0 | 0 | 0 | 0 | 0 | 0 | 0 |
| OTU000202 | 0 | 0 | 921 | 0 | 0 | 0 | 0 | 0 | 0 | 0 | 0 | 0 | 0 | 0 | 0 |
| OTU000203 | 820 | 0 | 0 | 0 | 0 | 0 | 12 | 11 | 11 | 65 | 1 | 0 | 0 | 0 | 0 |
| OTU000204 | 0 | 41 | 0 | 0 | 1 | 1 | 0 | 0 | 0 | 0 | 0 | 0 | 0 | 876 | 0 |
| OTU000205 | 1 | 22 | 0 | 4 | 1 | 0 | 0 | 0 | 0 | 0 | 0 | 10 | 0 | 323 | 552 |
| OTU000206 | 72 | 0 | 0 | 0 | 0 | 0 | 0 | 0 | 1 | 790 | 6 | 17 | 0 | 0 | 1 |
| OTU000209 | 23 | 0 | 0 | 0 | 0 | 0 | 27 | 14 | 813 | 0 | 0 | 1 | 0 | 0 | 1 |
| OTU000210 | 818 | 0 | 0 | 0 | 0 | 0 | 15 | 2 | 10 | 27 | 0 | 0 | 0 | 0 | 1 |
| OTU000211 | 3 | 0 | 0 | 0 | 0 | 0 | 1 | 6 | 0 | 30 | 8 | 810 | 0 | 0 | 8 |
| OTU000213 | 2 | 0 | 0 | 0 | 0 | 0 | 2 | 1 | 0 | 51 | 755 | 34 | 0 | 0 | 0 |
| OTU000215 | 63 | 0 | 0 | 0 | 0 | 0 | 25 | 9 | 744 | 0 | 2 | 0 | 0 | 0 | 0 |
| OTU000216 | 787 | 0 | 15 | 0 | 0 | 0 | 3 | 5 | 1 | 26 | 0 | 0 | 0 | 0 | 2 |
| OTU000217 | 0 | 121 | 704 | 2 | 0 | 2 | 4 | 0 | 0 | 0 | 0 | 0 | 1 | 5 | 0 |
| OTU000218 | 735 | 0 | 0 | 0 | 0 | 0 | 24 | 10 | 8 | 59 | 1 | 0 | 0 | 0 | 0 |
| OTU000219 | 1 | 18 | 0 | 3 | 25 | 6 | 0 | 0 | 0 | 0 | 0 | 0 | 779 | 1 | 0 |
| OTU000220 | 35 | 0 | 93 | 0 | 0 | 0 | 0 | 0 | 0 | 681 | 7 | 12 | 0 | 0 | 0 |
| OTU000222 | 3 | 527 | 0 | 69 | 23 | 28 | 0 | 0 | 3 | 0 | 0 | 0 | 143 | 15 | 0 |
| OTU000224 | 680 | 0 | 1 | 0 | 0 | 0 | 31 | 14 | 21 | 41 | 3 | 0 | 0 | 0 | 1 |
| OTU000225 | 21 | 0 | 0 | 0 | 0 | 0 | 12 | 19 | 737 | 0 | 1 | 0 | 0 | 0 | 0 |

**Table S5** *Cont.*

| OTU ID | A1-1 | A1-2 | A1-3 | B3-1 | B3-2 | B3-3 | C2-1 | C2-2 | C2-3 | C3-1 | C3-2 | C3-3 | M0-1 | M0-2 | M0-3 |
| --- | --- | --- | --- | --- | --- | --- | --- | --- | --- | --- | --- | --- | --- | --- | --- |
| OTU000226 | 0 | 0 | 0 | 0 | 0 | 0 | 0 | 2 | 0 | 31 | 733 | 22 | 0 | 0 | 0 |
| OTU000229 | 34 | 0 | 0 | 0 | 0 | 0 | 19 | 11 | 711 | 1 | 0 | 1 | 0 | 0 | 0 |
| OTU000230 | 0 | 219 | 0 | 4 | 1 | 0 | 0 | 0 | 0 | 0 | 0 | 0 | 2 | 550 | 0 |
| OTU000231 | 4 | 0 | 0 | 0 | 0 | 0 | 0 | 0 | 0 | 45 | 690 | 37 | 0 | 0 | 0 |
| OTU000232 | 21 | 0 | 0 | 0 | 0 | 0 | 6 | 14 | 733 | 0 | 1 | 0 | 0 | 0 | 0 |
| OTU000234 | 0 | 646 | 0 | 96 | 9 | 3 | 0 | 0 | 0 | 0 | 0 | 0 | 6 | 2 | 0 |
| OTU000235 | 0 | 712 | 0 | 6 | 9 | 10 | 0 | 0 | 0 | 0 | 0 | 0 | 10 | 10 | 0 |
| OTU000236 | 0 | 704 | 0 | 7 | 10 | 7 | 0 | 0 | 0 | 0 | 0 | 0 | 10 | 18 | 0 |
| OTU000237 | 0 | 0 | 0 | 0 | 0 | 0 | 0 | 0 | 0 | 37 | 688 | 27 | 0 | 0 | 1 |
| OTU000238 | 0 | 0 | 0 | 0 | 0 | 0 | 0 | 0 | 0 | 37 | 675 | 33 | 0 | 0 | 0 |
| OTU000239 | 15 | 0 | 0 | 0 | 0 | 0 | 14 | 17 | 692 | 0 | 0 | 0 | 0 | 0 | 0 |
| OTU000240 | 0 | 0 | 0 | 0 | 0 | 0 | 0 | 0 | 0 | 26 | 6 | 685 | 0 | 0 | 2 |
| OTU000241 | 629 | 0 | 0 | 0 | 0 | 0 | 14 | 12 | 11 | 45 | 1 | 0 | 0 | 0 | 0 |
| OTU000243 | 0 | 1 | 0 | 0 | 0 | 0 | 0 | 0 | 1 | 37 | 633 | 38 | 0 | 0 | 0 |
| OTU000244 | 23 | 0 | 0 | 0 | 0 | 0 | 649 | 7 | 23 | 0 | 0 | 2 | 0 | 0 | 1 |
| OTU000245 | 39 | 0 | 0 | 0 | 0 | 0 | 12 | 5 | 647 | 0 | 0 | 0 | 0 | 0 | 0 |
| OTU000246 | 23 | 0 | 83 | 0 | 0 | 0 | 317 | 2 | 275 | 0 | 0 | 0 | 0 | 0 | 0 |
| OTU000247 | 25 | 0 | 7 | 14 | 0 | 0 | 621 | 16 | 9 | 0 | 0 | 0 | 0 | 0 | 0 |
| OTU000248 | 2 | 0 | 0 | 0 | 0 | 0 | 4 | 0 | 1 | 13 | 651 | 20 | 0 | 0 | 0 |
| OTU000249 | 0 | 0 | 0 | 0 | 0 | 0 | 0 | 0 | 0 | 0 | 0 | 17 | 0 | 0 | 669 |
| OTU000250 | 0 | 7 | 0 | 5 | 15 | 4 | 0 | 0 | 0 | 0 | 1 | 0 | 648 | 0 | 0 |
| OTU000251 | 0 | 446 | 0 | 9 | 18 | 7 | 0 | 0 | 0 | 0 | 0 | 0 | 153 | 42 | 0 |
| OTU000252 | 21 | 0 | 0 | 0 | 0 | 0 | 632 | 10 | 8 | 0 | 1 | 0 | 0 | 0 | 0 |
| OTU000253 | 0 | 363 | 0 | 2 | 2 | 1 | 0 | 0 | 0 | 0 | 0 | 0 | 4 | 295 | 0 |
| OTU000255 | 578 | 0 | 0 | 0 | 0 | 0 | 12 | 9 | 14 | 42 | 0 | 0 | 0 | 0 | 0 |
| OTU000256 | 15 | 0 | 0 | 0 | 0 | 0 | 21 | 11 | 606 | 0 | 0 | 0 | 0 | 0 | 0 |
| OTU000257 | 0 | 0 | 0 | 0 | 0 | 0 | 0 | 0 | 0 | 2 | 0 | 24 | 0 | 0 | 618 |
| OTU000258 | 584 | 0 | 0 | 0 | 0 | 0 | 8 | 7 | 7 | 33 | 0 | 0 | 0 | 0 | 0 |
| OTU000259 | 0 | 32 | 0 | 0 | 0 | 0 | 0 | 0 | 0 | 0 | 0 | 0 | 0 | 595 | 0 |
| OTU000260 | 0 | 31 | 0 | 0 | 0 | 0 | 0 | 0 | 0 | 0 | 0 | 0 | 0 | 595 | 0 |
| OTU000261 | 0 | 586 | 0 | 6 | 4 | 7 | 0 | 0 | 0 | 0 | 0 | 0 | 8 | 5 | 0 |
| OTU000262 | 35 | 0 | 0 | 0 | 0 | 0 | 14 | 8 | 552 | 2 | 0 | 0 | 0 | 0 | 1 |
| OTU000264 | 551 | 0 | 0 | 0 | 0 | 0 | 6 | 6 | 7 | 21 | 0 | 1 | 0 | 0 | 0 |
| OTU000265 | 13 | 0 | 0 | 0 | 0 | 0 | 9 | 10 | 552 | 0 | 0 | 1 | 0 | 0 | 3 |
| OTU000266 | 515 | 0 | 0 | 0 | 0 | 0 | 13 | 5 | 21 | 29 | 0 | 1 | 0 | 0 | 0 |
| OTU000267 | 0 | 0 | 0 | 0 | 0 | 0 | 0 | 0 | 0 | 17 | 3 | 555 | 0 | 0 | 5 |
| OTU000268 | 532 | 0 | 0 | 0 | 0 | 0 | 15 | 7 | 7 | 17 | 0 | 0 | 0 | 0 | 0 |

**Table S5** *Cont.*

| OTU ID | A1-1 | A1-2 | A1-3 | B3-1 | B3-2 | B3-3 | C2-1 | C2-2 | C2-3 | C3-1 | C3-2 | C3-3 | M0-1 | M0-2 | M0-3 |
| --- | --- | --- | --- | --- | --- | --- | --- | --- | --- | --- | --- | --- | --- | --- | --- |
| OTU000270 | 15 | 0 | 0 | 0 | 0 | 0 | 2 | 537 | 17 | 1 | 0 | 0 | 0 | 0 | 0 |
| OTU000271 | 11 | 0 | 64 | 0 | 0 | 0 | 77 | 280 | 135 | 2 | 0 | 0 | 0 | 0 | 0 |
| OTU000272 | 4 | 0 | 0 | 0 | 0 | 0 | 0 | 0 | 0 | 11 | 8 | 541 | 0 | 0 | 4 |
| OTU000273 | 0 | 0 | 35 | 0 | 0 | 0 | 0 | 0 | 0 | 17 | 502 | 6 | 0 | 0 | 0 |
| OTU000275 | 1 | 228 | 308 | 0 | 2 | 2 | 0 | 0 | 0 | 4 | 0 | 1 | 2 | 1 | 5 |
| OTU000278 | 27 | 0 | 0 | 0 | 0 | 0 | 7 | 495 | 13 | 0 | 1 | 1 | 0 | 0 | 2 |
| OTU000279 | 0 | 0 | 0 | 0 | 0 | 0 | 0 | 0 | 0 | 21 | 497 | 13 | 0 | 0 | 2 |
| OTU000280 | 0 | 0 | 523 | 0 | 0 | 0 | 0 | 0 | 0 | 0 | 0 | 0 | 0 | 0 | 0 |
| OTU000282 | 19 | 0 | 0 | 0 | 0 | 0 | 14 | 11 | 452 | 0 | 0 | 0 | 0 | 0 | 1 |
| OTU000284 | 0 | 19 | 0 | 1 | 0 | 1 | 0 | 0 | 0 | 0 | 0 | 0 | 0 | 464 | 0 |
| OTU000285 | 1 | 0 | 0 | 0 | 0 | 0 | 1 | 0 | 0 | 8 | 460 | 13 | 0 | 0 | 0 |
| OTU000286 | 0 | 0 | 0 | 0 | 0 | 0 | 2 | 0 | 2 | 46 | 401 | 31 | 0 | 0 | 0 |
| OTU000287 | 16 | 0 | 0 | 0 | 0 | 0 | 108 | 8 | 346 | 0 | 0 | 0 | 0 | 0 | 0 |
| OTU000288 | 0 | 0 | 476 | 0 | 0 | 0 | 0 | 0 | 0 | 0 | 0 | 0 | 0 | 0 | 0 |
| OTU000290 | 0 | 2 | 0 | 4 | 1 | 4 | 0 | 0 | 0 | 0 | 0 | 0 | 454 | 0 | 0 |
| OTU000291 | 0 | 27 | 0 | 0 | 0 | 0 | 0 | 0 | 0 | 0 | 0 | 0 | 0 | 436 | 0 |
| OTU000292 | 6 | 6 | 139 | 43 | 55 | 1 | 1 | 3 | 0 | 1 | 1 | 1 | 2 | 199 | 4 |
| OTU000295 | 10 | 0 | 179 | 0 | 0 | 0 | 247 | 7 | 5 | 0 | 0 | 0 | 0 | 0 | 0 |
| OTU000296 | 20 | 0 | 0 | 0 | 0 | 0 | 0 | 0 | 0 | 404 | 8 | 11 | 0 | 0 | 0 |
| OTU000297 | 400 | 0 | 0 | 0 | 0 | 0 | 3 | 5 | 5 | 21 | 0 | 0 | 0 | 0 | 0 |
| OTU000300 | 397 | 0 | 0 | 0 | 0 | 0 | 5 | 2 | 3 | 21 | 1 | 0 | 0 | 0 | 0 |
| OTU000302 | 176 | 0 | 0 | 0 | 0 | 0 | 108 | 2 | 113 | 4 | 0 | 0 | 0 | 0 | 0 |
| OTU000306 | 0 | 0 | 393 | 0 | 0 | 0 | 0 | 0 | 0 | 0 | 0 | 0 | 0 | 0 | 0 |
| OTU000308 | 0 | 352 | 0 | 9 | 10 | 4 | 0 | 0 | 0 | 0 | 0 | 0 | 9 | 6 | 0 |
| OTU000309 | 1 | 0 | 381 | 0 | 0 | 0 | 0 | 0 | 0 | 0 | 0 | 0 | 0 | 0 | 0 |
| OTU000311 | 0 | 0 | 0 | 370 | 5 | 1 | 0 | 1 | 0 | 0 | 2 | 0 | 0 | 0 | 0 |
| OTU000312 | 3 | 0 | 331 | 25 | 5 | 4 | 0 | 2 | 3 | 2 | 0 | 1 | 0 | 0 | 1 |
| OTU000314 | 0 | 0 | 368 | 0 | 0 | 0 | 0 | 0 | 0 | 0 | 0 | 0 | 0 | 0 | 0 |
| OTU000315 | 338 | 0 | 0 | 0 | 0 | 0 | 8 | 0 | 0 | 18 | 0 | 0 | 0 | 0 | 0 |
| OTU000316 | 337 | 0 | 0 | 0 | 0 | 0 | 3 | 2 | 0 | 18 | 0 | 0 | 0 | 0 | 0 |
| OTU000318 | 3 | 0 | 295 | 45 | 1 | 2 | 1 | 3 | 1 | 3 | 2 | 1 | 0 | 0 | 0 |
| OTU000319 | 0 | 318 | 0 | 5 | 5 | 5 | 0 | 0 | 0 | 0 | 0 | 0 | 7 | 6 | 0 |
| OTU000320 | 308 | 0 | 0 | 0 | 0 | 0 | 4 | 2 | 7 | 25 | 0 | 0 | 0 | 0 | 0 |
| OTU000321 | 0 | 0 | 345 | 0 | 0 | 0 | 0 | 0 | 0 | 0 | 0 | 0 | 0 | 0 | 0 |
| OTU000322 | 316 | 0 | 1 | 0 | 0 | 0 | 3 | 3 | 4 | 15 | 0 | 0 | 0 | 0 | 2 |
| OTU000323 | 0 | 1 | 316 | 3 | 1 | 10 | 0 | 0 | 0 | 0 | 2 | 0 | 1 | 6 | 0 |
| OTU000324 | 0 | 0 | 338 | 0 | 0 | 0 | 0 | 0 | 0 | 0 | 0 | 0 | 0 | 0 | 0 |

**Table S5** *Cont.*

| OTU ID | A1-1 | A1-2 | A1-3 | B3-1 | B3-2 | B3-3 | C2-1 | C2-2 | C2-3 | C3-1 | C3-2 | C3-3 | M0-1 | M0-2 | M0-3 |
| --- | --- | --- | --- | --- | --- | --- | --- | --- | --- | --- | --- | --- | --- | --- | --- |
| OTU000325 | 0 | 8 | 0 | 1 | 147 | 175 | 0 | 0 | 0 | 0 | 0 | 0 | 4 | 0 | 0 |
| OTU000326 | 0 | 0 | 321 | 0 | 0 | 0 | 1 | 0 | 0 | 2 | 5 | 1 | 0 | 0 | 1 |
| OTU000327 | 36 | 0 | 2 | 0 | 0 | 0 | 0 | 0 | 0 | 275 | 8 | 7 | 0 | 0 | 2 |
| OTU000328 | 0 | 0 | 319 | 2 | 2 | 0 | 0 | 0 | 0 | 0 | 0 | 0 | 0 | 0 | 0 |
| OTU000330 | 0 | 0 | 321 | 0 | 0 | 0 | 0 | 0 | 0 | 0 | 0 | 0 | 0 | 0 | 0 |
| OTU000331 | 0 | 0 | 310 | 0 | 0 | 0 | 0 | 0 | 0 | 0 | 0 | 0 | 0 | 0 | 0 |
| OTU000333 | 0 | 288 | 0 | 5 | 3 | 4 | 0 | 0 | 0 | 0 | 0 | 0 | 0 | 2 | 0 |
| OTU000334 | 0 | 0 | 175 | 0 | 103 | 0 | 3 | 1 | 1 | 8 | 9 | 2 | 0 | 0 | 0 |
| OTU000335 | 1 | 2 | 231 | 16 | 29 | 1 | 0 | 0 | 2 | 0 | 0 | 0 | 0 | 16 | 1 |
| OTU000339 | 0 | 0 | 288 | 0 | 0 | 0 | 0 | 0 | 0 | 0 | 0 | 0 | 0 | 0 | 0 |
| OTU000340 | 0 | 0 | 0 | 0 | 0 | 0 | 0 | 0 | 0 | 7 | 268 | 8 | 0 | 0 | 0 |
| OTU000341 | 2 | 0 | 171 | 91 | 0 | 1 | 1 | 1 | 0 | 1 | 0 | 0 | 3 | 1 | 9 |
| OTU000342 | 0 | 0 | 277 | 0 | 0 | 0 | 0 | 0 | 0 | 0 | 0 | 0 | 1 | 0 | 0 |
| OTU000344 | 1 | 0 | 0 | 0 | 0 | 0 | 0 | 0 | 0 | 0 | 0 | 15 | 0 | 0 | 260 |
| OTU000345 | 0 | 14 | 0 | 0 | 0 | 0 | 0 | 0 | 0 | 1 | 0 | 0 | 3 | 257 | 0 |
| OTU000347 | 0 | 242 | 0 | 6 | 4 | 4 | 0 | 0 | 0 | 0 | 0 | 0 | 7 | 4 | 0 |
| OTU000348 | 13 | 0 | 0 | 0 | 0 | 0 | 3 | 2 | 248 | 0 | 0 | 0 | 0 | 0 | 0 |
| OTU000350 | 0 | 0 | 263 | 0 | 0 | 0 | 1 | 0 | 0 | 0 | 0 | 0 | 0 | 0 | 0 |
| OTU000351 | 0 | 249 | 0 | 0 | 0 | 0 | 0 | 0 | 0 | 0 | 0 | 0 | 3 | 6 | 0 |
| OTU000352 | 0 | 0 | 256 | 0 | 0 | 0 | 0 | 0 | 0 | 0 | 0 | 0 | 0 | 0 | 0 |
| OTU000353 | 0 | 226 | 0 | 8 | 5 | 1 | 0 | 0 | 0 | 0 | 0 | 0 | 4 | 5 | 0 |
| OTU000354 | 1 | 0 | 0 | 0 | 0 | 0 | 240 | 0 | 7 | 0 | 0 | 0 | 0 | 0 | 0 |
| OTU000355 | 7 | 0 | 0 | 0 | 0 | 0 | 11 | 4 | 225 | 0 | 0 | 0 | 0 | 0 | 0 |
| OTU000356 | 0 | 0 | 239 | 0 | 0 | 0 | 0 | 0 | 0 | 1 | 0 | 0 | 0 | 0 | 0 |
| OTU000358 | 0 | 141 | 0 | 46 | 5 | 1 | 0 | 0 | 0 | 0 | 0 | 0 | 4 | 39 | 0 |
| OTU000360 | 11 | 0 | 0 | 0 | 0 | 0 | 11 | 1 | 210 | 0 | 0 | 0 | 0 | 0 | 0 |
| OTU000363 | 0 | 0 | 222 | 0 | 0 | 0 | 0 | 0 | 0 | 0 | 0 | 0 | 0 | 0 | 0 |
| OTU000370 | 0 | 0 | 211 | 0 | 0 | 0 | 0 | 0 | 0 | 0 | 0 | 0 | 0 | 0 | 0 |
| OTU000373 | 0 | 0 | 203 | 0 | 0 | 0 | 0 | 0 | 0 | 0 | 0 | 0 | 0 | 0 | 0 |
| OTU000374 | 0 | 0 | 0 | 0 | 0 | 0 | 3 | 3 | 193 | 0 | 0 | 0 | 0 | 0 | 0 |
| OTU000375 | 0 | 7 | 0 | 3 | 181 | 4 | 0 | 0 | 0 | 0 | 0 | 0 | 3 | 0 | 0 |
| OTU000377 | 0 | 0 | 192 | 0 | 0 | 0 | 0 | 0 | 0 | 0 | 0 | 0 | 0 | 0 | 0 |
| OTU000378 | 0 | 173 | 0 | 0 | 0 | 8 | 0 | 0 | 0 | 0 | 0 | 0 | 4 | 5 | 0 |
| OTU000379 | 3 | 0 | 0 | 0 | 0 | 0 | 1 | 184 | 1 | 0 | 0 | 0 | 0 | 0 | 0 |
| OTU000380 | 0 | 0 | 188 | 0 | 0 | 0 | 0 | 0 | 0 | 0 | 0 | 0 | 0 | 0 | 0 |
| OTU000385 | 0 | 0 | 181 | 0 | 0 | 0 | 0 | 0 | 0 | 0 | 0 | 0 | 0 | 0 | 0 |
| OTU000386 | 0 | 0 | 181 | 0 | 0 | 0 | 0 | 0 | 0 | 0 | 0 | 0 | 0 | 0 | 0 |

**Table S5** *Cont.*

| OTU ID | A1-1 | A1-2 | A1-3 | B3-1 | B3-2 | B3-3 | C2-1 | C2-2 | C2-3 | C3-1 | C3-2 | C3-3 | M0-1 | M0-2 | M0-3 |
| --- | --- | --- | --- | --- | --- | --- | --- | --- | --- | --- | --- | --- | --- | --- | --- |
| OTU000387 | 0 | 173 | 0 | 1 | 5 | 0 | 0 | 0 | 0 | 0 | 0 | 0 | 0 | 1 | 0 |
| OTU000388 | 0 | 0 | 178 | 0 | 0 | 0 | 0 | 0 | 0 | 0 | 0 | 0 | 0 | 0 | 0 |
| OTU000389 | 6 | 0 | 0 | 0 | 0 | 0 | 5 | 3 | 164 | 0 | 0 | 0 | 0 | 0 | 0 |
| OTU000391 | 0 | 0 | 176 | 0 | 0 | 0 | 0 | 0 | 0 | 0 | 0 | 0 | 0 | 0 | 0 |
| OTU000392 | 0 | 163 | 0 | 0 | 1 | 1 | 0 | 0 | 0 | 0 | 0 | 0 | 7 | 2 | 0 |
| OTU000394 | 0 | 0 | 172 | 0 | 0 | 1 | 0 | 0 | 0 | 0 | 0 | 0 | 0 | 0 | 0 |
| OTU000395 | 142 | 0 | 0 | 0 | 0 | 0 | 8 | 3 | 6 | 11 | 0 | 0 | 0 | 0 | 0 |
| OTU000397 | 0 | 158 | 0 | 3 | 2 | 1 | 0 | 0 | 0 | 0 | 0 | 0 | 4 | 0 | 0 |
| OTU000398 | 2 | 0 | 0 | 0 | 0 | 0 | 2 | 0 | 162 | 0 | 0 | 1 | 0 | 0 | 0 |
| OTU000401 | 150 | 0 | 0 | 0 | 0 | 0 | 0 | 2 | 1 | 9 | 0 | 0 | 0 | 0 | 0 |
| OTU000404 | 0 | 152 | 0 | 2 | 1 | 1 | 0 | 0 | 0 | 0 | 0 | 0 | 0 | 5 | 0 |
| OTU000405 | 150 | 0 | 0 | 0 | 0 | 0 | 3 | 0 | 1 | 5 | 0 | 0 | 0 | 0 | 0 |
| OTU000406 | 15 | 0 | 0 | 0 | 0 | 0 | 3 | 2 | 139 | 0 | 0 | 0 | 0 | 0 | 0 |
| OTU000407 | 0 | 0 | 154 | 0 | 0 | 0 | 3 | 0 | 0 | 0 | 0 | 0 | 0 | 0 | 0 |
| OTU000409 | 0 | 0 | 152 | 0 | 0 | 0 | 0 | 0 | 0 | 0 | 0 | 0 | 0 | 0 | 0 |
| OTU000410 | 0 | 2 | 0 | 16 | 0 | 1 | 1 | 0 | 0 | 0 | 0 | 0 | 5 | 127 | 0 |
| OTU000411 | 0 | 139 | 0 | 1 | 3 | 0 | 0 | 0 | 0 | 0 | 0 | 0 | 6 | 1 | 0 |
| OTU000412 | 0 | 133 | 0 | 9 | 2 | 1 | 0 | 0 | 0 | 0 | 0 | 0 | 0 | 3 | 0 |
| OTU000413 | 0 | 0 | 144 | 0 | 0 | 0 | 0 | 0 | 0 | 0 | 0 | 0 | 0 | 0 | 0 |
| OTU000414 | 0 | 135 | 0 | 3 | 2 | 0 | 0 | 0 | 0 | 0 | 0 | 0 | 0 | 3 | 0 |
| OTU000415 | 4 | 0 | 0 | 0 | 0 | 0 | 1 | 3 | 133 | 0 | 0 | 0 | 0 | 0 | 0 |
| OTU000417 | 0 | 1 | 0 | 6 | 123 | 5 | 0 | 0 | 0 | 0 | 0 | 0 | 2 | 0 | 0 |
| OTU000418 | 0 | 0 | 134 | 0 | 0 | 0 | 0 | 0 | 0 | 0 | 0 | 0 | 0 | 0 | 0 |
| OTU000419 | 2 | 0 | 0 | 0 | 0 | 0 | 4 | 3 | 124 | 0 | 0 | 0 | 0 | 0 | 0 |
| OTU000422 | 0 | 0 | 128 | 0 | 0 | 0 | 0 | 0 | 0 | 0 | 0 | 0 | 0 | 0 | 0 |
| OTU000423 | 0 | 0 | 0 | 128 | 0 | 0 | 0 | 0 | 0 | 0 | 0 | 0 | 0 | 0 | 0 |
| OTU000424 | 0 | 0 | 0 | 0 | 0 | 0 | 0 | 0 | 1 | 11 | 107 | 4 | 0 | 0 | 0 |
| OTU000425 | 98 | 0 | 0 | 0 | 0 | 0 | 5 | 4 | 3 | 10 | 0 | 0 | 0 | 0 | 0 |
| OTU000426 | 0 | 111 | 0 | 2 | 4 | 0 | 0 | 0 | 0 | 0 | 0 | 0 | 0 | 1 | 0 |
| OTU000427 | 0 | 109 | 1 | 0 | 0 | 0 | 0 | 0 | 0 | 0 | 0 | 0 | 4 | 1 | 0 |
| OTU000428 | 1 | 0 | 72 | 0 | 0 | 0 | 0 | 0 | 42 | 0 | 0 | 0 | 0 | 0 | 0 |
| OTU000429 | 4 | 0 | 0 | 0 | 0 | 0 | 1 | 0 | 108 | 0 | 0 | 0 | 0 | 0 | 0 |
| OTU000430 | 0 | 0 | 109 | 0 | 0 | 0 | 0 | 0 | 0 | 0 | 0 | 0 | 0 | 0 | 0 |
| OTU000432 | 2 | 0 | 0 | 0 | 0 | 0 | 2 | 1 | 101 | 1 | 0 | 0 | 0 | 0 | 0 |
| OTU000434 | 99 | 0 | 0 | 0 | 0 | 0 | 0 | 1 | 0 | 6 | 0 | 0 | 0 | 0 | 0 |
| OTU000435 | 1 | 0 | 0 | 0 | 0 | 0 | 101 | 1 | 2 | 0 | 0 | 0 | 0 | 0 | 1 |
| OTU000436 | 0 | 97 | 0 | 4 | 0 | 0 | 0 | 0 | 0 | 0 | 0 | 0 | 2 | 2 | 0 |

**Table S5** *Cont.*

| OTU ID | A1-1 | A1-2 | A1-3 | B3-1 | B3-2 | B3-3 | C2-1 | C2-2 | C2-3 | C3-1 | C3-2 | C3-3 | M0-1 | M0-2 | M0-3 |
| --- | --- | --- | --- | --- | --- | --- | --- | --- | --- | --- | --- | --- | --- | --- | --- |
| OTU000437 | 0 | 0 | 105 | 0 | 0 | 0 | 0 | 0 | 0 | 0 | 0 | 0 | 0 | 0 | 0 |
| OTU000438 | 0 | 0 | 104 | 0 | 0 | 0 | 0 | 0 | 0 | 0 | 0 | 0 | 0 | 0 | 0 |
| OTU000439 | 0 | 96 | 0 | 1 | 5 | 0 | 0 | 0 | 0 | 0 | 0 | 0 | 1 | 1 | 0 |
| OTU000440 | 0 | 0 | 102 | 0 | 0 | 0 | 0 | 0 | 0 | 0 | 0 | 0 | 0 | 0 | 0 |
| OTU000441 | 0 | 91 | 0 | 2 | 0 | 0 | 0 | 0 | 0 | 0 | 0 | 0 | 4 | 2 | 0 |
| OTU000443 | 39 | 46 | 0 | 1 | 2 | 1 | 0 | 0 | 0 | 2 | 0 | 0 | 0 | 5 | 2 |
| OTU000447 | 0 | 0 | 95 | 0 | 0 | 0 | 0 | 0 | 0 | 0 | 0 | 0 | 0 | 0 | 0 |
| OTU000448 | 0 | 0 | 95 | 0 | 0 | 0 | 0 | 0 | 0 | 0 | 0 | 0 | 0 | 0 | 0 |
| OTU000450 | 91 | 0 | 0 | 0 | 0 | 0 | 0 | 0 | 0 | 2 | 0 | 0 | 0 | 0 | 0 |
| OTU000452 | 0 | 84 | 0 | 0 | 2 | 4 | 0 | 0 | 0 | 0 | 0 | 0 | 2 | 0 | 0 |
| OTU000453 | 0 | 0 | 92 | 0 | 0 | 0 | 0 | 0 | 0 | 0 | 0 | 0 | 0 | 0 | 0 |
| OTU000454 | 2 | 1 | 0 | 33 | 37 | 0 | 2 | 2 | 3 | 0 | 3 | 0 | 0 | 0 | 9 |
| OTU000455 | 2 | 0 | 0 | 0 | 0 | 0 | 0 | 0 | 89 | 0 | 0 | 0 | 0 | 0 | 0 |
| OTU000458 | 0 | 87 | 0 | 0 | 0 | 0 | 0 | 0 | 0 | 0 | 0 | 0 | 0 | 0 | 0 |
| OTU000460 | 0 | 1 | 0 | 82 | 1 | 1 | 0 | 0 | 0 | 0 | 0 | 0 | 0 | 0 | 0 |
| OTU000461 | 2 | 0 | 79 | 0 | 0 | 0 | 0 | 2 | 0 | 0 | 0 | 0 | 0 | 0 | 0 |
| OTU000462 | 0 | 3 | 0 | 0 | 80 | 0 | 0 | 0 | 0 | 0 | 0 | 0 | 0 | 0 | 0 |
| OTU000464 | 0 | 3 | 0 | 0 | 79 | 0 | 0 | 0 | 0 | 0 | 0 | 0 | 0 | 0 | 0 |
| OTU000467 | 0 | 1 | 2 | 51 | 0 | 0 | 1 | 1 | 1 | 3 | 4 | 6 | 7 | 0 | 2 |
| OTU000468 | 4 | 0 | 0 | 0 | 0 | 0 | 3 | 0 | 72 | 0 | 0 | 0 | 0 | 0 | 0 |
| OTU000469 | 0 | 0 | 77 | 0 | 0 | 0 | 0 | 0 | 0 | 0 | 0 | 0 | 0 | 0 | 0 |
| OTU000470 | 0 | 5 | 0 | 0 | 64 | 0 | 0 | 0 | 0 | 0 | 0 | 0 | 4 | 0 | 1 |
| OTU000473 | 0 | 0 | 72 | 0 | 0 | 0 | 0 | 0 | 0 | 0 | 0 | 0 | 0 | 0 | 0 |
| OTU000476 | 0 | 2 | 0 | 3 | 59 | 1 | 0 | 0 | 0 | 0 | 0 | 0 | 0 | 0 | 0 |
| OTU000478 | 0 | 0 | 0 | 0 | 64 | 0 | 0 | 0 | 0 | 0 | 0 | 0 | 0 | 0 | 0 |
| OTU000479 | 0 | 59 | 0 | 0 | 0 | 1 | 0 | 0 | 0 | 0 | 0 | 0 | 1 | 2 | 0 |
| OTU000481 | 0 | 0 | 62 | 0 | 0 | 0 | 0 | 0 | 0 | 0 | 0 | 0 | 0 | 0 | 0 |
| OTU000483 | 0 | 0 | 52 | 0 | 0 | 0 | 0 | 0 | 0 | 0 | 0 | 0 | 0 | 0 | 0 |
| OTU000485 | 0 | 50 | 0 | 0 | 0 | 0 | 0 | 0 | 0 | 0 | 0 | 0 | 1 | 1 | 0 |
| OTU000489 | 0 | 0 | 30 | 0 | 0 | 0 | 0 | 0 | 0 | 1 | 0 | 17 | 0 | 0 | 0 |
| OTU000490 | 0 | 0 | 0 | 45 | 0 | 3 | 0 | 0 | 0 | 0 | 0 | 0 | 0 | 0 | 0 |
| OTU000491 | 0 | 0 | 47 | 0 | 0 | 0 | 0 | 0 | 0 | 0 | 0 | 0 | 0 | 0 | 0 |
| OTU000493 | 0 | 0 | 47 | 0 | 0 | 0 | 0 | 0 | 0 | 0 | 0 | 0 | 0 | 0 | 0 |
| OTU000494 | 0 | 1 | 0 | 1 | 44 | 0 | 0 | 0 | 0 | 0 | 0 | 0 | 0 | 0 | 0 |
| OTU000499 | 0 | 29 | 0 | 5 | 4 | 1 | 0 | 0 | 0 | 0 | 0 | 0 | 4 | 0 | 0 |
| OTU000501 | 0 | 0 | 42 | 0 | 0 | 0 | 0 | 0 | 0 | 0 | 0 | 0 | 0 | 0 | 0 |
| OTU000502 | 0 | 0 | 0 | 38 | 0 | 0 | 0 | 0 | 0 | 0 | 0 | 0 | 1 | 0 | 0 |

**Table S5** *Cont.*

| OTU ID | A1-1 | A1-2 | A1-3 | B3-1 | B3-2 | B3-3 | C2-1 | C2-2 | C2-3 | C3-1 | C3-2 | C3-3 | M0-1 | M0-2 | M0-3 |
| --- | --- | --- | --- | --- | --- | --- | --- | --- | --- | --- | --- | --- | --- | --- | --- |
| OTU000506 | 0 | 0 | 38 | 0 | 0 | 0 | 0 | 0 | 0 | 0 | 0 | 0 | 0 | 0 | 0 |
| OTU000508 | 0 | 0 | 0 | 35 | 1 | 0 | 0 | 0 | 0 | 0 | 0 | 0 | 0 | 0 | 0 |
| OTU000509 | 0 | 33 | 0 | 0 | 0 | 2 | 0 | 0 | 0 | 0 | 0 | 0 | 0 | 0 | 0 |
| OTU000510 | 0 | 0 | 0 | 34 | 0 | 0 | 0 | 1 | 0 | 0 | 0 | 0 | 0 | 0 | 0 |
| OTU000511 | 0 | 0 | 32 | 0 | 0 | 0 | 0 | 0 | 0 | 0 | 0 | 0 | 0 | 0 | 0 |
| OTU000512 | 0 | 0 | 0 | 1 | 0 | 0 | 0 | 0 | 31 | 0 | 0 | 0 | 0 | 0 | 0 |
| OTU000513 | 0 | 0 | 0 | 0 | 32 | 0 | 0 | 0 | 0 | 0 | 0 | 0 | 0 | 0 | 0 |
| OTU000514 | 0 | 0 | 0 | 0 | 0 | 0 | 0 | 0 | 0 | 0 | 0 | 0 | 0 | 0 | 31 |
| OTU000519 | 0 | 0 | 0 | 26 | 0 | 0 | 0 | 0 | 0 | 0 | 0 | 0 | 0 | 0 | 0 |
| OTU000520 | 0 | 0 | 25 | 0 | 0 | 0 | 0 | 0 | 0 | 0 | 0 | 0 | 0 | 0 | 0 |
| OTU000524 | 11 | 0 | 0 | 0 | 0 | 0 | 0 | 4 | 3 | 2 | 0 | 1 | 0 | 0 | 0 |
| OTU000525 | 0 | 0 | 0 | 20 | 0 | 0 | 0 | 0 | 0 | 0 | 0 | 0 | 0 | 0 | 0 |
| OTU000527 | 0 | 2 | 0 | 16 | 1 | 1 | 0 | 0 | 0 | 0 | 0 | 0 | 0 | 0 | 0 |
| OTU000528 | 0 | 0 | 19 | 0 | 0 | 0 | 0 | 0 | 0 | 0 | 0 | 0 | 0 | 0 | 0 |
| OTU000530 | 0 | 0 | 0 | 0 | 0 | 0 | 0 | 0 | 16 | 0 | 0 | 0 | 0 | 0 | 0 |
| OTU000531 | 0 | 0 | 0 | 16 | 0 | 0 | 0 | 0 | 0 | 0 | 0 | 0 | 0 | 0 | 0 |
| OTU000535 | 0 | 0 | 0 | 0 | 14 | 0 | 0 | 0 | 1 | 0 | 0 | 0 | 0 | 0 | 0 |
| OTU000539 | 0 | 2 | 0 | 0 | 0 | 1 | 3 | 3 | 1 | 0 | 0 | 0 | 2 | 0 | 1 |
| OTU000543 | 0 | 0 | 11 | 0 | 0 | 0 | 0 | 1 | 0 | 0 | 0 | 0 | 0 | 0 | 0 |
| OTU000548 | 0 | 8 | 0 | 0 | 0 | 1 | 0 | 0 | 0 | 0 | 0 | 0 | 0 | 0 | 0 |
| OTU000549 | 0 | 0 | 0 | 0 | 1 | 0 | 3 | 1 | 2 | 0 | 0 | 1 | 0 | 0 | 0 |
| OTU000550 | 0 | 0 | 0 | 5 | 0 | 0 | 0 | 1 | 0 | 0 | 1 | 1 | 0 | 0 | 0 |
| OTU000551 | 8 | 0 | 0 | 0 | 0 | 0 | 0 | 0 | 0 | 0 | 0 | 0 | 0 | 0 | 0 |
| OTU000554 | 0 | 0 | 8 | 0 | 0 | 0 | 0 | 0 | 0 | 0 | 0 | 0 | 0 | 0 | 0 |
| OTU000555 | 0 | 0 | 0 | 0 | 0 | 0 | 6 | 0 | 1 | 0 | 0 | 1 | 0 | 0 | 0 |
| OTU000556 | 0 | 0 | 0 | 0 | 0 | 0 | 0 | 0 | 0 | 0 | 0 | 8 | 0 | 0 | 0 |
| OTU000557 | 0 | 0 | 7 | 0 | 0 | 0 | 0 | 0 | 0 | 0 | 0 | 0 | 0 | 0 | 0 |
| OTU000558 | 0 | 0 | 0 | 0 | 0 | 0 | 1 | 0 | 0 | 5 | 0 | 1 | 0 | 0 | 0 |
| OTU000559 | 0 | 0 | 7 | 0 | 0 | 0 | 0 | 0 | 0 | 0 | 0 | 0 | 0 | 0 | 0 |
| OTU000561 | 2 | 0 | 0 | 0 | 0 | 0 | 0 | 1 | 0 | 0 | 1 | 3 | 0 | 0 | 0 |
| OTU000562 | 0 | 5 | 0 | 0 | 0 | 0 | 0 | 0 | 1 | 0 | 0 | 0 | 0 | 0 | 0 |
| OTU000564 | 0 | 0 | 2 | 0 | 0 | 0 | 0 | 0 | 1 | 0 | 0 | 0 | 0 | 0 | 3 |
| OTU000565 | 0 | 3 | 0 | 3 | 0 | 0 | 0 | 0 | 0 | 0 | 0 | 0 | 0 | 0 | 0 |
| OTU000566 | 0 | 0 | 0 | 0 | 0 | 0 | 0 | 2 | 4 | 0 | 0 | 0 | 0 | 0 | 0 |
| OTU000569 | 0 | 0 | 0 | 0 | 0 | 0 | 0 | 1 | 0 | 5 | 0 | 0 | 0 | 0 | 0 |
| OTU000571 | 2 | 0 | 4 | 0 | 0 | 0 | 0 | 0 | 0 | 0 | 0 | 0 | 0 | 0 | 0 |
| OTU000573 | 0 | 0 | 1 | 0 | 0 | 0 | 0 | 0 | 0 | 2 | 0 | 1 | 0 | 0 | 1 |

**Table S5** *Cont.*

| OTU ID | A1-1 | A1-2 | A1-3 | B3-1 | B3-2 | B3-3 | C2-1 | C2-2 | C2-3 | C3-1 | C3-2 | C3-3 | M0-1 | M0-2 | M0-3 |
| --- | --- | --- | --- | --- | --- | --- | --- | --- | --- | --- | --- | --- | --- | --- | --- |
| OTU000574 | 0 | 0 | 0 | 0 | 0 | 0 | 0 | 3 | 0 | 1 | 1 | 0 | 0 | 0 | 0 |
| OTU000576 | 0 | 0 | 0 | 0 | 0 | 0 | 0 | 0 | 0 | 3 | 0 | 0 | 0 | 0 | 2 |
| OTU000577 | 0 | 0 | 5 | 0 | 0 | 0 | 0 | 0 | 0 | 0 | 0 | 0 | 0 | 0 | 0 |
| OTU000579 | 0 | 0 | 0 | 0 | 2 | 2 | 0 | 0 | 0 | 0 | 0 | 0 | 1 | 0 | 0 |
| OTU000580 | 0 | 0 | 0 | 0 | 0 | 0 | 0 | 0 | 4 | 0 | 0 | 0 | 0 | 0 | 0 |
| OTU000581 | 1 | 0 | 0 | 0 | 0 | 0 | 1 | 0 | 1 | 0 | 1 | 0 | 0 | 0 | 0 |
| OTU000582 | 0 | 0 | 0 | 0 | 0 | 0 | 0 | 0 | 0 | 0 | 0 | 0 | 4 | 0 | 0 |
| OTU000583 | 2 | 0 | 0 | 0 | 0 | 0 | 1 | 0 | 0 | 0 | 0 | 1 | 0 | 0 | 0 |
| OTU000584 | 0 | 0 | 0 | 0 | 0 | 0 | 0 | 0 | 4 | 0 | 0 | 0 | 0 | 0 | 0 |
| OTU000585 | 0 | 0 | 0 | 0 | 0 | 0 | 0 | 4 | 0 | 0 | 0 | 0 | 0 | 0 | 0 |
| OTU000589 | 0 | 0 | 0 | 0 | 0 | 0 | 0 | 0 | 0 | 0 | 0 | 0 | 0 | 0 | 3 |
| OTU000590 | 0 | 0 | 0 | 0 | 3 | 0 | 0 | 0 | 0 | 0 | 0 | 0 | 0 | 0 | 0 |
| OTU000591 | 0 | 0 | 0 | 0 | 0 | 0 | 0 | 2 | 0 | 0 | 1 | 0 | 0 | 0 | 0 |
| OTU000592 | 0 | 0 | 0 | 0 | 0 | 0 | 3 | 0 | 0 | 0 | 0 | 0 | 0 | 0 | 0 |
| OTU000593 | 0 | 0 | 0 | 0 | 0 | 0 | 0 | 3 | 0 | 0 | 0 | 0 | 0 | 0 | 0 |
| OTU000595 | 0 | 0 | 0 | 0 | 0 | 0 | 0 | 3 | 0 | 0 | 0 | 0 | 0 | 0 | 0 |
| OTU000597 | 0 | 0 | 0 | 0 | 0 | 0 | 0 | 0 | 0 | 0 | 2 | 0 | 0 | 0 | 0 |
| OTU000598 | 0 | 0 | 0 | 0 | 0 | 2 | 0 | 0 | 0 | 0 | 0 | 0 | 0 | 0 | 0 |
| OTU000600 | 0 | 0 | 0 | 0 | 0 | 0 | 0 | 2 | 0 | 0 | 0 | 0 | 0 | 0 | 0 |
| OTU000601 | 2 | 0 | 0 | 0 | 0 | 0 | 0 | 0 | 0 | 0 | 0 | 0 | 0 | 0 | 0 |
| OTU000602 | 0 | 0 | 0 | 0 | 0 | 0 | 0 | 0 | 0 | 2 | 0 | 0 | 0 | 0 | 0 |
| OTU000603 | 0 | 0 | 0 | 0 | 0 | 0 | 0 | 0 | 0 | 0 | 0 | 0 | 0 | 0 | 2 |

Table S6 OTU identification and classification information

| OTU ID | Phylum | Class | Order | Family | Genus | Species |
| --- | --- | --- | --- | --- | --- | --- |
| OTU000001 | Ascomycota | Saccharomycetes | Saccharomycetales | Saccharomycetaceae | Saccharomyces |  |
| OTU000003 | Ascomycota |  |  |  |  |  |
| OTU000004 | Ascomycota | Dothideomycetes | Capnodiales | Cladosporiaceae | Cladosporium | Cladosporium_halotolerans |
| OTU000005 | Ascomycota | Saccharomycetes | Saccharomycetales | Metschnikowiaceae | Metschnikowia | Metschnikowia_bicuspidata |
| OTU000007 | Basidiomycota | Agaricomycetes | Agaricales | Lyophyllaceae | Fibulochlamys | Fibulochlamys_chilensis |
| OTU000010 | Ascomycota | Dothideomycetes | Capnodiales | Cladosporiaceae | Cladosporium | Cladosporium_cladosporioides |
| OTU000011 | Ascomycota | Sordariomycetes | Hypocreales | Cordycipitaceae | Simplicillium | Simplicillium_aogashimaense |
| OTU000012 | Ascomycota | Sordariomycetes | Coniochaetales | Coniochaetaceae | Coniochaeta |  |
| OTU000013 | Ascomycota | Saccharomycetes | Saccharomycetales | Saccharomycodaceae | Hanseniaspora | Hanseniaspora_uvarum |
| OTU000014 | Ascomycota | Saccharomycetes | Saccharomycetales | Saccharomycetales_fam_Incertae_sedis |  |  |
| OTU000015 | Ascomycota | Sordariomycetes | Sordariales | Chaetomiaceae |  |  |
| OTU000016 | Ascomycota | Sordariomycetes | Hypocreales | Hypocreaceae | Trichoderma |  |
| OTU000017 | Ascomycota | Eurotiomycetes | Eurotiales | Aspergillaceae | Aspergillus | Aspergillus_penicillioides |
| OTU000018 | Ascomycota | Sordariomycetes | Sordariales | Cephalothecaceae | Phialemonium | Phialemonium_inflatum |
| OTU000019 | Ascomycota | Sordariomycetes | Hypocreales | Hypocreaceae | Trichoderma |  |
| OTU000020 | Basidiomycota | Tremellomycetes | Tremellales | Trimorphomycetaceae | Saitozyma | Saitozyma_podzolica |
| OTU000021 | Mortierellomycota | Mortierellomycetes | Mortierellales | Mortierellaceae | Mortierella | Mortierella_elongata |
| OTU000022 | Ascomycota | Saccharomycetes | Saccharomycetales | Saccharomycetaceae | Kazachstania |  |
| OTU000023 | Ascomycota | Sordariomycetes | Hypocreales | Nectriaceae | Fusarium |  |
| OTU000024 | Ascomycota | Eurotiomycetes | Eurotiales | Aspergillaceae | Aspergillus | Aspergillus_penicillioides |
| OTU000025 | Ascomycota | Dothideomycetes | Pleosporales | Didymellaceae |  |  |
| OTU000027 | Ascomycota | Leotiomycetes | Thelebolales | Pseudeurotiaceae | Pseudogymnoascus |  |
| OTU000028 | Ascomycota | Eurotiomycetes | Eurotiales | Aspergillaceae | Aspergillus | Aspergillus_conicus |
| OTU000029 | Basidiomycota | Agaricomycetes | Agaricales | Lyophyllaceae | Tephrocybe | Tephrocybe_anthracophila |
| OTU000030 | Ascomycota | Saccharomycetes | Saccharomycetales | Dipodascaceae |  |  |
| OTU000031 | Ascomycota | Saccharomycetes | Saccharomycetales | Saccharomycetaceae | Nakaseomyces |  |
| OTU000032 | Ascomycota | Eurotiomycetes | Eurotiales | Trichocomaceae | Talaromyces |  |
| OTU000033 | Ascomycota | Eurotiomycetes | Eurotiales | Aspergillaceae | Penicillium |  |
| OTU000034 | Ascomycota | Dothideomycetes | Pleosporales | Pleosporaceae | Alternaria |  |
| OTU000035 | Ascomycota | Sordariomycetes | Microascales | Microascaceae | Cephalotrichum |  |
| OTU000036 | Basidiomycota | Agaricomycetes | Agaricales | Lyophyllaceae | Fibulochlamys | Fibulochlamys_chilensis |
| OTU000037 | Ascomycota | Sordariomycetes | Hypocreales | Nectriaceae | Fusicolla | Fusicolla_acetilerea |
| OTU000038 | Ascomycota | Eurotiomycetes | Eurotiales | Aspergillaceae | Aspergillus |  |
| OTU000039 | Ascomycota | Sordariomycetes | Hypocreales | Stachybotryaceae | Stachybotrys | Stachybotrys_chartarum |
| OTU000040 | Basidiomycota | Agaricomycetes | Agaricales | Entolomataceae | Clitopilus |  |
| OTU000041 | Ascomycota | Eurotiomycetes | Eurotiales | Aspergillaceae | Aspergillus |  |

**Table S6** *Cont.*

| OTU ID | Phylum | Class | Order | Family | Genus | Species |
| --- | --- | --- | --- | --- | --- | --- |
| OTU000042 | Basidiomycota | Tremellomycetes | Trichosporonales | Trichosporonaceae | Cutaneotrichosporon | Cutaneotrichosporon_debeurmannianum |
| OTU000044 | Ascomycota | Eurotiomycetes | Eurotiales | Aspergillaceae | Aspergillus |  |
| OTU000045 | Ascomycota | Dothideomycetes | Capnodiales | Cladosporiaceae | Toxicocladosporium |  |
| OTU000046 | Ascomycota | Sordariomycetes | Hypocreales | Nectriaceae | Fusarium | Fusarium_solani |
| OTU000047 | Ascomycota | Dothideomycetes | Pleosporales | Morosphaeriaceae |  |  |
| OTU000049 | Basidiomycota | Agaricomycetes | Agaricales | Pluteaceae | Pluteus | Pluteus_umbrosoides |
| OTU000051 | Ascomycota | Saccharomycetes | Saccharomycetales | Metschnikowiaceae | Kodamaea | Kodamaea_ohmeri |
| OTU000054 | Mortierellomycota | Mortierellomycetes | Mortierellales | Mortierellaceae | Mortierella | Mortierella_alpina |
| OTU000055 | Basidiomycota |  |  |  |  |  |
| OTU000056 | Ascomycota | Saccharomycetes | Saccharomycetales | Saccharomycetales_fam_Incertae_sedis | Candida |  |
| OTU000057 | Ascomycota | Eurotiomycetes | Eurotiales | Aspergillaceae | Aspergillus |  |
| OTU000058 | Ascomycota | Dothideomycetes | Dothideales | Dothideales_fam_Incertae_sedis | Hortaea | Hortaea_werneckii |
| OTU000059 | Ascomycota | Eurotiomycetes | Eurotiales | Aspergillaceae | Aspergillus | Aspergillus_chlamydosporus |
| OTU000060 | Basidiomycota | Tremellomycetes | Filobasidiales | Piskurozymaceae | Solicoccozyma | Solicoccozyma_aeria |
| OTU000061 | Ascomycota | Sordariomycetes | Sordariales |  |  |  |
| OTU000062 | Ascomycota | Sordariomycetes | Hypocreales | Nectriaceae | Nectria | Nectria_ramulariae |
| OTU000063 | Ascomycota | Saccharomycetes | Saccharomycetales | Saccharomycetales_fam_Incertae_sedis | Candida | Candida_nivariensis |
| OTU000064 |  |  |  |  |  |  |
| OTU000065 | Mucoromycota | Mucoromycetes | Mucorales | Cunninghamellaceae | Gongronella | Gongronella_butleri |
| OTU000066 | Ascomycota | Saccharomycetes | Saccharomycetales | Saccharomycetaceae | Issatchenkia | Issatchenkia_orientalis |
| OTU000067 | Ascomycota | Sordariomycetes | Microascales | Microascaceae |  |  |
| OTU000068 | Basidiomycota | Agaricomycetes | Tremellodendropsidales |  |  |  |
| OTU000069 | Ascomycota | Sordariomycetes | Hypocreales | Nectriaceae | Gibberella |  |
| OTU000070 | Ascomycota | Sordariomycetes | Hypocreales | Nectriaceae | Fusarium |  |
| OTU000071 | Ascomycota | Sordariomycetes | Trichosphaeriales | Trichosphaeriaceae | Nigrospora | Nigrospora_oryzae |
| OTU000072 | Ascomycota | Sordariomycetes | Hypocreales | Bionectriaceae | Clonostachys |  |
| OTU000073 | Basidiomycota | Tremellomycetes | Trichosporonales | Trichosporonaceae |  |  |
| OTU000074 | Basidiomycota | Agaricomycetes | Trechisporales | Hydnodontaceae | Subulicystidium |  |
| OTU000075 | Basidiomycota | Agaricostilbomycetes | Agaricostilbales | Agaricostilbaceae | Sterigmatomyces | Sterigmatomyces_halophilus |
| OTU000076 | Ascomycota | Dothideomycetes | Dothideales | Aureobasidiaceae | Aureobasidium |  |
| OTU000077 | Ascomycota | Eurotiomycetes | Chaetothyriales | Herpotrichiellaceae |  |  |
| OTU000078 | Ascomycota |  |  |  |  |  |
| OTU000079 | Ascomycota | Sordariomycetes | Sordariales | Lasiosphaeriaceae | Immersiella | Immersiella_caudata |
| OTU000080 | Ascomycota | Eurotiomycetes | Eurotiales | Aspergillaceae | Aspergillus | Aspergillus_hiratsukae |
| OTU000081 | Ascomycota | Dothideomycetes | Pleosporales | Phaeosphaeriaceae | Paraphoma | Paraphoma_radicina |
| OTU000082 |  |  |  |  |  |  |
| OTU000083 | Ascomycota | Eurotiomycetes | Eurotiales | Aspergillaceae | Aspergillus |  |

**Table S6** *Cont.*

| OTU ID | Phylum | Class | Order | Family | Genus | Species |
| --- | --- | --- | --- | --- | --- | --- |
| OTU000085 | Ascomycota | Eurotiomycetes | Chaetothyriales |  |  |  |
| OTU000086 |  |  |  |  |  |  |
| OTU000087 | Ascomycota | Dothideomycetes | Pleosporales | Periconiaceae | Periconia |  |
| OTU000088 | Ascomycota | Sordariomycetes | Hypocreales | Nectriaceae | Fusarium | Fusarium_solani |
| OTU000089 |  |  |  |  |  |  |
| OTU000090 | Ascomycota | Eurotiomycetes | Chaetothyriales | Herpotrichiellaceae | Cladophialophora |  |
| OTU000091 | Basidiomycota | Agaricomycetes | Agaricales | Clavariaceae | Hodophilus |  |
| OTU000092 | Ascomycota | Saccharomycetes | Saccharomycetales | Metschnikowiaceae | Metschnikowia | Metschnikowia_bicuspidata |
| OTU000093 | Ascomycota | Leotiomycetes | Thelebolales | Pseudeurotiaceae | Pseudeurotium |  |
| OTU000094 | Basidiomycota | Tremellomycetes | Trichosporonales | Trichosporonaceae | Cutaneotrichosporon | Cutaneotrichosporon_moniliiforme |
| OTU000095 | Ascomycota | Eurotiomycetes | Eurotiales | Thermoascaceae | Byssochlamys | Byssochlamys_zollerniae |
| OTU000097 | Ascomycota |  |  |  |  |  |
| OTU000100 | Ascomycota | Saccharomycetes | Saccharomycetales |  |  |  |
| OTU000101 | Ascomycota | Eurotiomycetes | Chaetothyriales |  |  |  |
| OTU000103 | Ascomycota | Leotiomycetes | Helotiales | Helotiaceae | Tetracladium | Tetracladium_apiense |
| OTU000104 | Ascomycota | Sordariomycetes | Sordariales | Chaetomiaceae | Corynascella | Corynascella_humicola |
| OTU000105 | Ascomycota |  |  |  |  |  |
| OTU000106 | Ascomycota | Sordariomycetes | Sordariales | Lasiosphaeriaceae |  |  |
| OTU000107 | Ascomycota | Saccharomycetes | Saccharomycetales | Saccharomycetales_fam_Incertae_sedis | Candida | Candida_quercitrusa |
| OTU000108 | Ascomycota | Eurotiomycetes | Eurotiales | Aspergillaceae | Penicillium |  |
| OTU000109 | Ascomycota | Dothideomycetes | Pleosporales |  |  |  |
| OTU000110 | Basidiomycota | Agaricomycetes | Russulales | Peniophoraceae | Peniophora | Peniophora_lycii |
| OTU000114 | Ascomycota | Sordariomycetes | Sordariales | Chaetomiaceae |  |  |
| OTU000115 | Ascomycota | Dothideomycetes | Pleosporales | Pleosporaceae | Curvularia | Curvularia_lunata |
| OTU000116 | Basidiomycota | Agaricomycetes | Agaricales | Lycoperdaceae | Calvatia | Calvatia_fragilis |
| OTU000117 | Ascomycota | Sordariomycetes | Glomerellales | Plectosphaerellaceae | Gibellulopsis | Gibellulopsis_piscis |
| OTU000118 | Ascomycota | Pezizomycetes | Pezizales | Ascodesmidaceae | Cephaliophora | Cephaliophora_tropica |
| OTU000119 | Ascomycota | Sordariomycetes | Hypocreales | Nectriaceae | Dactylonectria | Dactylonectria_macrodidyma |
| OTU000120 | Ascomycota | Sordariomycetes | Sordariales | Sordariales_fam_Incertae_sedis | Conlarium |  |
| OTU000122 | Ascomycota | Sordariomycetes | Hypocreales | Nectriaceae | Fusarium | Fusarium_concentricum |
| OTU000123 | Ascomycota | Leotiomycetes | Helotiales | Helotiales_fam_Incertae_sedis | Chalara |  |
| OTU000124 | Basidiomycota | Malasseziomycetes | Malasseziales | Malasseziaceae | Malassezia | Malassezia_restricta |
| OTU000125 | Ascomycota | Sordariomycetes | Sordariales | Chaetomiaceae | Zopfiella | Zopfiella_tardifaciens |
| OTU000126 | Basidiomycota | Agaricomycetes | Agaricales | Hymenogastraceae | Hebeloma |  |
| OTU000129 | Ascomycota | Pezizomycetes | Pezizales | Pezizaceae | Peziza | Peziza_ostracoderma |
| OTU000130 | Ascomycota | Eurotiomycetes | Chaetothyriales | Herpotrichiellaceae | Exophiala | Exophiala_pisciphila |
| OTU000131 | Ascomycota | Sordariomycetes | Hypocreales | Bionectriaceae | Clonostachys |  |

**Table S6** *Cont.*

| OTU ID | Phylum | Class | Order | Family | Genus | Species |
| --- | --- | --- | --- | --- | --- | --- |
| OTU000133 | Ascomycota | Dothideomycetes | Pleosporales | Didymosphaeriaceae | Paraphaeosphaeria | Paraphaeosphaeria_angularis |
| OTU000134 | Ascomycota | Dothideomycetes | Capnodiales | Mycosphaerellaceae | Cercospora |  |
| OTU000136 | Ascomycota | Eurotiomycetes | Chaetothyriales |  |  |  |
| OTU000137 | Ascomycota | Leotiomycetes | Helotiales |  |  |  |
| OTU000138 | Basidiomycota | Agaricomycetes | Agaricales | Tricholomataceae | Mycena |  |
| OTU000140 | Ascomycota | Dothideomycetes | Capnodiales | Teratosphaeriaceae | Eupenidiella | Eupenidiella_venezuelensis |
| OTU000141 | Basidiomycota | Tremellomycetes | Holtermanniales | Holtermanniales_fam_Incertae_sedis | Holtermanniella |  |
| OTU000142 | Ascomycota |  |  |  |  |  |
| OTU000143 | Ascomycota | Saccharomycetes | Saccharomycetales | Saccharomycetales_fam_Incertae_sedis | Candida | Candida_apicola |
| OTU000144 | Ascomycota | Leotiomycetes | Helotiales | Sclerotiniaceae |  |  |
| OTU000145 | Ascomycota | Leotiomycetes | Helotiales | Helotiaceae | Tetracladium |  |
| OTU000146 | Ascomycota | Eurotiomycetes | Eurotiales | Aspergillaceae | Penicillium |  |
| OTU000147 | Ascomycota |  |  |  |  |  |
| OTU000149 | Ascomycota | Sordariomycetes | Hypocreales | Hypocreales_fam_Incertae_sedis |  |  |
| OTU000150 | Ascomycota |  |  |  |  |  |
| OTU000152 | Mucoromycota | Mucoromycetes | Mucorales | Mucoraceae | Mucor | Mucor_racemosus |
| OTU000154 | Ascomycota | Dothideomycetes | Pleosporales |  |  |  |
| OTU000155 | Ascomycota | Sordariomycetes |  |  |  |  |
| OTU000156 | Ascomycota | Sordariomycetes | Sordariales | Chaetomiaceae | Chaetomium |  |
| OTU000158 | Ascomycota | Sordariomycetes | Sordariales | Sordariales_fam_Incertae_sedis | Staphylotrichum |  |
| OTU000159 |  |  |  |  |  |  |
| OTU000160 | Ascomycota | Sordariomycetes | Hypocreales | Nectriaceae | Fusarium |  |
| OTU000162 | Basidiomycota | Microbotryomycetes | Sporidiobolales | Sporidiobolaceae | Rhodotorula | Rhodotorula_toruloides |
| OTU000163 |  |  |  |  |  |  |
| OTU000165 |  |  |  |  |  |  |
| OTU000166 | Ascomycota | Eurotiomycetes | Eurotiales | Aspergillaceae | Aspergillus |  |
| OTU000167 | Chytridiomycota | Rhizophydiomycetes | Rhizophydiales | Terramycetaceae | Boothiomyces | Boothiomyces_macroporosum |
| OTU000168 | Ascomycota | Sordariomycetes | Pleurotheciales | Pleurotheciaceae |  |  |
| OTU000169 | Ascomycota | Sordariomycetes |  |  |  |  |
| OTU000170 | Basidiomycota | Agaricomycetes | Trechisporales |  |  |  |
| OTU000171 | Ascomycota | Sordariomycetes | Sordariales |  |  |  |
| OTU000172 | Ascomycota | Eurotiomycetes | Eurotiales | Aspergillaceae | Aspergillus | Aspergillus_penicillioides |
| OTU000173 | Ascomycota |  |  |  |  |  |
| OTU000174 | Ascomycota | Dothideomycetes | Pleosporales | Pyrenochaetopsidaceae | Neopyrenochaeta |  |
| OTU000176 | Ascomycota | Leotiomycetes | Thelebolales | Pseudeurotiaceae | Pseudogymnoascus |  |
| OTU000177 | Ascomycota | Sordariomycetes | Hypocreales | Clavicipitaceae | Metarhizium | Metarhizium_marquandii |
| OTU000178 | Ascomycota | Sordariomycetes | Sordariales | Lasiosphaeriaceae | Podospora | Podospora_communis |

**Table S6** *Cont.*

| OTU ID | Phylum | Class | Order | Family | Genus | Species |
| --- | --- | --- | --- | --- | --- | --- |
| OTU000180 | Ascomycota | Dothideomycetes | Capnodiales | Neodevriesiaceae |  |  |
| OTU000181 | Mucoromycota | Endogonomycetes | Endogonales | Endogonaceae | Endogone |  |
| OTU000182 | Basidiomycota | Tremellomycetes | Tremellales | Trimorphomycetaceae | Saitozyma | Saitozyma_podzolica |
| OTU000183 | Ascomycota | Sordariomycetes | Sordariales | Lasiosphaeriaceae | Arnium |  |
| OTU000184 | Basidiomycota | Agaricomycetes | Agaricales | Entolomataceae | Clitopilus | Clitopilus_hobsonii |
| OTU000185 | Ascomycota |  |  |  |  |  |
| OTU000186 | Ascomycota | Sordariomycetes | Sordariales | Lasiosphaeriaceae | Arnium |  |
| OTU000187 | Ascomycota | Eurotiomycetes | Eurotiales | Aspergillaceae | Aspergillus | Aspergillus_penicillioides |
| OTU000188 | Basidiomycota | Agaricomycetes | Russulales | Russulaceae | Russula |  |
| OTU000189 | Ascomycota |  |  |  |  |  |
| OTU000190 | Basidiomycota | Agaricomycetes | Sebacinales | Serendipitaceae | Serendipita | Serendipita_indica |
| OTU000193 | Ascomycota | Sordariomycetes | Sordariales | Lasiosphaeriaceae |  |  |
| OTU000194 | Ascomycota | Sordariomycetes | Xylariales |  |  |  |
| OTU000195 | Ascomycota | Sordariomycetes | Hypocreales | Nectriaceae | Cylindrocarpon |  |
| OTU000196 | Basidiomycota | Agaricomycetes | Agaricales | Entolomataceae | Entoloma |  |
| OTU000197 | Ascomycota | Pezizomycetes | Pezizales | Morchellaceae | Morchella | Morchella_importuna |
| OTU000198 | Ascomycota | Sordariomycetes | Xylariales |  |  |  |
| OTU000200 | Ascomycota | Dothideomycetes | Pleosporales | Phaeosphaeriaceae | Leptospora |  |
| OTU000201 | Ascomycota | Saccharomycetes | Saccharomycetales | Debaryomycetaceae | Meyerozyma |  |
| OTU000202 | Basidiomycota | Ustilaginomycetes | Ustilaginales | Ustilaginaceae | Pseudozyma | Pseudozyma_hubeiensis |
| OTU000203 | Ascomycota | Sordariomycetes | Hypocreales | Bionectriaceae |  |  |
| OTU000204 | Ascomycota | Sordariomycetes | Sordariales | Lasiosphaeriaceae | Arnium |  |
| OTU000205 |  |  |  |  |  |  |
| OTU000206 | Ascomycota | Sordariomycetes |  |  |  |  |
| OTU000209 | Ascomycota | Eurotiomycetes | Chaetothyriales |  |  |  |
| OTU000210 | Mortierellomycota | Mortierellomycetes | Mortierellales | Mortierellaceae | Mortierella | Mortierella_elongata |
| OTU000211 | Ascomycota |  |  |  |  |  |
| OTU000213 | Ascomycota | Sordariomycetes | Chaetosphaeriales | Chaetosphaeriaceae | Dinemasporium | Dinemasporium_longicapillatum |
| OTU000215 | Ascomycota | Sordariomycetes | Hypocreales | Hypocreales_fam_Incertae_sedis | Ustilaginoidea | Ustilaginoidea_virens |
| OTU000216 | Mucoromycota | Mucoromycetes | Mucorales | Rhizopodaceae | Rhizopus | Rhizopus_arrhizus |
| OTU000217 | Ascomycota | Sordariomycetes | Hypocreales | Nectriaceae | Fusarium |  |
| OTU000218 | Ascomycota | Sordariomycetes | Sordariales | Chaetomiaceae | Acrophialophora | Acrophialophora_hechuanensis |
| OTU000219 | Ascomycota | Sordariomycetes | Sordariales | Chaetomiaceae |  |  |
| OTU000220 | Ascomycota | Saccharomycetes | Saccharomycetales | Debaryomycetaceae | Debaryomyces | Debaryomyces_hansenii |
| OTU000222 | Ascomycota | Dothideomycetes | Capnodiales | Cladosporiaceae | Cladosporium | Cladosporium_sphaerospermum |
| OTU000224 | Ascomycota | Eurotiomycetes | Eurotiales | Aspergillaceae | Aspergillus |  |
| OTU000225 | Ascomycota | Dothideomycetes | Capnodiales | Teratosphaeriaceae | Penidiella |  |

**Table S6** *Cont.*

| OTU ID | Phylum | Class | Order | Family | Genus | Species |
| --- | --- | --- | --- | --- | --- | --- |
| OTU000226 | Ascomycota | Eurotiomycetes | Chaetothyriales | Herpotrichiellaceae |  |  |
| OTU000229 | Ascomycota | Dothideomycetes |  |  |  |  |
| OTU000230 | Ascomycota | Sordariomycetes | Chaetosphaeriales | Chaetosphaeriaceae | Chloridium | Chloridium_aseptatum |
| OTU000231 | Ascomycota | Sordariomycetes |  |  |  |  |
| OTU000232 | Basidiomycota | Agaricomycetes | Agaricales | Inocybaceae | Inocybe | Inocybe_curvipes |
| OTU000234 | Ascomycota | Sordariomycetes | Sordariales | Chaetomiaceae | Trichocladium | Trichocladium_pyriforme |
| OTU000235 |  |  |  |  |  |  |
| OTU000236 | Ascomycota | Sordariomycetes | Sordariales | Chaetomiaceae | Humicola | Humicola_grisea |
| OTU000237 | Ascomycota |  |  |  |  |  |
| OTU000238 | Ascomycota | Sordariomycetes | Chaetosphaeriales | Chaetosphaeriaceae | Dinemasporium | Dinemasporium_longicapillatum |
| OTU000239 | Ascomycota | Dothideomycetes | Capnodiales | Teratosphaeriaceae | Devriesia | Devriesia_pseudoamericana |
| OTU000240 | Ascomycota | Eurotiomycetes | Eurotiales | Aspergillaceae | Aspergillus | Aspergillus_thermomutatus |
| OTU000241 | Ascomycota | Sordariomycetes | Sordariales |  |  |  |
| OTU000243 | Ascomycota | Sordariomycetes | Microascales | Microascaceae |  |  |
| OTU000244 | Ascomycota | Eurotiomycetes | Eurotiales | Aspergillaceae | Aspergillus |  |
| OTU000245 | Ascomycota | Dothideomycetes | Pleosporales | Didymosphaeriaceae |  |  |
| OTU000246 | Basidiomycota | Malasseziomycetes | Malasseziales | Malasseziaceae | Malassezia |  |
| OTU000247 | Ascomycota | Eurotiomycetes | Eurotiales | Aspergillaceae | Aspergillus | Aspergillus_penicillioides |
| OTU000248 | Basidiomycota |  |  |  |  |  |
| OTU000249 | Basidiomycota | Microbotryomycetes | Sporidiobolales | Sporidiobolaceae | Rhodotorula | Rhodotorula_diobovata |
| OTU000250 | Ascomycota | Dothideomycetes | Pleosporales | Periconiaceae | Periconia |  |
| OTU000251 | Ascomycota |  |  |  |  |  |
| OTU000252 | Ascomycota | Sordariomycetes | Xylariales |  |  |  |
| OTU000253 | Ascomycota | Sordariomycetes | Microascales | Microascaceae | Scedosporium |  |
| OTU000255 | Ascomycota | Sordariomycetes | Sordariales | Sordariales_fam_Incertae_sedis | Staphylotrichum |  |
| OTU000256 | Ascomycota | Eurotiomycetes | Eurotiales | Aspergillaceae | Penicillium |  |
| OTU000257 | Ascomycota |  |  |  |  |  |
| OTU000258 | Basidiomycota | Agaricomycetes | Agaricales | Inocybaceae | Inocybe |  |
| OTU000259 | Basidiomycota | Agaricomycetes | Geastrales |  |  |  |
| OTU000260 |  |  |  |  |  |  |
| OTU000261 | Ascomycota | Sordariomycetes | Sordariales | Lasiosphaeriaceae | Schizothecium |  |
| OTU000262 | Ascomycota |  |  |  |  |  |
| OTU000264 |  |  |  |  |  |  |
| OTU000265 | Basidiomycota | Agaricomycetes | Agaricales | Bolbitiaceae | Conocybe |  |
| OTU000266 | Ascomycota | Sordariomycetes | Hypocreales | Clavicipitaceae | Metarhizium | Metarhizium_lepidiotae |
| OTU000267 | Mucoromycota | Endogonomycetes | Endogonales |  |  |  |
| OTU000268 | Basidiomycota | Agaricomycetes | Cantharellales | Ceratobasidiaceae |  |  |

**Table S6** *Cont.*

| OTU ID | Phylum | Class | Order | Family | Genus | Species |
| --- | --- | --- | --- | --- | --- | --- |
| OTU000270 | Basidiomycota | Agaricomycetes | Polyporales | Fomitopsidaceae |  |  |
| OTU000271 | Ascomycota | Eurotiomycetes | Eurotiales | Aspergillaceae | Penicillium |  |
| OTU000272 | Mortierellomycota | Mortierellomycetes | Mortierellales | Mortierellaceae | Mortierella | Mortierella_exigua |
| OTU000273 | Basidiomycota | Wallemiomycetes | Wallemiales | Wallemiaceae | Wallemia |  |
| OTU000275 | Ascomycota | Sordariomycetes | Sordariales | Chaetomiaceae |  |  |
| OTU000278 | Basidiomycota | Tremellomycetes | Tremellales | Rhynchogastremataceae | Papiliotrema | Papiliotrema_japonica |
| OTU000279 | Basidiomycota | Agaricomycetes | Agaricales |  |  |  |
| OTU000280 | Ascomycota | Pezizomycetes | Pezizales | Pyronemataceae |  |  |
| OTU000282 | Ascomycota | Leotiomycetes | Helotiales | Helotiaceae |  |  |
| OTU000284 | Ascomycota | Eurotiomycetes | Onygenales | Onygenales_fam_Incertae_sedis | Chrysosporium | Chrysosporium_pseudomerdarium |
| OTU000285 | Ascomycota |  |  |  |  |  |
| OTU000286 | Ascomycota |  |  |  |  |  |
| OTU000287 | Ascomycota | Saccharomycetes | Saccharomycetales |  |  |  |
| OTU000288 | Ascomycota | Dothideomycetes | Dothideales | Aureobasidiaceae | Aureobasidium | Aureobasidium_thailandense |
| OTU000290 |  |  |  |  |  |  |
| OTU000291 | Basidiomycota | Agaricomycetes | Agaricales | Schizophyllaceae | Schizophyllum | Schizophyllum_commune |
| OTU000292 | Ascomycota | Saccharomycetes | Saccharomycetales | Saccharomycodaceae | Hanseniaspora | Hanseniaspora_osmophila |
| OTU000295 | Ascomycota | Dothideomycetes | Pleosporales |  |  |  |
| OTU000296 | Ascomycota |  |  |  |  |  |
| OTU000297 | Basidiomycota | Agaricomycetes | Agaricales |  |  |  |
| OTU000300 | Mortierellomycota | Mortierellomycetes | Mortierellales | Mortierellaceae | Mortierella |  |
| OTU000302 | Ascomycota | Eurotiomycetes | Eurotiales | Aspergillaceae | Aspergillus |  |
| OTU000306 | Basidiomycota | Tremellomycetes | Trichosporonales | Trichosporonaceae | Cutaneotrichosporon |  |
| OTU000308 | Ascomycota | Sordariomycetes | Sordariales |  |  |  |
| OTU000309 | Basidiomycota | Tremellomycetes | Trichosporonales | Trichosporonaceae | Trichosporon |  |
| OTU000311 | Ascomycota | Saccharomycetes | Saccharomycetales | Metschnikowiaceae | Metschnikowia |  |
| OTU000312 | Ascomycota | Saccharomycetes | Saccharomycetales | Saccharomycetaceae | Torulaspora | Torulaspora_delbrueckii |
| OTU000314 | Basidiomycota | Agaricomycetes |  |  |  |  |
| OTU000315 | Mortierellomycota | Mortierellomycetes | Mortierellales | Mortierellaceae | Mortierella |  |
| OTU000316 | Ascomycota | Sordariomycetes | Magnaporthales | Magnaporthaceae | Arxiella | Arxiella_dolichandrae |
| OTU000318 | Basidiomycota | Tremellomycetes | Filobasidiales | Filobasidiaceae | Filobasidium |  |
| OTU000319 | Ascomycota | Sordariomycetes | Sordariales | Lasiosphaeriaceae | Podospora | Podospora_pyriformis |
| OTU000320 |  |  |  |  |  |  |
| OTU000321 | Ascomycota |  |  |  |  |  |
| OTU000322 | Mortierellomycota | Mortierellomycetes | Mortierellales | Mortierellaceae | Mortierella | Mortierella_alpina |
| OTU000323 | Ascomycota | Saccharomycetes | Saccharomycetales | Saccharomycetales_fam_Incertae_sedis |  |  |
| OTU000324 | Basidiomycota | Wallemiomycetes | Wallemiales | Wallemiaceae | Wallemia | Wallemia_canadensis |

**Table S6** *Cont.*

| OTU ID | Phylum | Class | Order | Family | Genus | Species |
| --- | --- | --- | --- | --- | --- | --- |
| OTU000325 | Ascomycota | Eurotiomycetes | Eurotiales | Aspergillaceae | Monascus | Monascus_purpureus |
| OTU000326 | Ascomycota | Sordariomycetes | Glomerellales | Glomerellaceae | Colletotrichum |  |
| OTU000327 | Ascomycota | Sordariomycetes | Hypocreales | Nectriaceae | Fusarium | Fusarium_chlamydosporum |
| OTU000328 | Ascomycota | Saccharomycetes | Saccharomycetales | Metschnikowiaceae | Metschnikowia |  |
| OTU000330 | Ascomycota | Sordariomycetes | Xylariales | Apiosporaceae | Arthrinium |  |
| OTU000331 | Ascomycota | Dothideomycetes | Pleosporales | Phaeosphaeriaceae | Neosetophoma |  |
| OTU000333 | Ascomycota | Sordariomycetes | Chaetosphaeriales | Chaetosphaeriaceae |  |  |
| OTU000334 | Ascomycota | Saccharomycetes | Saccharomycetales | Dipodascaceae | Dipodascus | Dipodascus_geotrichum |
| OTU000335 | Ascomycota | Saccharomycetes | Saccharomycetales | Saccharomycodaceae | Hanseniaspora | Hanseniaspora_vineae |
| OTU000339 | Basidiomycota | Agaricomycetes | Cantharellales | Ceratobasidiaceae | Ceratobasidium |  |
| OTU000340 |  |  |  |  |  |  |
| OTU000341 | Ascomycota | Leotiomycetes | Erysiphales | Erysiphaceae | Erysiphe | Erysiphe_necator |
| OTU000342 | Ascomycota |  |  |  |  |  |
| OTU000344 | Ascomycota | Dothideomycetes | Pleosporales | Didymosphaeriaceae |  |  |
| OTU000345 | Basidiomycota | Agaricomycetes | Polyporales |  |  |  |
| OTU000347 | Basidiomycota | Agaricomycetes | Agaricales | Entolomataceae | Entoloma | Entoloma_clypeatum |
| OTU000348 | Ascomycota | Dothideomycetes | Pleosporales | Didymosphaeriaceae | Pseudopithomyces | Pseudopithomyces_rosae |
| OTU000350 | Mortierellomycota | Mortierellomycetes | Mortierellales | Mortierellaceae | Mortierella | Mortierella_zychae |
| OTU000351 | Ascomycota | Dothideomycetes | Pleosporales | Torulaceae | Dendryphion | Dendryphion_fluminicola |
| OTU000352 | Ascomycota |  |  |  |  |  |
| OTU000353 | Ascomycota | Pezizomycetes | Pezizales | Pezizaceae |  |  |
| OTU000354 |  |  |  |  |  |  |
| OTU000355 | Ascomycota | Eurotiomycetes | Eurotiales | Aspergillaceae | Aspergillus | Aspergillus_halophilicus |
| OTU000356 | Ascomycota | Pezizomycetes | Pezizales | Pyronemataceae | Pseudaleuria |  |
| OTU000358 | Basidiomycota | Agaricomycetes | Agaricales | Lyophyllaceae |  |  |
| OTU000360 | Ascomycota | Dothideomycetes | Pleosporales | Corynesporascaceae | Corynespora |  |
| OTU000363 | Ascomycota | Pezizomycetes | Pezizales | Pyronemataceae | Pseudaleuria |  |
| OTU000370 | Ascomycota | Leotiomycetes | Helotiales | Leotiaceae | Neobulgaria |  |
| OTU000373 | Basidiomycota | Ustilaginomycetes | Ustilaginales | Ustilaginaceae |  |  |
| OTU000374 | Ascomycota | Sordariomycetes | Pleurotheciales | Pleurotheciaceae | Phaeoisaria |  |
| OTU000375 | Ascomycota | Eurotiomycetes | Eurotiales | Trichocomaceae | Talaromyces |  |
| OTU000377 | Mortierellomycota | Mortierellomycetes | Mortierellales | Mortierellaceae | Mortierella | Mortierella_fatshederae |
| OTU000378 | Ascomycota | Sordariomycetes | Sordariales | Lasiosphaeriaceae | Cercophora |  |
| OTU000379 | Mucoromycota | Mucoromycetes | Mucorales | Mucoraceae | Mucor | Mucor_nidicola |
| OTU000380 | Basidiomycota | Cystobasidiomycetes | Cystobasidiales | Cystobasidiaceae | Cystobasidium | Cystobasidium_minuta |
| OTU000385 | Basidiomycota | Cystobasidiomycetes | Cystobasidiales | Cystobasidiaceae | Occultifur |  |
| OTU000386 | Ascomycota | Dothideomycetes | Pleosporales | Pleosporaceae | Curvularia |  |

**Table S6** *Cont.*

| OTU ID | Phylum | Class | Order | Family | Genus | Species |
| --- | --- | --- | --- | --- | --- | --- |
| OTU000387 | Ascomycota | Sordariomycetes |  |  |  |  |
| OTU000388 | Ascomycota | Sordariomycetes | Hypocreales | Nectriaceae | Fusarium | Fusarium_concentricum |
| OTU000389 |  |  |  |  |  |  |
| OTU000391 | Ascomycota | Sordariomycetes | Hypocreales | Nectriaceae | Fusarium |  |
| OTU000392 | Ascomycota |  |  |  |  |  |
| OTU000394 | Ascomycota | Sordariomycetes | Hypocreales | Hypocreales_fam_Incertae_sedis |  |  |
| OTU000395 | Ascomycota | Dothideomycetes | Pleosporales | Sporormiaceae |  |  |
| OTU000397 |  |  |  |  |  |  |
| OTU000398 | Ascomycota | Sordariomycetes | Sordariales | Lasiosphaeriaceae |  |  |
| OTU000401 |  |  |  |  |  |  |
| OTU000404 | Ascomycota | Sordariomycetes | Hypocreales | Nectriaceae |  |  |
| OTU000405 | Ascomycota |  |  |  |  |  |
| OTU000406 | Ascomycota | Sordariomycetes | Boliniales | Boliniaceae | Endoxyla |  |
| OTU000407 | Ascomycota | Sordariomycetes | Hypocreales | Cordycipitaceae | Engyodontium | Engyodontium_album |
| OTU000409 | Ascomycota | Sordariomycetes | Xylariales | Xylariaceae | Xylaria |  |
| OTU000410 | Ascomycota | Saccharomycetes | Saccharomycetales | Debaryomycetaceae | Schwanniomyces | Schwanniomyces_occidentalis |
| OTU000411 | Ascomycota | Pezizomycetes | Pezizales | Pezizaceae | Chromelosporium | Chromelosporium_macrospermum |
| OTU000412 | Ascomycota |  |  |  |  |  |
| OTU000413 | Ascomycota | Sordariomycetes | Hypocreales | Nectriaceae | Xenoacremonium | Xenoacremonium_recifei |
| OTU000414 | Basidiomycota | Agaricomycetes |  |  |  |  |
| OTU000415 | Ascomycota | Dothideomycetes | Capnodiales | Teratosphaeriaceae | Devriesia | Devriesia_pseudoamericana |
| OTU000417 | Ascomycota | Leotiomycetes | Helotiales |  |  |  |
| OTU000418 | Ascomycota | Pezizomycetes | Pezizales | Pezizaceae | Iodophanus |  |
| OTU000419 | Ascomycota | Sordariomycetes | Xylariales | Microdochiaceae | Idriella |  |
| OTU000422 | Ascomycota | Sordariomycetes | Hypocreales | Nectriaceae | Fusarium |  |
| OTU000423 | Ascomycota | Dothideomycetes | Capnodiales |  |  |  |
| OTU000424 | Ascomycota | Sordariomycetes | Sordariales | Lasiosphaeriaceae |  |  |
| OTU000425 | Ascomycota | Sordariomycetes |  |  |  |  |
| OTU000426 | Ascomycota | Sordariomycetes | Sordariales | Lasiosphaeriaceae |  |  |
| OTU000427 | Ascomycota | Sordariomycetes | Glomerellales | Plectosphaerellaceae | Chordomyces | Chordomyces_antarcticus |
| OTU000428 | Basidiomycota | Malasseziomycetes | Malasseziales | Malasseziaceae | Malassezia | Malassezia_globosa |
| OTU000429 | Ascomycota | Dothideomycetes | Pleosporales | Pleosporaceae | Exserohilum | Exserohilum_rostratum |
| OTU000430 | Basidiomycota | Agaricomycetes |  |  |  |  |
| OTU000432 | Basidiomycota | Geminibasidiomycetes | Geminibasidiales | Geminibasidiaceae | Geminibasidium |  |
| OTU000434 | Basidiomycota | Agaricomycetes | Agaricales | Lycoperdaceae |  |  |
| OTU000435 | Ascomycota | Dothideomycetes | Pleosporales |  |  |  |
| OTU000436 | Ascomycota |  |  |  |  |  |

**Table S6** *Cont.*

| OTU ID | Phylum | Class | Order | Family | Genus | Species |
| --- | --- | --- | --- | --- | --- | --- |
| OTU000437 | Basidiomycota | Agaricomycetes | Polyporales | Polyporaceae | Trametes |  |
| OTU000438 | Ascomycota | Sordariomycetes |  |  |  |  |
| OTU000439 | Ascomycota | Sordariomycetes | Microascales | Microascaceae | Cephalotrichum |  |
| OTU000440 | Ascomycota | Dothideomycetes | Pleosporales | Pleosporaceae | Stemphylium |  |
| OTU000441 | Ascomycota | Sordariomycetes | Trichosphaeriales | Trichosphaeriaceae | Nigrospora |  |
| OTU000443 | Mortierellomycota | Mortierellomycetes | Mortierellales | Mortierellaceae | Mortierella |  |
| OTU000447 | Ascomycota | Leotiomycetes | Helotiales |  |  |  |
| OTU000448 | Basidiomycota | Agaricomycetes | Agaricales | Stephanosporaceae |  |  |
| OTU000450 | Basidiomycota | Agaricomycetes | Agaricales | Agaricaceae | Lepiota | Lepiota_venenata |
| OTU000452 | Ascomycota | Leotiomycetes | Helotiales | Helotiaceae | Scytalidium | Scytalidium_lignicola |
| OTU000453 | Basidiomycota |  |  |  |  |  |
| OTU000454 | Ascomycota | Saccharomycetes | Saccharomycetales | Phaffomycetaceae | Wickerhamomyces | Wickerhamomyces_anomalus |
| OTU000455 | Ascomycota | Dothideomycetes | Pleosporales | Phaeosphaeriaceae | Paraphoma |  |
| OTU000458 | Ascomycota | Leotiomycetes | Helotiales | Helotiaceae | Scytalidium | Scytalidium_lignicola |
| OTU000460 | Basidiomycota | Agaricomycetes | Hymenochaetales | Schizoporaceae | Hyphodontia | Hyphodontia_microspora |
| OTU000461 | Basidiomycota |  |  |  |  |  |
| OTU000462 | Ascomycota | Leotiomycetes | Helotiales | Helotiaceae | Hymenoscyphus | Hymenoscyphus_varicosporoides |
| OTU000464 | Chytridiomycota | Spizellomycetes | Spizellomycetales | Powellomycetaceae | Powellomyces | Powellomyces_hirtus |
| OTU000467 | Ascomycota | Dothideomycetes | Dothideales | Aureobasidiaceae | Aureobasidium |  |
| OTU000468 | Ascomycota |  |  |  |  |  |
| OTU000469 | Ascomycota | Sordariomycetes | Xylariales | Xylariales_fam_Incertae_sedis | Hansfordia | Hansfordia_pulvinata |
| OTU000470 | Ascomycota | Sordariomycetes | Hypocreales | Hypocreaceae | Trichoderma |  |
| OTU000473 | Ascomycota | Sordariomycetes | Hypocreales | Nectriaceae | Fusarium | Fusarium_concentricum |
| OTU000476 | Basidiomycota | Agaricomycetes | Polyporales | Hyphodermataceae | Hyphoderma | Hyphoderma_subsetigerum |
| OTU000478 | Ascomycota |  |  |  |  |  |
| OTU000479 | Mortierellomycota | Mortierellomycetes | Mortierellales | Mortierellaceae | Mortierella |  |
| OTU000481 | Ascomycota | Sordariomycetes | Xylariales | Xylariaceae | Xylaria | Xylaria_feejeensis |
| OTU000483 | Basidiomycota | Cystobasidiomycetes | Cystobasidiales | Cystobasidiaceae | Cystobasidium | Cystobasidium_calyptogenae |
| OTU000485 | Basidiomycota | Agaricomycetes | Agaricales | Lycoperdaceae | Lycoperdon |  |
| OTU000489 | Mortierellomycota | Mortierellomycetes | Mortierellales | Mortierellaceae | Mortierella | Mortierella_ambigua |
| OTU000490 | Basidiomycota | Agaricomycetes | Polyporales | Phanerochaetaceae |  |  |
| OTU000491 | Ascomycota | Dothideomycetes | Pleosporales | Sporormiaceae |  |  |
| OTU000493 | Ascomycota | Sordariomycetes | Xylariales | Diatrypaceae |  |  |
| OTU000494 | Ascomycota | Sordariomycetes | Trichosphaeriales | Trichosphaeriaceae | Nigrospora | Nigrospora_oryzae |
| OTU000499 | Ascomycota | Leotiomycetes | Thelebolales | Pseudeurotiaceae | Pseudeurotium |  |
| OTU000501 | Ascomycota | Dothideomycetes | Capnodiales |  |  |  |
| OTU000502 |  |  |  |  |  |  |

**Table S6** *Cont.*

| OTU ID | Phylum | Class | Order | Family | Genus | Species |
| --- | --- | --- | --- | --- | --- | --- |
| OTU000506 | Basidiomycota | Agaricomycetes | Polyporales | Polyporaceae | Hexagonia | Hexagonia_apiaria |
| OTU000508 | Basidiomycota | Malasseziomycetes | Malasseziales | Malasseziaceae | Malassezia |  |
| OTU000509 | Ascomycota | Sordariomycetes | Microascales | Microascaceae | Cephalotrichum | Cephalotrichum_telluricum |
| OTU000510 | Ascomycota | Saccharomycetes | Saccharomycetales | Saccharomycetales_fam_Incertae_sedis | Candida | Candida_zeylanoides |
| OTU000511 | Ascomycota |  |  |  |  |  |
| OTU000512 | Ascomycota | Eurotiomycetes | Eurotiales | Aspergillaceae | Penicillium |  |
| OTU000513 |  |  |  |  |  |  |
| OTU000514 | Mucoromycota | Mucoromycetes | Mucorales | Mucoraceae | Mucor | Mucor_circinelloides |
| OTU000519 | Basidiomycota |  |  |  |  |  |
| OTU000520 | Ascomycota |  |  |  |  |  |
| OTU000524 | Ascomycota |  |  |  |  |  |
| OTU000525 | Ascomycota | Saccharomycetes | Saccharomycetales | Saccharomycetaceae | Kazachstania | Kazachstania_exigua |
| OTU000527 | Ascomycota | Saccharomycetes | Saccharomycetales | Pichiaceae | Pichia |  |
| OTU000528 | Basidiomycota | Malasseziomycetes | Malasseziales | Malasseziaceae | Malassezia | Malassezia_globosa |
| OTU000530 | Basidiomycota | Agaricomycetes | Geastrales | Geastraceae | Geastrum | Geastrum_schmidelii |
| OTU000531 | Ascomycota | Saccharomycetes | Saccharomycetales | Saccharomycetaceae | Zygosaccharomyces | Zygosaccharomyces_bisporus |
| OTU000535 | Ascomycota | Sordariomycetes | Diaporthales | Valsaceae |  |  |
| OTU000539 | Ascomycota | Dothideomycetes | Capnodiales | Cladosporiaceae | Cladosporium |  |
| OTU000543 | Ascomycota | Pezizomycetes | Pezizales | Morchellaceae | Morchella | Morchella_septimelata |
| OTU000548 | Ascomycota | Sordariomycetes | Pleurotheciales | Pleurotheciaceae |  |  |
| OTU000549 | Rozellomycota | Rozellomycotina_cls_Incertae_sedis | GS11 |  |  |  |
| OTU000550 | Ascomycota | Saccharomycetes | Saccharomycetales | Saccharomycetaceae | Zygosaccharomyces | Zygosaccharomyces_bailii |
| OTU000551 | Ascomycota | Dothideomycetes | Pleosporales | Pleosporaceae | Alternaria |  |
| OTU000554 | Basidiomycota | Agaricomycetes | Agaricales | Physalacriaceae | Physalacria | Physalacria_maipoensis |
| OTU000555 | Mortierellomycota | Mortierellomycetes | Mortierellales | Mortierellaceae | Mortierella | Mortierella_kuhlmanii |
| OTU000556 |  |  |  |  |  |  |
| OTU000557 | Basidiomycota | Agaricomycetes | Polyporales | Phanerochaetaceae |  |  |
| OTU000558 | Basidiomycota | Microbotryomycetes | Sporidiobolales | Sporidiobolaceae | Rhodotorula | Rhodotorula_mucilaginosa |
| OTU000559 | Basidiomycota | Agaricomycetes | Cantharellales | Ceratobasidiaceae | Thanatephorus | Thanatephorus_cucumeris |
| OTU000561 | Mucoromycota | Umbelopsidomycetes | Umbelopsidales | Umbelopsidaceae | Umbelopsis | Umbelopsis_dimorpha |
| OTU000562 | Ascomycota | Eurotiomycetes | Eurotiales | Aspergillaceae | Penicillium |  |
| OTU000564 | Ascomycota | Dothideomycetes | Pleosporales | Leptosphaeriaceae | Leptosphaeria | Leptosphaeria_sclerotioides |
| OTU000565 | Ascomycota |  |  |  |  |  |
| OTU000566 | Glomeromycota | Glomeromycetes | Glomerales | Glomeraceae | Glomus | Glomus_indicum |
| OTU000569 | Ascomycota | Dothideomycetes | Capnodiales | Dissoconiaceae | Dissoconium |  |
| OTU000571 | Ascomycota | Eurotiomycetes | Eurotiales | Aspergillaceae | Aspergillus |  |
| OTU000573 | Basidiomycota | Tremellomycetes | Tremellales | Bulleribasidiaceae | Vishniacozyma | Vishniacozyma_victoriae |

**Table S6** *Cont.*

| OTU ID | Phylum | Class | Order | Family | Genus | Species |
| --- | --- | --- | --- | --- | --- | --- |
| OTU000574 | Ascomycota | Eurotiomycetes | Eurotiales | Trichocomaceae | Sagenomella |  |
| OTU000576 | Ascomycota | Sordariomycetes | Glomerellales | Plectosphaerellaceae | Plectosphaerella |  |
| OTU000577 | Basidiomycota | Ustilaginomycetes | Ustilaginales | Ustilaginaceae |  |  |
| OTU000579 | Ascomycota | Saccharomycetes | Saccharomycetales | Saccharomycetaceae |  |  |
| OTU000580 | Ascomycota | Sordariomycetes | Xylariales | Sporocadaceae | Neopestalotiopsis |  |
| OTU000581 | Basidiomycota | Agaricomycetes |  |  |  |  |
| OTU000582 | Ascomycota |  |  |  |  |  |
| OTU000583 | Ascomycota | Saccharomycetes | Saccharomycetales | Phaffomycetaceae | Cyberlindnera | Cyberlindnera_fabianii |
| OTU000584 | Glomeromycota | Glomeromycetes | Glomerales | Glomeraceae |  |  |
| OTU000585 | Basidiomycota | Agaricomycetes | Russulales | Auriscalpiaceae | Lentinellus |  |
| OTU000589 | Ascomycota | Pezizomycetes | Pezizales | Pyronemataceae |  |  |
| OTU000590 | Ascomycota | Saccharomycetes | Saccharomycetales | Saccharomycodaceae | Hanseniaspora |  |
| OTU000591 | Basidiomycota | Agaricomycetes |  |  |  |  |
| OTU000592 | Ascomycota | Saccharomycetes | Saccharomycetales |  |  |  |
| OTU000593 | Glomeromycota | Glomeromycetes | Glomerales | Glomeraceae |  |  |
| OTU000595 | Basidiomycota | Agaricomycetes | Geastrales | Geastraceae | Geastrum | Geastrum_floriforme |
| OTU000597 | Mortierellomycota | Mortierellomycetes | Mortierellales | Mortierellaceae | Mortierella |  |
| OTU000598 |  |  |  |  |  |  |
| OTU000600 | Ascomycota | Dothideomycetes | Pleosporales | Phaeosphaeriaceae |  |  |
| OTU000601 | Mortierellomycota | Mortierellomycetes | Mortierellales | Mortierellaceae | Mortierella | Mortierella_elongata |
| OTU000602 | Ascomycota | Dothideomycetes | Pleosporales | Didymellaceae |  |  |
| OTU000603 | Ascomycota | Eurotiomycetes | Chaetothyriales |  |  |  |

Table S7 Alpha diversity of benthic fungal communities

| Sample name | Shannon | Chao1 | Pielou |
| --- | --- | --- | --- |
| A1-1 | 4.23078615179821 | 236.545454545455 | 0.7817928429277 |
| A1-2 | 3.0340832949729 | 137 | 0.621381604881031 |
| A1-3 | 3.47271567147546 | 130 | 0.721650588385803 |
| B3-1 | 0.755977091496576 | 133.1 | 0.156832558680976 |
| B3-2 | 0.753347109394823 | 121.75 | 0.159358008753346 |
| B3-3 | 0.533929175413264 | 163.666666666667 | 0.114725767999977 |
| C2-1 | 3.38991097570574 | 225.833333333333 | 0.644776787392259 |
| C2-2 | 2.979661485 | 212.916666666667 | 0.564531405127981 |
| C2-3 | 3.92184881938685 | 223.5 | 0.745953831981066 |
| C3-1 | 3.78332846826425 | 213.555555555556 | 0.712061321015557 |
| C3-2 | 3.55691199021012 | 175.083333333333 | 0.711779247178327 |
| C3-3 | 3.72128531258592 | 206.071428571429 | 0.720511039832883 |
| M0-1 | 2.46669187423858 | 107.4 | 0.532219225871241 |
| M0-2 | 2.70584045411475 | 95.5 | 0.599850305735662 |
| M0-3 | 2.89172563025819 | 165.176470588235 | 0.597923298495428 |
